# Stable and Promiscuous Galactose Oxidases Engineered by Directed Evolution, Atomistic Design, and Ancestral Sequence Reconstruction

**DOI:** 10.1021/acssynbio.4c00653

**Published:** 2024-12-13

**Authors:** Merve Keser, Ivan Mateljak, Roman Kittl, Roland Ludwig, Valeria A. Risso, Jose M. Sanchez-Ruiz, David Gonzalez-Perez, Miguel Alcalde

**Affiliations:** † Department of Biocatalysis, Institute of Catalysis, ICP-CSIC, 28049 Madrid, Spain; ‡ EvoEnzyme S.L., Parque Científico de Madrid, 28049 Madrid, Spain; § DirectSens GmbH, Am Rosenbühel 38, 3400 Klosterneuburg, Austria; ∥ Department of Food Science and Technology, Institute of Food Technology, University of Natural Resources and Life Sciences, Muthgasse 18, 1190 Vienna, Austria; ⊥ Departamento de Quimica Fisica, Facultad de Ciencias, Unidad de Excelencia de Quimica Aplicada a Biomedicina y Medioambiente (UEQ), Universidad de Granada, 18071 Granada, Spain

**Keywords:** galactose oxidase, directed evolution, PROSS atomistic design, ancestral sequence reconstruction, thermostability, expression, promiscuity

## Abstract

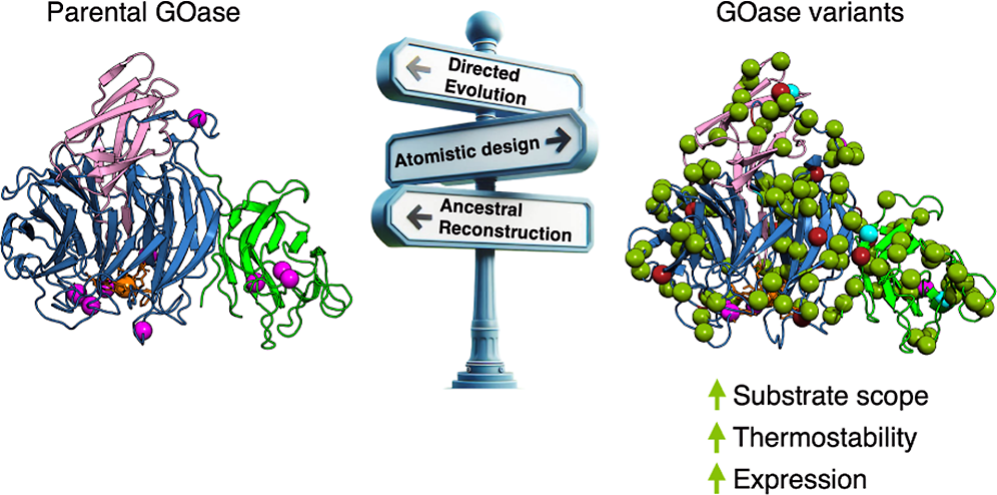

Galactose oxidase (GOase) is a versatile biocatalyst with a wide range of potential applications, ranging from synthetic chemistry to bioelectrochemical devices. Previous GOase engineering by directed evolution generated the M-RQW mutant, with unprecedented new-to-nature oxidation activity at the C6-OH group of glucose, and a mutational backbone that helped to unlock its promiscuity toward other molecules, including secondary alcohols. In the current study, we have used the M-RQW mutant as a starting point to engineer a set of GOases that are very thermostable and that are easily produced at high titers in yeast, enzymes with latent activities applicable to sustainable chemistry. To boost the generation of sequence and functional diversity, the directed evolution workflow incorporated one-shot computational mutagenesis by the PROSS algorithm and ancestral sequence reconstruction. This synergetic approach helped produce a rapid rise in functional expression by *Pichia pastoris*, achieving g/L production in a fed-batch bioreactor while the different GOases designed were resistant to pH and high temperature, with *T*_50_ enhancements up to 27 °C over the parental M-RQW. These designs displayed latent activity against glucose and an array of secondary aromatic alcohols with different degrees of bulkiness, becoming a suitable point of departure for the future engineering of industrial GOases.

## Introduction

Galactose oxidase (E.C. 1.1.3.9, d-galactose:oxygen 6-oxidoreductase, GOase) is an extracellular monocopper-dependent enzyme characterized by its natural oxidation of the C6-OH group of d-galactose to the corresponding aldehyde, while reducing O_2_ to H_2_O_2_. Wild-type GOase (GOase wt) enzymes are secreted by some fungal species and also act on raffinose, lactose, oligo and polysaccharides, and primary alcohols.^1^ This interesting substrate scope, coupled to its singular electrochemical features, makes GOase a promising biocatalyst for applications that range from biosensor development to chemical synthesis.^2^ For several years, GOase has been in the sights of protein engineers, attempting to convert it into an industrial biocatalyst. Among their most important achievements were the foundational studies carried out by the Arnold group in which GOase underwent two consecutive directed evolution campaigns to enhance its functional expression in bacteria and to unlock its substrate promiscuity.^3,4^ The latter was particularly noteworthy, as the enzyme was designed to selectively oxidize glucose at C6-OH, an activity not found in nature. The outcome of this laboratory evolution was the M-RQW variant, which carries five mutations driving expression in *Escherichia coli* (S10P, M70V, G195E, V494A, and N535D), as well as mutations in the backbone responsible for the novel activity on glucose (W290F, R330K, and Q406T).^4^

An unexpected consequence of this study was the appearance of latent activities in the M-RQW mutant toward primary alcohols with carbonyl or aromatic moieties at the α-position, as well as a weak yet noticeable activity on secondary alcohols. From this starting point, the enzyme mutant was engineered to achieve kinetic resolution of a broad panel of secondary alcohols.^5^ Subsequently, this substrate scope was further expanded toward bulky benzylic and alkyl secondary alcohols by structure-guided directed evolution.^6^ This repertoire of mutants also proved to be a solid departure point for other complex transformations, spanning from the selective oxidation of 5-hydroxymethylfurfural and glycans, to the synthesis of nitriles from primary alcohols, showcasing the immense plasticity of M-RQW and the variants thereof.^7−9^

The emergence of new computational methods for protein engineering expands the potential for the laboratory design of enzymes. Among the most appealing approaches are those based on atomistic and phylogenetic calculations, which can open up unexplored avenues when combined with directed evolution.^10^ In this regard, our group is bringing together directed evolution with one-shot computational mutagenesis based on Rosetta design and phylogenetic inference (PROSS and FuncLib algorithms) with a view to generate functional designs with a repertoire of enhanced features, including expressibility, thermostability, and enantiodivergence.^11−13^ Likewise, ancestral sequence reconstruction is useful to learn more about primitive protein function and when combined with directed evolution may extend the biochemical features of modern counterparts.^14−18^ This protein engineering toolbox places us in a good position to engineer new variants of M-RQW with a view to generate more robust and functionally diverse GOases.

In this study, we describe how combining directed evolution, atomistic design calculations, and ancestral sequence reconstruction led to the design of highly stable and promiscuous GOases. The final GOases obtained are readily secreted by yeast on a gram per liter scale in a fed-batch bioreactor, and their strong improvements in thermostability are coupled to latent activities that could be targeted in future protein engineering endeavors.

## Results and Discussion

### Directed Evolution and Atomistic Design

**Figure 1:**
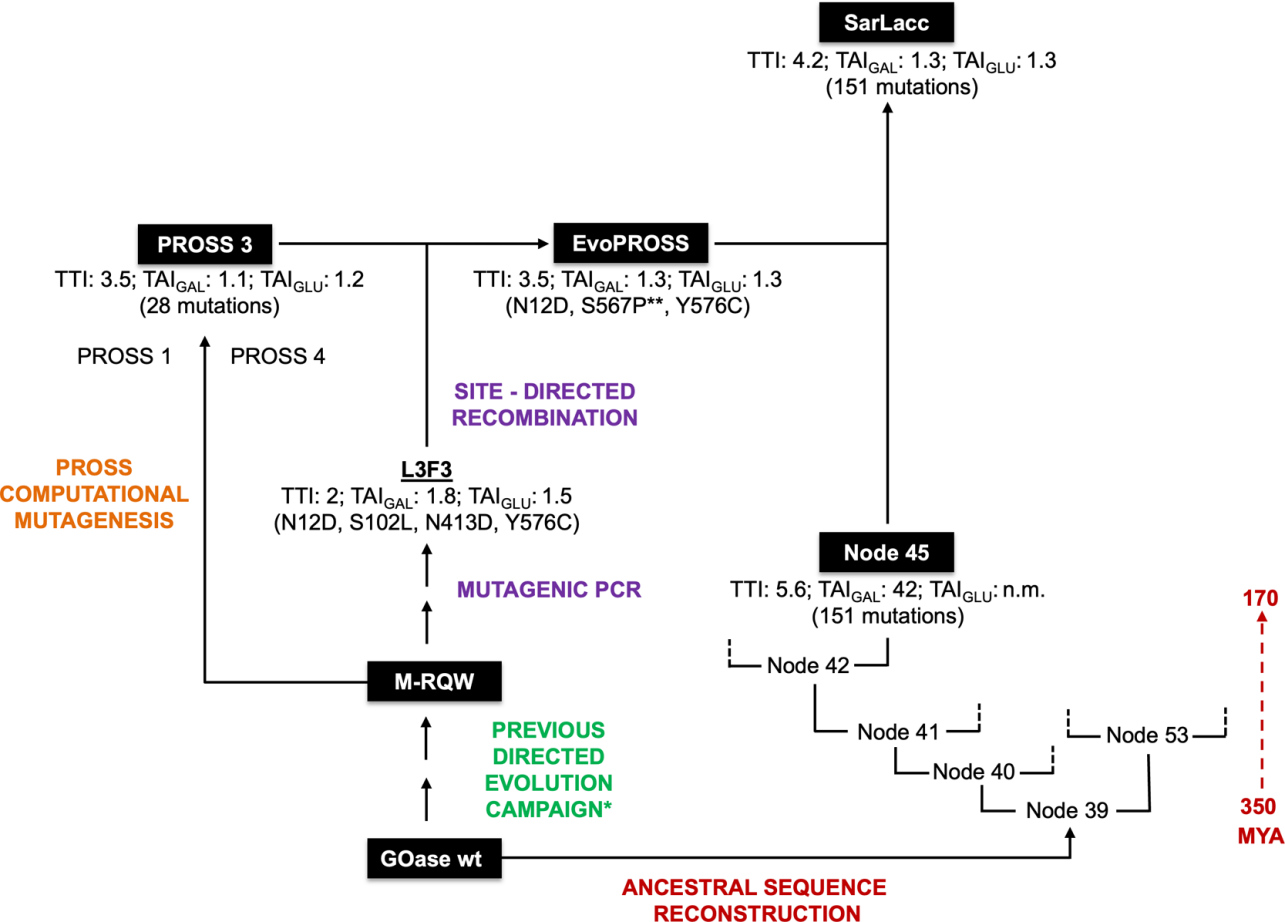
Overview of the GOase engineering strategy of this work. The M-RQW mutant was the starting point in this study, derived from a previous directed evolution campaign* aimed at improving functional expression in *E. coli* and unlocking oxidative activity against glucose.^3,4^ We first subjected M-RQW to directed evolution (libraries constructed by error-prone PCR) for thermostability and activity, generating the L3F3 variant. In a parallel approach, the PROSS 3 variant was designed from M-RQW by PROSS computational mutagenesis. PROSS 3 was used as the template for SDR of the L3F3 mutations, yielding the EvoPROSS variant that harbored 28 mutations from PROSS 3 together with N12D and Y576C from L3F3 (**S567P was introduced during the PCR amplification). The GOase wt was used as the query sequence for ancestral reconstruction, producing several ancestral enzymes of which node 45 was selected. Mutations from EvoPROSS were recombined into node 45 to design the final construct SarLacc, which has 159 substitutions relative to the original GOase wt from *Fusarium graminearum*. TTI at 70 °C (in-fold) relative to M-RQW; TAI_GAL_ (in-fold) relative to M-RQW using galactose as a substrate; and TAI_GLU_ (in-fold) relative to M-RQW using glucose as a substrate. The TTI and TAI were measured on *E. coli* lysates, and the measurements were obtained in quintuplet from cell-free extracts of independent cultures grown in 96-well plates using an ABTS-HRP coupled assay. MYA, million years ago. Full details about mutations and sequences can be found in the Supporting Information.

Initially, the parental M-RQW variant was subjected to a rapid directed evolution campaign, in conjunction with atomistic design calculations, in order to improve its thermostability and activity against glucose and galactose. We established the thermostability improvement (TTI) of mutants as the ratio between the initial and residual activity upon heating to 70 °C while the total activity improvement (TAI) was defined as the product of activity and expression (estimated from *E. coli* lysates). A dual high-throughput screening platform was prepared, such that mutant libraries were explored by measuring the H_2_O_2_ released by GOase using a colorimetric assay based on ABTS oxidation by horseradish peroxidase (CV ∼ 14%; Scheme S1). After two rounds of random mutagenesis and screening, we selected the L3F3 mutant that carried four new mutations (N12D, S102L, N413D, and Y576C) responsible for improving thermostability at 70 °C 2-fold, as well as enhancing activity 1.8- and 1.5-fold on galactose and glucose, respectively (Figures 1 and S1).

In a parallel approach, we subjected M-RQW to PROSS (a computational algorithm that leverages Rosetta atomistic design calculations and phylogenetic information) with a view to promote enzyme stability and expression by focusing on one-shot mutagenesis outside the active site.^19,20^ The algorithm yielded nine potential designs with an increasing number of mutations, producing 13 to 100 amino acid changes, of which the more conservative variants (PROSS 1, 13 mutations; PROSS 3, 28 mutations; and PROSS 4, 39 mutations) were cloned and expressed in *E. coli* and benchmarked for thermostability and activity (Table S1). PROSS 3 showed a TTI at 70 °C of 3.5-fold, while maintaining similar activities for galactose and glucose as the parental M-RQW (Figure 1). Thereafter, we constructed a new mutant library by site-directed recombination (SDR) in order to assess the combinatorial effect of the novel mutations in L3F3 obtained by directed evolution in the PROSS 3 sequence. The SDR approach mixes protein blocks containing 50% of the targeted positions with parental residues and 50% with the specific amino acid changes as a means to interrogate whether recombining the mutations in a new context is beneficial or not (Figure S2).^21^ The outcome of this experiment was the EvoPROSS mutant which incorporated the N12D and Y576C mutations (Figures 1S1, and S3 and Table S2). Carrying a total of 31 mutations, EvoPROSS exhibited a good balance between activity and thermostability, as it retained the thermostability of PROSS 3 but increased its activity by 30% relative to M-RQW (Figure 1).

### Ancestral Sequence Reconstruction

**Figure 2:**
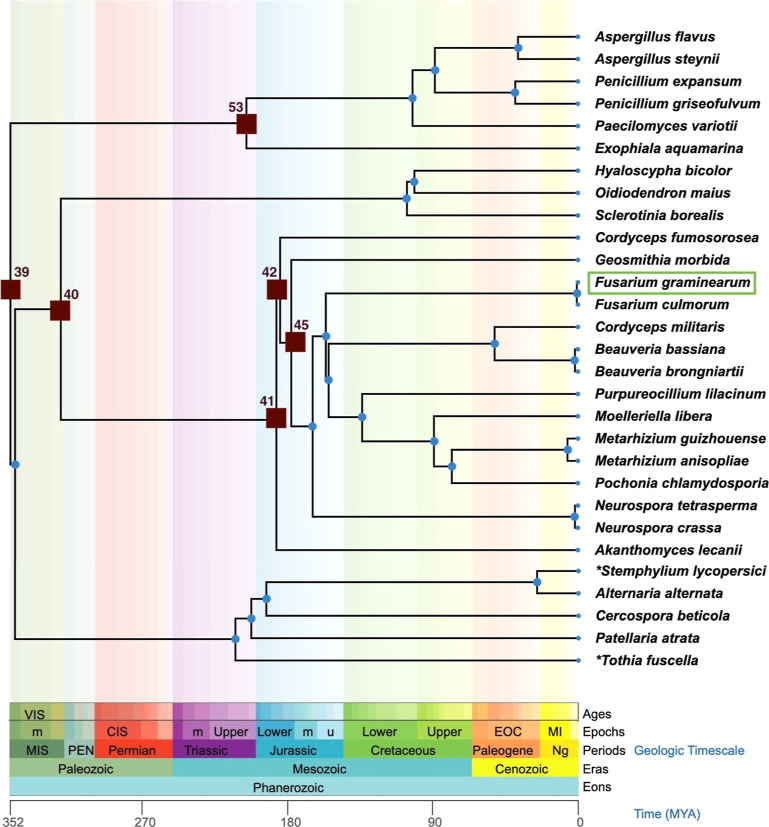
Phylogenetic tree built from 29 different GOase sequences, retrieved from a variety of subclasses in the Ascomycota division. The nodes whose sequences were selected for cloning and expression are depicted as brown squares. The tree was designed using TimeTree of Life (available at http://www.timetree.org/), which was employed to theoretically locate the ancestral nodes over a geological time scale. The colors used in the phylogenetic tree and TimeTree are conserved. GOase wt from *F. graminearum* was used as the query sequence for ASR. *Thyrospora lycopersici and *Microthyrium fuscellum* were reclassified as *Stemphylium lycopersici* and *Tothia fuscella*, respectively.

Ancestral sequence reconstruction (ASR) is a valuable tool when designing enzymes to improve their thermostability, expression, and promiscuity and hence we included it in our protein engineering workflow.^18,22,23^ We inferred six ancestral GOase nodes phylogenetically (nodes 39, 40, 41, 42, 45, and 53), from the oldest (node 39, c.a. 350 MYA) to the most recent nodes (nodes 42 and 45, c.a. 170 MYA) (Figure 2).

Interestingly, the six nodes identified carried between 119 and 186 mutations relative to GOase wt, including two insertions between positions 8/9 and 293/294 that led to proteins with two residues (641 amino acids) more than GOase wt (639 amino acids) (Table S3). It is worth noting that the GOase wt sequence was used as the query in our ASR experiment as opposed to the evolved M-RQW, such that neither carried the mutational backbone conferring activity on glucose nor enhanced expression. This was deliberate as we wanted to know if ASR rooted on the native GOase sequence could produce functional proteins with similar levels of expression as those obtained after directed evolution focused on expression.^3^ Pleasingly, all of the ancestral nodes were expressed functionally in *E. coli* as stable enzymes that acted on galactose but not on glucose (like the query sequence). We selected node 45 as a candidate for further engineering given its 5.6-fold improved thermostability at 70 °C and a striking ∼42-fold improvement in activity on galactose relative to M-RQW (measured from cell-free extracts, Figure 1). Sharing 76% sequence identity with M-RQW, node 45 carries 151 ancestral mutations that allow it to fold correctly and permitted functional heterologous expression in *E. coli* (Figure S3, Table S2). It is also worth noting that two of the 5 mutations (M70V and G195E) reported in the foundational directed evolution work to foster heterologous GOase expression in bacteria^3^ are indeed ancestral mutations. Although node 45 underwent a substantial change in amino acid composition, it still retained the overall arrangement of the native active site, including the highly conserved copper coordination sphere represented by the tyrosylcysteine complex at C228/Y272/Y495/H496/H581.

### Bringing ASR and Laboratory Evolution Together

When the M-RQW active pocket was reshaped to perform the regioselective oxidation of glucose at C6-OH, it was at the cost of reducing drastically its activity against d-galactose by ∼1000-fold.^4^ In our study, the ancestral node 45 was built from the query sequence of GOase wt, which allowed the enzyme to show noticeable activity for galactose (1/3 of that from GOase wt), strong thermostability, and high expression levels (see biochemical characterization section below); yet, node 45 lacked the activity against glucose presented by both M-RQW and EvoPROSS. We envisioned a final step to include the mutational backbone from the entire engineering campaign into node 45, with the goal of generating a hybrid variant that combines the enhancements produced by ASR and laboratory evolution. Accordingly, ancestral node 45 was used as a template, into which a total of 24 mutations from the EvoPROSS variant were inserted. Of this set of mutations, six came from the original GOase directed evolution campaign aimed at achieving heterologous expression and unlocking new-to-nature activity (i.e., the M-RQW mutations S10P, W290F, R330K, Q406T, V494A, and N535D), while 15 of the 28 mutations were from one-shot computational mutagenesis by PROSS. The reason why the remaining 13 mutations in PROSS 3 were not included in the final mutant was that they were already present in ancestral node 45. Such redundancy is consistent with the nature of the PROSS and ASR computational protein engineering methods, which both leverage phylogenetic calculations. The set of mutations was completed by adding the 2 stabilizing mutations selected from the directed evolution and SDR campaign (N12D, and Y576C) (Figure S3, Table S2). The final variant, termed SarLacc, harbored a total of 159 mutations relative to GOase wt (Table S4), producing an ∼4.2-fold improvement in thermostability at 70 °C. This mutant can act on glucose showing higher activity than parental M-RQW (see biochemical characterization below) but at the cost of jeopardizing its activity for galactose due to the inclusion of the mutational backbone for glucose activity (Figure 1).

### Large-Scale Production in *Pichia pastoris* and Biochemical Characterization

To benchmark the different variants, we transferred them from *E. coli* to *P. pastoris* (recently reclassified as *Komagataella phaffii*). *P. pastoris* can reach high cell densities in simple media of up to 130 g L^–1^ of dry cell weight, favoring upscale production of enzymes in a fed-batch bioreactor. Moreover, it can readily perform appropriate post-translational modifications and it streamlines any downstream processing due to its ability to secrete heterologous proteins into the culture broth.^24^ Accordingly, M-RQW, EvoPROSS, ancestral node 45, and SarLacc were cloned and overproduced in this yeast. The expression of M-RQW in *E. coli* is roughly 10 mg/L but when we cloned this variant in *P. pastoris*, its yield increased strikingly up to ∼160 mg/L in shaking flask production, c.a. 16-fold higher than its expression in bacteria, whereas the in-flask production of EvoPROSS and ancestral node 45 was 262 and 41 mg/L, respectively. As such, ASR achieved notable levels of heterologous GOase secretion without the need for a directed evolution campaign targeting expression.^3^ Similarly, PROSS increased expression ∼1.7-fold through a single shot of computational mutagenesis. When compared with node 45, the final SarLacc mutant boosted expression to 173 mg/L, a clear consequence of introducing the EvoPROSS mutations into the ancestral GOase scaffold (Table 1).

**Table 1:** Biochemical Characteristics of the Purified Variants Expressed by *P. pastoris*

variant	M-RQW^d^	EvoPROSS	node 45	SarLacc
mass (Da)^a^	69,244	69,909	75,699	76,595
mass (Da)^b^	68,448	68,609	68,284	68,391
glycosylation (%)^c^	1.15	1.86	9.80	10.71
expression level (mg/L in flask)	160 ± 22	262 ± 26	41 ± 3	173 ± 13
thermostability (*T*_50_ °C)	44.0 ± 0.3	52.0 ± 0.1	71.5 ± 2.5	57.0 ± 2.2
pH stability	5.0–9.0	5.0–9.0	5.0–9.0	5.0–9.0
initial turnover rates for d-galactose (μmol product μmol enzyme^–1^ min^–1^)	145 ± 3	75 ± 2	4080 ± 69	76 ± 3
initial turnover rates for d-galactose (μmol product μmol enzyme^–1^ min^–1^)	39 ± 1	75 ± 3	1.4 ± 0.04	78 ± 4

a Estimated by MALDI-TOF mass spectrometry.

b Computed with the Expasy ProtParam tool (https://web.expasy.org/protparam/).

c Calculated from the mass difference estimated by MALDI-TOF and that computed with the Expasy ProtParam tool.

d M-RQW is 1000 times less active toward d-galactose than GOase wt.^4^

To determine whether the *P. pastoris* variants could be useful for future production upscaling, we ran a large fermentation of M-RQW in a 10 L fed-batch bioreactor. Without any optimization of the fermentation parameters, 0.8 g/L production was achieved, placing our *P. pastoris* recombinant GOases in a good position for future industrial purposes (Figure S4). Subsequently, the *P. pastoris* variants were purified to homogeneity by IMAC (Figure S5) and their main biochemical properties were assessed (Table 1).

In terms of glycosylation, the M-RQW and EvoPROSS variants expressed by *P. pastoris* showed a similar degree of glycosylation to that from the original fungus, yet glycosylation of ancestral node 45 and SarLacc was enhanced to ∼10%. It is highly likely that new glycosylation sites have been generated among the common ancestral mutations present in these two mutants. Indeed, the GlycoEP server predicted three new *N*-glycosylation sites in the ASR and SarLacc variants (Asn36, Asn55, and Asn344; https://webs.iiitd.edu.in/raghava/glycoep/index.html). All in all, the degree of glycosylation remained low, as expected for recombinant proteins expressed in *P. pastoris*, which may facilitate future protein crystallization studies.

Kinetic thermostability was determined by measuring the *T*_50_ values, defined as the temperature at which the enzyme retains 50% of its activity after a 10 min incubation (Table 1, Figure S6). The *T*_50_ values were node 45 > SarLacc > EvoPROSS, given the 27, 13, and 8 °C increase in thermostability relative to M-RQW, respectively. The pH-dependent stability over the course of 10 days was measured, with all of the enzymes being stable in the pH range from 5.0 to 9.0 (Figure S7). Initial turnover rates for galactose and glucose were measured and as seen during the screenings, the ancestral node had the highest activity on galactose of the entire enzyme panel (55 U/mg), yet it lacked activity against glucose. By contrast, both the EvoPROSS and SarLacc mutants had similar turnover rates for galactose and glucose, the latter 2-fold higher than that of the parental M-RQW (Table 1).

**Figure 3:**
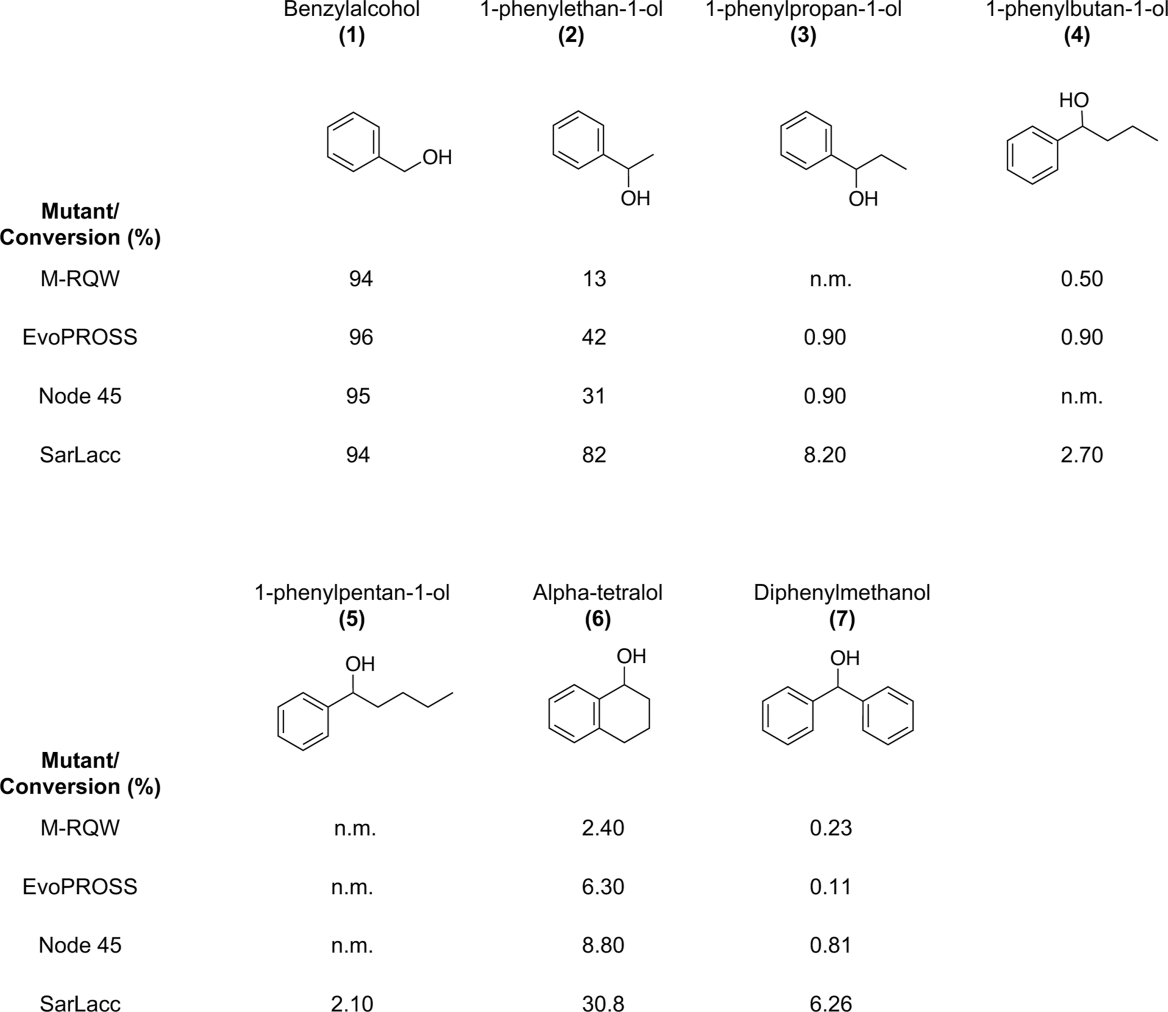
Conversion of different alcohols to their corresponding aldehydes/ketones. Reactions were performed in duplicate over 24 h at 35 °C and 750 rpm, in a final reaction volume of 200 μL containing 0.1 mg/mL of the purified enzyme, 5 mM substrates, 440 U catalase, 25 μg/mL HRP, 5% DMSO, and 0.5 mM CuSO_4_ in 100 mM NaPi buffer pH 7.0. The reactions were stopped by adding 200 μL of pure methanol and the products were analyzed by HPLC-MS. n.m., not measurable.

When M-RQW was engineered to unlock its activity on glucose, it also showed unexpected latent activities for secondary alcohols that has been the subject of study for years.^5,6^ Accordingly, we benchmarked our ensemble of variants against a representative set of primary and secondary aromatic alcohols with different degrees of bulkiness. Reactions with benzyl alcohol (**1**), 1-phenylethanol (**2**), 1-phenylpropanol (**3**), 1-phenylbutanol (**4**), 1-phenylpentanol (**5**), alpha-tetralol (**6**), and diphenylmethanol (**7**) were carried out and analyzed by HPLC-MS (Figure 3, Table S5). Regardless of the compound tested, the best conversions were produced by SarLacc. In good agreement with previous M-RQW studies,^4−6^ reactions with primary alcohol (**1**) achieved up to 94–96% conversion to benzaldehyde (Figure 3, Figure S8). There was greater or lesser latent activity of the mutants when assayed with secondary alcohols containing different alkyl chains (**2**) to (**5**). Parental M-RQW converted 13% of (**2**) into the corresponding ketone, with SarLacc achieving conversions up to 82% under the same reaction conditions (Figures 3 and S9).

Conversion became weaker as the length of the alkyl chain increased from (**3**) through to (**5**), yet SarLacc did show latent activity with conversions ranging from 8 to 2% (Figures 3 and S10–S12). Although cyclization of the alkane substitution into alpha-tetralol (**6**) produced a bulkier substrate, all four GOase variants were more active on this compound than on its aliphatic counterpart (**5**), with conversions up to 31% for the SarLacc mutant (Figure 3). With the bulkiest substrate (**7**), the mutants showed a similar trend as with (**6**) but offering much lower conversion rates (Figures 3, S13, and S14). In summary, our results indicate the substrate-binding plasticity of the GOase variants in terms of accommodation of bulky aromatic alcohols, although this plasticity does not extend to that of long aliphatic side chains.

### Mutational Analysis of GOase Variants

**Figure 4:**
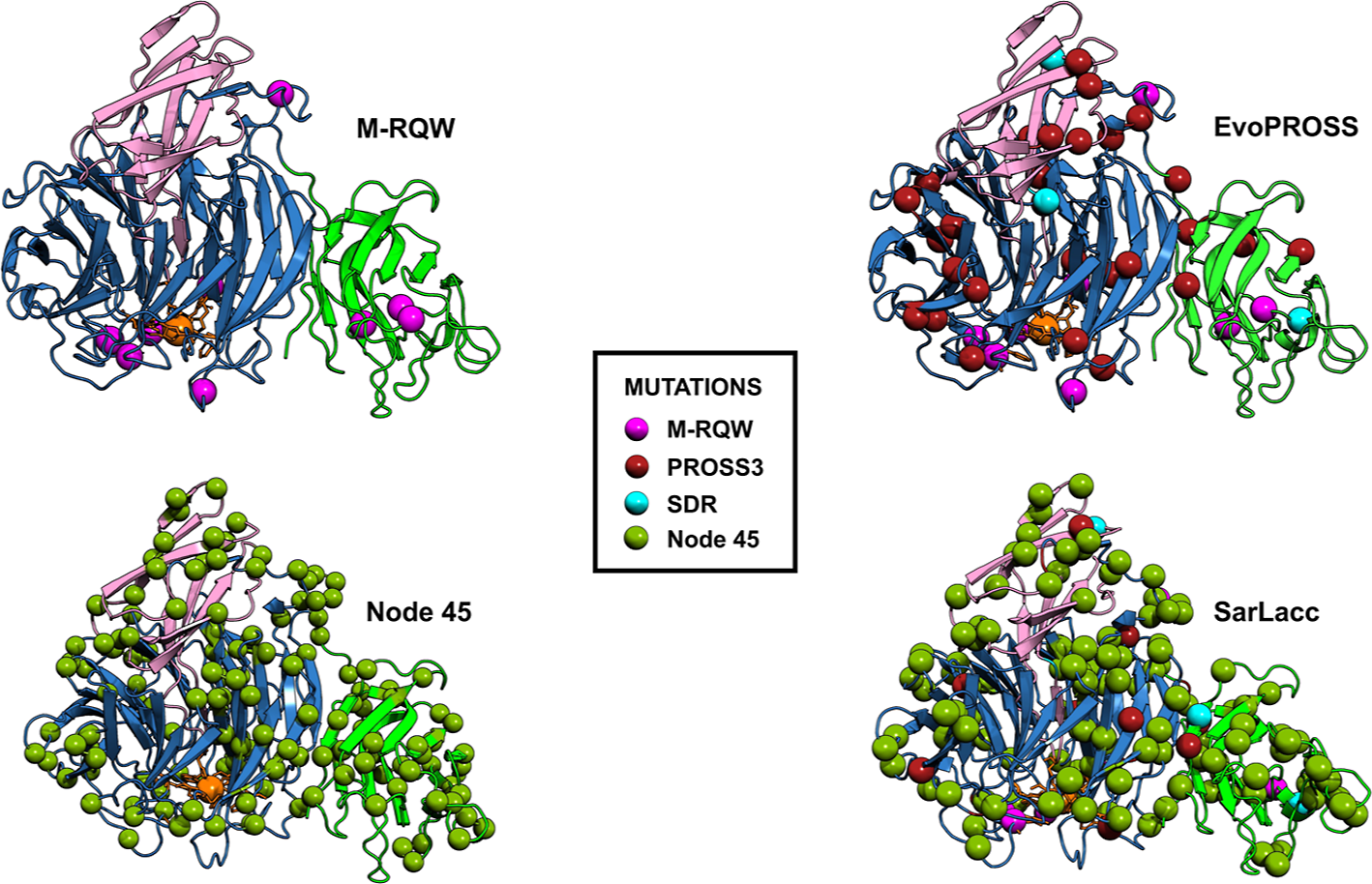
Overview of the mutations incorporated into the engineered versions of GOase. The structural models were created with Expasy Swiss model and crosschecked with AlphaFold.^27,28^ The GOase structures are represented in cartoon mode with domain I depicted in green, domain II in blue, and domain III in pink. The colored spheres indicate the position of the mutations in the GOase structure and the engineering campaign in which they were found. Orange spheres represent the copper ion in the active site.

At the structural level, GOase belongs to the group of β-folded proteins, and it is composed of three domains. The domain I (residues 1–155) adopts a β-sandwich structure, also known as the carbohydrate binding domain (CBD), which is folded by two β-sheets disposed one over the other one, each of them being formed of four β-strands connected in antiparallel orientation. The domain I plays a crucial role not only in facilitating substrate recognition but also in ensuring the proper folding of domain II, as its removal renders a nonfunctional enzyme. The domain II (residues 156–532) features a Kelch-like structural motif folded as a 7-fold β-propeller arrangement and attaches the cupric ion by three (Y495, H496, and Y272) of the four coordinating ligands. Finally, the domain III (residues 533–639) is characterized by an immunoglobulin-like (Ig) module, which carries the fourth coordinating ligand (H581) in an antiparallel β-ribbon that pierces domain II for a characteristic copper coordination.^25^ With over 25% of substitutions relative to GOase wt in the entire protein, the mutations in SarLacc are distributed across the three domains so that any epistatic effect between the mutations is complicated to interpret (Table S4). We can only speculate that the biochemical properties of our mutants might be modulated by the structural packaging of the individual domains, perhaps improving the interactions between each of them to different extents, influencing expression, stability, and reactivity (Figure 4).

For instance, of the 28 mutations from the PROSS campaign, only 6 mutations were located in β-strands, while the remaining substitutions were situated in disordered loop regions (Figure S15). The mutations that reside in these loop areas could potentially restructure these disordered fragments, facilitating the overall folding of the enzyme by enhancing the conformational flexibility. This effect could explain the strong expression of EvoPROSS in yeast, reaching secretion levels as high as 262 mg/L in the flask fermentation. By contrast, 37 of the 151 ancestral mutations in node 45 are located in β-strands (Figure S15), which might be responsible for the strong increase in thermostability (27 °C in *T*_50_) in our study. Mutations in β-strands could have a dual effect on thermostability by (i) reinforcing the intramolecular hydrophobic interactions of the β-strand and (ii) enhancing intermolecular packaging of close β-strands. Indeed, the number of hydrophobic residues in the GOase variants increased from 309 in M-RQW, to 315 in EvoPROSS, 322 in node 45, and 326 in SarLacc. The major effect of increasing the hydrophobic amino acid content was attributed to the addition of Pro residues from 41 in M-RQW to 51 in SarLacc, lowering the protein backbone entropy by restricting the conformations of neighboring residues.^26^

## Conclusions

The stereoselectivity and promiscuity of the M-RQW GOase variant reflects its strong potential in green chemistry, as demonstrated in previous studies assessing the kinetic resolution of benzylic and alkylic secondary alcohols to the transformation of renewable chemicals.^5,6,30^ M-RQW is a highly versatile enzyme that can be immobilized onto biosensors in order to monitor glucose or galactose. This variant has the advantage that it can be switched on/off given that its active state involves a tyrosyl radical that can be fully oxidized for functionality or that can be turned off upon reduction.^29^ For all these applications, thermostable GOases with endurance for longer operations and more promiscuous activities are needed that can be produced in industrially relevant hosts. Here, we engineered strongly secreted and thermostable GOases variants with latent activities by bringing together directed evolution with PROSS computational mutagenesis and ASR. Stabilizing mutations from the directed evolution campaign on M-RQW were recombined into the most stable PROSS design, and in parallel, ASR was performed to generate functionally expressed ancestral nodes carrying over 100 ancestral mutations. The final convergence of directed evolution, PROSS, and ASR allowed us to design hybrid GOases, the mutations of which seem to influence structural packaging of the different protein domains.

From a general perspective, directed enzyme evolution is, beyond a doubt, the most successful approach for protein engineering known to date. We are witnessing the advent of computational methods led by artificial intelligence, whereby the construction and exploration of mutant libraries is becoming more and more efficient aimed at achieving the “holy grail” of enzyme engineers, that is, the function global fitness optima.^31^ Our study sought to enhance the sequence and functional diversity of GOase by incorporating in the directed evolution workflow PROSS mutagenesis and ASR. With this strategy, we generated GOase designs that are readily expressed by *P. pastoris* on a g/L scale in a fed-batch bioreactor, showing an ensemble of biochemical properties, with good stability at high temperatures and across a range of pHs, with latent activities, all features that make them useful tools for organic synthesis and biosensing applications. Indeed, our results agree well with previous studies, highlighting the significance of PROSS and ASR to produce suitable candidates with which to start a directed evolution campaign or to be added to the workflow of a lab evolution experiment.^10−20,22,23^

## Supporting Information Available

Methods and protocols including directed evolution strategies, high-throughput screening assay, PROSS and ASR design, cloning and production in *P. pastoris*, protein purification, scale-up fermentation, kinetic thermostability assay, and analytical methods; GOase protein and DNA sequences; multiple sequence alignments; monitoring of the fed-batch bioreactor; SDS-PAGE gels; kinetic thermostabilities; pH-dependent stabilities; and HPLC-MS analysis (PDF)
