# Supplementary material for: Stable and Promiscuous Galactose Oxidases Engineered by Directed Evolution, Atomistic Design, and Ancestral Sequence Reconstruction
Source: ACS Synth Biol. 2024 Dec 13;14(1):239–46. doi: 10.1021/acssynbio.4c00653 (PMC11925331; doi:10.1021/acssynbio.4c00653)
Supplement: Supplementary file 1 — sb4c00653_si_001.pdf [file sb4c00653_si_001.pdf]

Supplementary Information for:

Stable and Promiscuous Galactose Oxidases Engineered by Directed Evolution, Atomistic Design and Ancestral sequence Reconstruction

Merve Keser,[†] Ivan Mateljak,[‡] Roman Kittl,[§] Roland Ludwig,[‖] Valeria A. Risso,[⊥] Jose Manuel Sanchez-Ruiz,[⊥] David Gonzalez-Perez,[†*] and Miguel Alcalde[†*]

[†] Department of Biocatalysis, Institute of Catalysis, ICP-CSIC, 28049 Madrid, Spain

[‡] EvoEnzyme S.L., Parque Científico de Madrid, 28049 Madrid, Spain

[§] DirectSens GmbH, Am Rosenbühel 38, 3400 Klosterneuburg, Austria

[‖] Department of Food Science and Technology, Institute of Food Technology, University of Natural Resources and Life Sciences, Muthgasse 18, 1190, Vienna, Austria

[⊥] Departamento de Química Física, Facultad de Ciencias, Unidad de Excelencia de Química Aplicada a Biomedicina y Medioambiente (UEQ), Universidad de Granada, 18071 Granada, Spain

* Corresponding authors' e-mail: malcalde@icp.csic.es, david.gonzalez.perez@csic.es.

Table of Contents

Materials 4
Reagents, molecular biology kits, strains and enzymes 4
Culture media 4
Methods 5
Directed evolution: first generation 5
Directed evolution: second generation 6
Site-Directed Recombination (SDR) 6
High-Throughput Screening (HTS) assay 8
PROSS 10
Ancestral Sequence Reconstruction (ASR) 11
Designing of SarLacc variant 12
Cloning and production in *P. pastoris* 13
Protein purification 14
Scale-up fermentation of GOase 14
Kinetic thermostability assay (T_{50} assay) 15
pH-Stability profile 16
Substrate panel using HPLC 16
Supporting references 17
Supporting schemes, figures, tables and GOase sequences 19
Scheme S1 19
Figure S1 20
Figure S2 21
Figure S3 23
Figure S4 24
Figure S5 25
Figure S6 26
Figure S7 27
Figure S8 28
Figure S9 29
Figure S10 30
Figure S11 31
Figure S12 32
Figure S13 33

Figure S14 34
Figure S15 35
Table S1 36
Table S2 37
Table S3 40
Table S4 43
Table S5 44
Table S6 46
Table S7 48
GOase sequences in this study 49

Materials

Reagents, molecular biology kits, strains and enzymes

All chemical reagents were of the highest purity commercially available. *Taq* polymerase was purchased from Thermofisher Scientific (Massachusetts, USA). The Gene Morph II kit was acquired from Agilent Technologies (Santa Clara, CA, USA). Restriction enzymes, Gibson Assembly kit, and Q5 High-Fidelity PCR kit were obtained from NEB (New England Biolabs, Massachusetts, USA). iProof High-fidelity polymerase was from BioRad (Hercules, CA, USA). DNA extraction and PCR clean-up kit utilized was from Macherey-Nagel (Düren, Germany). Synthetic DNA constructs and primers were purchased from Twist Biosciences (San Francisco, CA, USA) and IDT (Integrated DNA Technologies, Leuven, The Netherlands). The pPICZ-α vector was acquired from Invitrogen (Waltham, Massachusettes, USA). Strains of *E. coli* XL1-Blue and electrocompetent BL21(DE3) were from Agilent Technologies (Santa Clara, CA, USA) and LGC Biosearch Technologies (Teddington, Middlesex, UK), respectively. The *Pichia pastoris* PPS9011 Mut^S strain was kindly provided by DirectSens GmbH (Vienna, Austria). The plasmid pGAO-(M-RQW) containing glycerol stock was kindly donated by Prof. Frances Arnold Lab (Caltech, CA, USA).

Culture media

Auto-induction media supplemented with carbenicillin (AIM/C) was composed of the following reagents (per liter of media): 12 g bacto tryptone, 24 g yeast extract, 3.3 g $(NH_4)_2 SO_4$, 6.8 g KH_2PO_4, 7.1 g Na_2HPO_4, 0.5 g glucose, 2.0 g lactose, 0.15 g $MgSO_4$, and 1 mL of 100 mg/mL carbenicillin.

Yeast extract peptone dextrose media (YPD) supplemented with zeocin (Invitrogen, Massachusetts, USA) for *P. pastoris* proliferation was composed of the following (per liter of media): 20 g yeast extract, 10 g peptone from casein, 4.4 g glucose monohydrate, 15 g bacto agar (for agar plates), 1 mL of zeocin at 100 mg/mL.

BMM2/30 media for a selection of best-producing variants expressed by *P. pastoris* in 96 deep-well plates contained the following (per 0.5 L of media): 100 mM potassium phosphate (KPi) buffer pH 6.0, yeast nitrogen base (YNB) without amino acids solution containing ammonium sulphate (YNB, 0.034 g/mL), 0.2 mg/mL biotin, and 3% methanol.

BMMY media for *P. pastoris* fermentation was composed of the following (per liter of media): 20 g peptone from soy, 10 g yeast extract, 100 mM KPi buffer pH 6.0, YNB solution without amino acids containing ammonium sulphate (0.034 g/mL) plus extra ammonium sulphate (0.1 g/L), 0.4 mg/mL biotin, and 3% methanol.

Methods

Directed evolution: first generation

The 1st generation was run on the M-RQW gene by using *Taq* DNA polymerase and varying concentrations of $MnCl_2$ to optimize mutational loads per GOase gene. Reactions were prepared in a final volume of 50 µL containing DNA template (0.2 ng/µL), 100 µM forward and reverse primers (see **Table S6**), 0.3 mM dNTPs (0.075 mM each), 1.5 mM $MnCl_2$, 3% (v/v) DMSO, 1x *Taq* polymerase buffer, and 0.05 U/µL *Taq* polymerase. The PCR was run in a BioRad T100 Thermocycler (Hercules, CA, USA) with the following parameters: initial denaturing at 95°C for 2 min (1 cycle), followed by 28 cycles of 94°C for 60s, 60°C for 90s, and 74°C for 2 min. The final extension was carried out for one cycle at 74°C for 10 minutes, then cooling and storage at 4°C. The PCR products were electrophoresed on a 1% agarose gel and purified using the Macherey-Nagel PCR clean-up kit (Düren, Germany). The purified PCR products were then ligated to the pTrcHis2B linearized plasmid with the help of engineered overlapping ends to the vector through Gibson assembly following the manufacturer´s specifications (NEB, Massachusetts, USA). The assembled plasmids were transformed into BL21(DE3) electrocompetent *E. coli* cells (Electroporator Eppendorf 2510, Germany) and plated on Luria-Bertani supplemented with 50

μg/mL carbenicillin (LB/C) plates. Nine hundred individual clones were propagated and screened for thermostability following the high-throughput screening mentioned below.

Directed evolution: second generation

The 2nd generation was performed by error-prone PCR using the GeneMorph II Random mutagenesis kit to ensure a well-distributed mutational landscape, as well as reduction of mutational bias. Mutagenesis was carried out according to the manufacturer's instructions with minor adjustments to yield a low mutational frequency amounting for 1-4.5 mutations per GOase gene. Reactions were prepared in a total volume of 50 μL containing the following components: DNA template (3,000 ng), 300 nM forward and reverse primer (see **Table S6**), 0.8 mM dNTPs (0.2 mM each), 2.5 U/μL Mutazyme II DNA polymerase, and 1x polymerase buffer. The PCR was run in a BioRad T100 Thermocycler (Hercules, CA, USA) and the parameters used were the same as described for the first generation. The PCR products were treated the same as the first generation such that they were placed on 1% agarose, extracted, and purified using the Macherey-Nagel PCR clean-up kit (Düren, Germany). The overlapping regions in the primers used (shown in capitals in **Table S6**) were compatible with the pTrcHis2B linearized plasmid for facilitating ligation when using Gibson assembly (NEB, Massachusetts, USA). Following ligation, the plasmids were transformed into *E. coli* BL21(DE3) electrocompetent cells. Eight hundred individual clones were propagated and screened in a high-throughput fashion for thermostability/activity improvement as mentioned below.

Site-Directed Recombination (SDR)

PROSS 3 design was the template to construct all the PCR fragments needed for SDR (**Figure S2**). In total, four fragments were generated for Gibson assembly; however, due to the proximity of mutations found at positions 12 and 102, we had to amplify an extra intermediary fragment, termed Fragment 0, which was the template of PCR Fragment 1 to extend the overlapping areas for efficient assembly and cloning of GOase gene. Therefore, five fragments were synthesized by PCR and designed with overlapping ends each other and to the linearized

vector for Gibson assembly. PCR reactions were prepared in a total volume of 50 µL containing the following components: DNA template (PROSS 3, 10 ng), 5 µM forward and reverse primers necessary for each respective fragment (**Table S6**), 3 mM DMSO, 0.8 mM dNTPs (0.2 mM each), 0.02 U/µL iProof HF polymerase, and 1x iProof HF buffer. Primers for Fragment 0 were SDR-12Asn_Fw, SDR-12Asp_fw, SDR-102Ser_fw, and SDR_102Leu-Rv. Fragment 1 primers were SDR_PCR2 _Fw and SDR_PCR2_Rv (a combination of Fragment 0 and 1 was PCR1 in **Figure S2**). Primers used for Fragment 2 were SDR_102Leu_Fw, SDR_102Ser_Rv, SDR_413Asn_Rv, SDR_413Asp_Rv (PCR2 in **Figure S2**). Primers used in Fragment 3 were SDR_413Asp_Fw, SDR_413Asn_Fw, SDR_576Try_Rv, SDR_576Cys_Rv (PCR3 in **Figure S2**). Primers used in Fragment 4 (PCR4 in **Figure S2**) were SDR_576Cys_Fw, SDR_576Tyr_Fw, SDR_FinalPCR_Rv (**Table S6**). Reactions were performed by using the BioRad T100 Thermocycler (Hercules, CA, USA) with the following PCR parameters; for Fragment 0 and 1 they were: one cycle at 98°C for 30 seconds followed by 30 cycles of first 98°C for 10s, 61°C for 20s, and 72°C for 15s. The final extension at 72°C for 10 minutes was done for 1 cycle. PCR cycles for Fragment 2, 3, and 4 were: one cycle at 98°C for 30 seconds followed by 30 cycles of first 98°C for 10s, 62°C for 20s, and 72°C for 15s. Final extension at 72°C for 10 minutes was done for 1 cycle. PCR products were placed on 1% agarose gel and extracted using the Macherey-Nagel DNA extraction and PCR clean-up kit (Düren, Germany). All the fragments were assembled using Gibson assembly thanks to the overlapping ends that complemented each other along with the vector (**Figure S2**). Plasmid pTrcHis2B was used to build the full construct where the fragments were at least six times in picomoles to that of the vector concentration. The reaction was carried out at 50°C for 3 hours followed by the dilution of the reaction to 1:3 (v:v) before transforming into electrocompetent BL21(DE3) *E. coli* cells.

High-Throughput Screening (HTS) assay

Individual clones were picked onto sterile flat bottom 96-well plates (Sarstedt, Numbrecht, Germany) pre-filled with 100 µL of LB/C media. Column 6 was inoculated with the M-RQW variant as internal standard, and H1 well (negative control) with an empty plasmid colony. The plates were sealed with parafilm and incubated for propagation at 37 ºC overnight (pre-culture), 220 rpm and 80% humidity (Minitron Infors, Bottmingen, Switzerland). The pre-culture plates were replicated using a CR1000 96-pin replicator (Enzyscreen, Heemstede, The Netherlands) onto a sterile U-bottom 96-well plate (Corning, New York, USA) pre-filled with 200 µL of auto-induction media supplemented with 50 µg/mL carbenicillin (AIM/C) (expression plate). The expression plates were sealed with parafilm and incubated overnight at 30 ºC, 220 rpm and 80% humidity (Minitron Infors, Bottmingen, Switzerland). Then, the plates were centrifuged at 800 *xg* for 10 minutes at 4 ºC (Eppendorf 5810R, Hamburg, Germany), and the supernatant was discarded. The resulting pellets were frozen at -80 ºC overnight to initiate cell fragmentation. The frozen pellets were left to thaw the next day on the bench before adding to each well 200 µL of lysis buffer containing 0.5 mg/mL lysozyme (Thermofisher Scientific, Massachusetts, USA), 2 U DNAse I, 0.5 mM $CuSO_4$, and 100 mM sodium phosphate (NaPi) buffer pH 7.0. Next, the plates containing lysis buffer were incubated at 37 ºC for 1.5 hours and spun down at 2,000 *xg* for 30 minutes at 4 ºC (Eppendorf 5810R, Hamburg, Germany). At this point, 50 µL and 20 µL of cell-free extract (CFE) from each well were transferred to a 96-well PCR plate and a flat bottom 96-well plate, respectively, with the help of a robotic liquid handler (Freedom Evo TECAN 100, equipped with 96-multi channel arm (MCA), TECAN, Switzerland) to carry out the thermostability assay (TTI assay). The filled PCR plates with 50 µL of CFE were sealed with a thermoresistant seal (Thermofisher Scientific, Massachusetts, USA) and incubated at 70 ºC in a thermocycler BioRad T100 (Hercules, CA, USA) for 10 minutes, immediately followed by placing the plates on ice for 10 minutes and bringing them to room temperature on bench for 10 minutes before assessing the residual activity (RA). The RA was measured with both galactose and glucose by

transferring 20 µL aliquots to two flat bottom 96-well replica plates using Freedom Evo TECAN 100, followed by the addition of 180 µL of reaction buffer (100 mM galactose or 400 mM glucose, 7 U/mL HRP, 2 mM ABTS, 100 mM NaPi buffer pH 7.0 and 0.5 mM $K_3[Fe(CN)_6]$) with the help of a Multidrop Combi robot (Thermofisher, Massachusettes, USA). Enzymatic activity was monitored at room temperature using a NanoQuant Infinite M200 plate reader (TECAN, Switzerland) following the coupled reaction of GOase with horseradish peroxidase (HRP) at 418 nm (ε_{418} $ABTS^{\bullet+}$ = 36,000 M^{-1} cm^{-1}). In this reaction, HRP uses the H_2O_2 released by GOase as a co-substrate for ABTS oxidation (**Scheme 1**).[1] The initial activity (IA) was immediately assayed for glucose and galactose by transferring 20 µL aliquots of CFE into two flat bottom 96-well replica plates with the help of Freedom Evo TECAN 100 and adding 180 µL of reaction buffer to follow the coupled ABTS-HRP assay as mentioned above. The background signal of the H1 well (negative control) was subtracted from all the values and then normalized against the signal of the parental type in each respective plate. The total thermostability improvement (TTI) was based on the ratio of RA/IA > 1 (being 1 the normalized thermostability of the parent). False positives were ruled out by conducting three consecutive re-screenings as mentioned below.

- First re-screening: 10 µL aliquots of the best performance clones were removed from the initial screening master plates and inoculated in 90 µL of LB/C media in 96-well microtiter flat-bottom plates (Sarstedt, Numbrecht, Germany). Columns 1 and 12 (rows A and H) were left empty to prevent evaporation. The plates were incubated overnight at 37 ºC, 220 rpm and 80% humidity in a Minitron shaker (Infors, Bottmingen, Switzerland). Then 10 µL of pre-cultures were transferred to the four adjacent wells, which contained 90 µL of fresh LB/C media. These new cultures were further incubated overnight at 37 ºC, 220 rpm and 80% humidity in a Minitron shaker (Infors, Bottmingen, Switzerland). The following day, the plates were replicated using a sterilized cell replicator (EnzyScreen CR1000 96-pin replicator, Heemstede, The Netherlands) in a U-bottom 96-deepwell plates (Corning, NY, USA) pre-filled with 200 µL of LB/C media supplemented with 1mM IPTG to induce GOase expression; these plates were incubated overnight at 30 ºC, 220 rpm

and 80% humidity (Minitron shaker Infors, Bottmingen, Switzerland). After GOase expression phase, the plates were subjected to cell lysis, and the resulting CFE was used for the HTS thermostability assay described above. Accordingly, every single mutant was grown in 4 wells. Parental type was also subjected to the same procedure, but placed on lane E of the plates, wells 7–11.

- Second re-screening: An aliquot of the best clones from the first re-screening was inoculated in 10 mL of LB/C and incubated overnight at 37 °C and 250 rpm (Minitron shaker Infors, Bottmingen, Switzerland). Plasmids from these cultures were extracted with Macherey-Nagel plasmid miniprep kit and they were transformed again, along with the parental type, into electrocompetent BL21(DE3) *E. coli* cells. Five individual colonies of every single mutant were inoculated in 100 µL of LB/C into flat-bottom 96-well plates (Sarstedt, Numbrecht, Germany). Columns 1 and 12 (rows A and H) were left empty, and the plates were further incubated overnight at 37 °C and 250 rpm (Minitron shaker Infors, Bottmingen, Switzerland) to generate pre-cultures. Then, GOase expression was carried out as described in the first re-screening, and CFEs were subjected to the HTS assay for thermostability and activity as described above.

- Third re-screening: Fresh transformants of selected mutants -and parental type- were inoculated in 20 mL of AIM/C media and incubated 24 hours for expression at 30°C and 220 rpm (Minitron shaker Infors, Bottmingen, Switzerland). Then, the cultures were transferred to 15 mL falcon tubes and spun down at 2,000 *xg* for 15 minutes at 4 ºC (Eppendorf 5810R centrifuge, Hamburg, Germany). Next, the supernatant was discarded, and cell pellets were processed as described before to obtain CFE, except for using a scaled-up version of the protocol using 6 mL of lysis buffer. Next, the CFEs were subjected to TTI assay and activity measurements as described above.

PROSS

Protein **R**epair **O**ne-**S**top **S**hop algorithm was used on M-RQW as instructed by the PROSS website (https://pross.weizmann.ac.il/step/pross-terms). Residues at positions 229, 273,

290, 330, 407, 496, 497, and 582 -ensuring glucose activity and active site integrity- were ruled out for mutagenesis. PROSS output nine designs in total with increasing mutational loads along with their FASTA sequence and a PDB file for each design. Design 1 (PROSS 1), Design 3 (PROSS 3), and Design 4 (PROSS 4) with 13, 28, and 39 mutations, respectively, were synthesized (Twist Biosciences, San Francisco, CA, USA) containing overlapping ends to the linearized vector pTrcHis2B for cloning by Gibson assembly following manufacturer´s specifications (NEB, Massachusetts, USA). The constructed plasmids were transformed into XL1-Blue chemically competent *E. coli* cells followed by propagation for subsequent DNA gel extraction (Macherey-Nagel, Düren, Germany). DNA samples from each design were verified by sequencing and further transformed for expression into electrocompetent BL21(DE3) *E. coli* cells. PROSS designs were expressed in small flasks using AIM/C media and incubated at 30 ºC, 220 rpm for 24 hours (Minitron Infors, Bottmingen, Switzerland) and subjected to the thermostability assay (TTI assay) as described before.

Ancestral Sequence Reconstruction (ASR)

Multiple sequence alignment (MSA) was performed by retrieving all fungal galactose oxidase homologs using the BLAST (https://blast.ncbi.nlm.nih.gov/Blast.cgi) tool from the National Center for Biotechnology Information (NCBI) with a sequence number cut-off at 500 sequences. Sequences that were derived from insect or human genomes, as well as “putative”, “hypothetical”, or “predicted” were excluded from the selection for refinement amounting to a total of 50 sequences that were further processed using Molecular Evolutionary Genetics Analysis (MEGA-X) program.[2] The refinement of the MSA included the alignment itself and defining key structural features of GOase such as the presence of disulfide bridges, alignment of the active site residues, identification of the enzyme domains in the sequences, and removal of gaps, insertions, or deletions in the aligned sequences. To have a grounded tree, sialidase from *Micromonospora viridifaciens* (PDB ID: 1EUT) with a sequence identity of 30% was placed as the first and last position within the alignment as an out-group. The derivation of the ancestral node

tree was carried out Al-Hambra Cluster and the Bayesian method[3]. The nodes derived from the tree had posterior probabilities of 0.8 or higher with each node in FASTA format. Six nodes were chosen for heterologous expression (resurrection) by synthesizing the genes (Twist Biosciences, San Francisco, CA, USA) with overlapping ends compatible with the pTrcHis2B vector for Gibson assembly. The constructed plasmids were chemically transformed into XL1-Blue *E.coli* cells for plasmid DNA propagation followed by the extraction with Macherey-Nagel miniprep kit for sequence verification. Verified plasmids (Eurofins Genomics, KY, USA) were further transformed into electrocompetent BL21(DE3) *E. coli* cells for expression in 20 mL auto-induction media supplemented with 50 μg/mL carbenicillin and incubated for 24 hours at 30 ºC, 220 rpm (Minitron Infors, Bottmingen, Switzerland) and subjected to the thermostability assay (TTI assay) mentioned before. The resurrected node resulting in the highest thermostability improvement was chosen for further characterization; thus node 45. The bioinformatics analysis of the resurrected nodes was performed by using Clustal Omega[4] for sequence identities and FASTA format of resurrected nodes, which was received in conjunction with the constructed ancestral phylogenetic tree. The structure of node 45 was modelled using ExPASy Swiss Model crosschecked with AlphaFold.[5,6]

Designing of SarLacc variant

Using as a template the node 45 variant, 24 residues were substituted (S10P, W290F, R330K, Q406T, V494A, N535D, Q63H, S104M, A153P, A193Q, S206I, A269P, M278L, S311L, A323N, Y436W, V492P, I498V, N531P, Q552D, S563Q, N12D, S567P, Y576C, *see* details in **Figure S3** and **Table S2**) to obtain the final variant SarLacc. The integration of mutations resulted from variant EvoPROSS and M-RQW into node 45 scaffold whereby the gene was synthesized through Twist Biosciences (San Francisco, CA, USA) with overlapping ends compatible with the pTrcHis2B for Gibson assembly. The assembly was carried out at 50 ºC for 3 hours followed by the dilution of the reaction to 1:3 (v:v) before transformation into chemically competent XL1-Blue *E. coli* cells for plasmid DNA propagation. Next, plasmids were extracted with Macherey-Nagel

miniprep kit for sequence verification. The verified plasmid (Eurofins Genomics, KY, USA) was further transformed into electrocompetent BL21(DE3) *E. coli* cells for expression in 20 mL of auto-induction media supplemented with 50 µg/mL carbenicillin and incubated for 24 hours at 30 ºC and 220 rpm (Minitron Infors, Bottmingen, Switzerland) as described before. Then, the culture was centrifuged at 2,000 *xg* for 15 min and the supernatant was discarded. Cell pellets were lysed as described in the high-throughput screening section using 6 mL of lysis buffer and finally subjected to the thermostability assay (TTI assay) mentioned.

Cloning and production in *P. pastoris*

M-RQW, EvoPROSS, node 45, and SarLacc variants were PCR amplified with Q5 polymerase and cloned by Gibson assembly into vector backbone of pPICZ-α consisting of alpha mating factor (MFα) signal peptide in frame with a 6xHis-tag. Primers used for the amplification of the vector and the engineered variants can be found in **Table S6**. The His-tagged constructs were transformed into XL1-Blue *E.coli* for propagation and extracted using Macherey-Nagel miniprep kit for sequence verification (Düren, Germany). The variants were further linearized and transformed into *P. pastoris* strain PPS901(YPD)1 Mut^S by electroporation using 100 ng of linear DNA and plated on rich medium with increasing zeocin concentrations (100, 250, 500, 1000 µg/mL) for selection. Clones growing on the highest zeocin concentration plate were selected for expression in 96 deep-well plates using YPD media for 2 days at 30 ºC, 250 rpm, and 80% relative humidity (Minitron Infors, Bottmingen, Switzerland) sealed with a breathe-easy film, followed by the addition of BMM2/30 media (200 mM KPi buffer pH 6.0, 1:10 yeast nitrogen base (YNB), 0.2 mg/L biotin, and 3% methanol (v/v)). Enzymes were expressed for additional 3 days at 25 ºC, 250 rpm and 80% humidity (Minitron Infors, Bottmingen, Switzerland) followed by centrifugation at 4,000 x*g* (Eppendorf 5810R centrifuge, Germany). Twenty microliters of supernatants were transferred to a flat bottom 96-well plate with the help of a Freedom Evo TECAN 100 robot and submitted to the ABTS-HRP screening assay to determine IA as mentioned previously. Clones giving the best volumetric activity were selected as the main clones for further production. Then,

enzymes were expressed in 0.5 L cultures by using buffered minimal methanol media (BMMY) with the addition of 1:500 (v/v) dilution of J673 antifoam (Struktol) for 5 days at 25°C and 120 rpm (HT Multiron, Infors, Bottmingen, Switzerland). Cultures were centrifuged at 4,000 x*g* for 15 minutes (Eppendorf 5810R centrifuge, Germany), the pellet was discarded since GOase expression was directed to extracellular media, and the supernatant was processed for purification by IMAC.

Protein purification

GOase variants were purified to homogeneity by their affinity of the His-tag to Ni-NTA resin with His-Trap FF 5 mL columns using immobilized metal ion affinity chromatography (IMAC) and Äkta Pure system (Cytiva Massachusettes, USA). His column was equilibrated in Buffer A (50 mM KPi buffer pH 8.4 containing 300 mM NaCl) and then crude extracts were flowed through the columns for the binding phase. Next, unspecific proteins were washed out of the column with Buffer A containing 5 mM imidazole, and GOase variants were eluted with Buffer A containing 500 mM Imidazole. The concentration of the purified proteins was determined by Bradford assay (BioRad, Hercules, California, USA) adapted to 96-well plate format, and purity was further assessed by SDS-PAGE.

Scale-up fermentation of GOase

The production of GOase was adapted from the *Pichia* Fermentation Process Guidelines (Invitrogen, CA, USA) using an HT Minifors 2 Bioreactor system (Infors, Bottmingen, Switzerland). The inoculum (1 L) was cultivated at 30 ºC, 220 rpm for 20 hours (HT Multiron shaker, Bottmingen, Switzerland) and used to inoculate 10 L of fermentation medium containing 4% (w/v) glycerol as carbon source. The temperature during the fermentation process was maintained at 25 ºC and the pH was constantly adjusted to 6.0-7.0 with the addition of 25% NH_4OH. During the glycerol batch phase, the oxygen supply was directed using an airflow of 1.0 vvm and an agitation speed of 800 rpm. After the glycerol batch phase, a feed of 50% (w/v) and 13 mL/L PTM_1 plus 0.2 g/L biotin mix was initiated, and the feed rate was controlled to maintain oxygen level at 40%. After

28 hours; thus, two hours prior to the end of glycerol feed, methanol was added to a final concentration of 0.3% (v/v) to initiate induction of GOase expression. Protein expression was carried out using a feed of 100% methanol containing 12 mL/L PTM_1 plus 0.2g/L biotin mix with a feed rate regulated to maintain an oxygen level of 30%. Cultivation was stopped after 95 hours on methanol feed (128 hours total fermentation time) and the supernatant was harvested by centrifugation at 4,000 x*g* at 4 ºC for 30 minutes (Eppendorf Centrifuge 5920R, Hamburg, Germany). The supernatant was filtered using a 0.22 µm Steritop vacuum filtration system (Millipore, Massachusettes, USA) to remove residual particles, and rebuffered with *dd*H_2O for concentration using a tangential flow filtration system equipped with a 0.45 µm filter (Sartorius, Gottingen, Germany) before preparation for IMAC purification as mentioned above.

Kinetic thermostability assay (T_{50} assay)

GOase variants expressed in *P. pastoris* and purified to homogeneity were diluted with 10 mM NaPi buffer pH 7.0 to 0.1 mg/mL, except node 45, which was 0.007 mg/mL. Initial activity (IA) measurements were done per enzyme in eight replicates at room temperature using ABTS-HRP coupled assay consisting of 20 µL of purified enzymes and 180 µL of reaction buffer (100 mM galactose, 7 U/mL HRP, 2 mM ABTS, and 100 mM NaPi buffer pH 7.0). The steps in the temperature gradient for determining the T_{50} were from 45 to 72 ºC, except node 45, which was from 45 to 90 ºC. The purified enzymes were aliquoted in 50 µL triplicates in non-skirted 96-well PCR plates (Thermofisher Scientific, Massachusetts, USA). The plates were sealed with a thermoresistant adhesive (Thermofisher Scientific, Massachusetts, USA) and placed in the thermocycler (BioRad, Hercules, CA, USA) for 10 min, followed by 10 min on ice, and subsequently brought to room temperature for additional 10 min before activity measurement. Residual activities (RA) were assayed by transferring 20 µL aliquots from the 96-well PCR plate into a flat bottom 96-well plate using the Freedom Evo TECAN 100 (TECAN, Switzerland), followed by the addition 180 µL of reaction buffer using a Multidrop combi robot. Kinetic measurements were monitored at 418 nm in the NanoQuant Infinite M200 plate reader (TECAN,

Switzerland). The residual activities in the graphs were defined as the ratio RA/IA and expressed as percentages. The T_{50} value was defined as the temperature that reduces 50% of the initial activity of GOase after 10 minutes of heat treatment.

pH-Stability profile

Assessing the stability of the final variants at different pH values was carried out by incubating the enzymes in the following buffers: 50 mM citrate buffer for pH 2.0-5.0, 50 mM KPi buffer for pH 6.0-7.0, and 50 mM Tris-HCl buffer for pH 8.0-9.0. Per buffer composition, 0.2 mg/mL of M-RQW, 0.1 mg/mL EvoPROSS, 0.007 mg/mL node 45 and 0.2 mg/mL SarLacc were added to 96 deep-well plates and incubated at 4 ºC for 7 days. Aliquots of incubation mix were extracted at time zero, 0.5, 1, 2, 48, and 168 hours and monitored for activity by using the ABTS-HRP coupled assay. The specific activity of each enzyme was calculated over time and normalized against the activity of the enzyme measured at time zero.

Substrate panel using HPLC

Enzymatic reactions using purified variants (named R) were set up in the following conditions: 5 mM substrates, 25 µg/mL HRP, 0.1 mg/mL GOase variants, 440 U/mL catalase, 5% DMSO, and 0.5 mM $CuSO_4$ in 100 mM NaPi buffer pH 7.0. Control samples (named CTRL) were performed as described above but in the absence of GOase. Control and enzymatic reactions were performed in duplicates (R1, R2 and CTRL). The reaction volume was 200 µL and it was carried out in 1.5 mL tubes at 35 ºC and 750 rpm using an Eppendorf Thermomixer C (Hamburg, Germany). Reactions were stopped after 24 hours with the addition of 200 µL methanol. The precipitates were removed by centrifugation at 15,000 x*g* for 5 minutes (Eppendorf 5418 centrifuge, Germany). Next, ten microliters of the reactions were injected for reverse high-performance liquid chromatography (HPLC) (Shimadzu LC-2050C-3D, Kyoto, Japan) equipped with a VP-ODS C18 column (150 mm × 4.6 mm, 5 µm) thermostatized at 40 ºC, using a flow rate of 1 mL/min, and a photodiode array detector (PDA detector). The injections were performed by an autosampler at room temperature. Calculation of the product yields was performed as follows:

the control and the reaction chromatogram were integrated, and a table of the integrated peaks was generated using the Lab Solutions post-data analysis. The integrated peaks pertaining to each control and reaction sample were set as percentages using the total peak area of the respective chromatograms. The control integrated peaks were then subtracted from those of the reaction integrated giving the product yield formed as a percentage. The following equation was used for calculations:

Integral sample peak (%) - Integral peak control (%) = Product yield (%)

Reactions were repeated with SarLacc variant and they were injected to UHPLC coupled to a triple quadrupole MS (QQQ-EVOQ Elite) for identification of products using an Ace Excel 3, C18-AR (150 x 3.0 mm, 3 µm) column thermostatized at 40 ºC. The mobile phase consisted of A (Milli Q + 0.1% formic acid) and B (LC-MS grade acetonitrile). The flow rate was at 0.4 mL/min with a gradient of A and B starting at time zero with 80% A and 20% B sustained until minute 15 at 20% of A and 80% of B followed by 100% B at minute 16 to 18 coming back to initial ratios of 80% A with 20% B at minute 18.10 until 20 minutes. MS detection was at positive electrospray ionization (ESI+) mode using scan mode at a mass interval of 80-500. Aldehyde and ketone products were identified based on their retention time (t_R, represented in min), UV spectrum, molecular mass signals, and fragmented patterns compared with known aldehyde or ketone mass spectra from the literature.

Supporting references

(1) Rogers, M. S.; Tyler, E. M.; Akyumani, N.; Kurtis, C. R.; Spooner, R. K.; Deacon, S. E.; Tamber, S.; Firbank, S. J.; Mahmoud, K.; Knowles, P. F.; Phillips, S. E. V.; McPherson, M. J.; Dooley, D. M. The Stacking Tryptophan of Galactose Oxidase: A Second-Coordination Sphere Residue That Has Profound Effects on Tyrosyl Radical Behavior and Enzyme Catalysis '. *Biochemistry* **2007**, *46* (15), 4606–4618. https://doi.org/10.1021/bi062139d.

(2) Kumar, S.; Stecher, G.; Li, M.; Knyaz, C.; Tamura, K. MEGA X: Molecular Evolutionary Genetics Analysis across Computing Platforms. *Molecular Biology and Evolution* **2018**, *35* (6),

1547–1549. https://doi.org/10.1093/molbev/msy096.

(3) Risso, V. A.; Gavira, J. A.; Mejia-Carmona, D. F.; Gaucher, E. A.; Sanchez-Ruiz, J. M. Hyperstability and Substrate Promiscuity in Laboratory Resurrections of Precambrian β-Lactamases. *J. Am. Chem. Soc.* **2013**, *135* (8), 2899–2902. https://doi.org/10.1021/ja311630a.

(4) Madeira, F.; Pearce, M.; Tivey, A. R. N.; Basutkar, P.; Lee, J.; Edbali, O.; Madhusoodanan, N.; Kolesnikov, A.; Lopez, R. Search and Sequence Analysis Tools Services from EMBL-EBI in 2022. *Nucleic acids research* **2022**, *50* (W1), W276—W279. https://doi.org/10.1093/nar/gkac240.

(5) Varadi, M.; Anyango, S.; Deshpande, M.; Nair, S.; Natassia, C.; Yordanova, G.; Yuan, D.; Stroe, O.; Wood, G.; Laydon, A.; Žídek, A.; Green, T.; Tunyasuvunakool, K.; Petersen, S.; Jumper, J.; Clancy, E.; Green, R.; Vora, A.; Lutfi, M.; Figurnov, M.; Cowie, A.; Hobbs, N.; Kohli, P.; Kleywegt, G.; Birney, E.; Hassabis, D.; Velankar, S. AlphaFold Protein Structure Database: Massively Expanding the Structural Coverage of Protein-Sequence Space with High-Accuracy Models. *Nucleic Acids Research* **2022**, *50* (D1), D439–D444. https://doi.org/10.1093/nar/gkab1061.

(6) Waterhouse, A.; Bertoni, M.; Bienert, S.; Studer, G.; Tauriello, G.; Gumienny, R.; Heer, F. T.; de Beer, T. A. P.; Rempfer, C.; Bordoli, L.; Lepore, R.; Schwede, T. SWISS-MODEL: Homology Modelling of Protein Structures and Complexes. *Nucleic Acids Research* **2018**, *46* (W1), W296–W303. https://doi.org/10.1093/nar/gky427.

Supporting schemes, figures, tables and GOase sequences

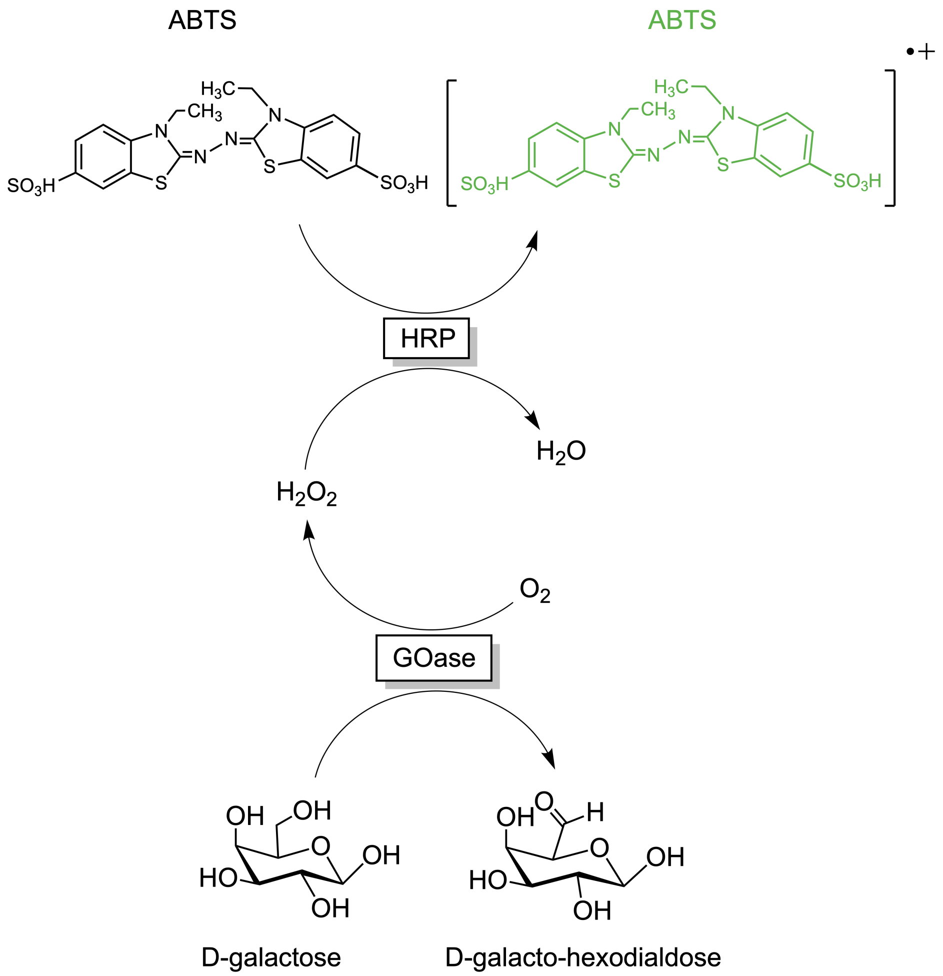

Scheme S1. Reaction cascade to measure GOase activity through HRP-ABTS coupled assay.

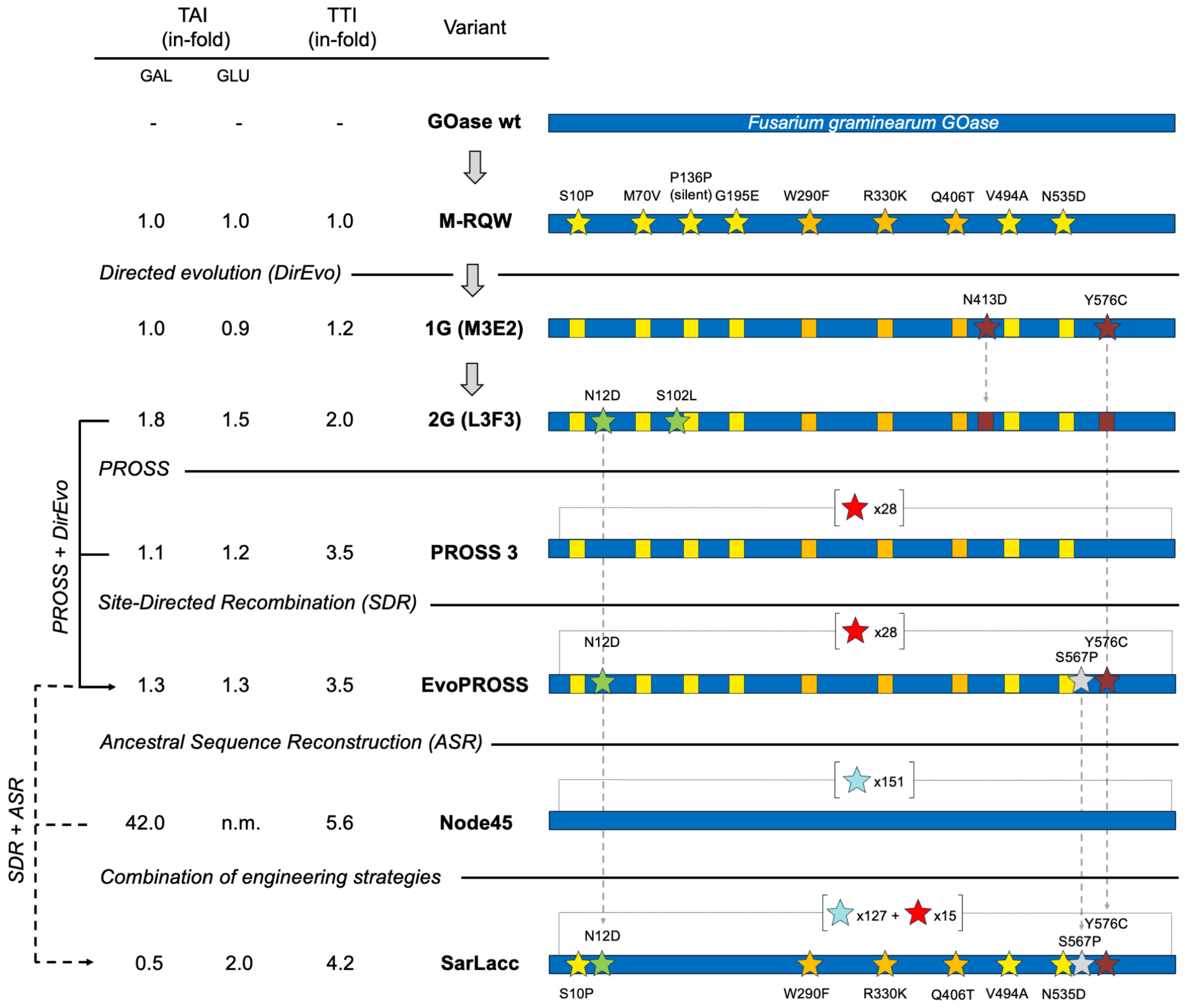

Figure S1. Overview of the protein engineering of this work. Stars indicate the introduction of new mutations, which are represented as squares once they are integrated in the GOase offspring. TAI: total activity improvement, TTI: total thermostability improvement, n.m.: non-measurable.

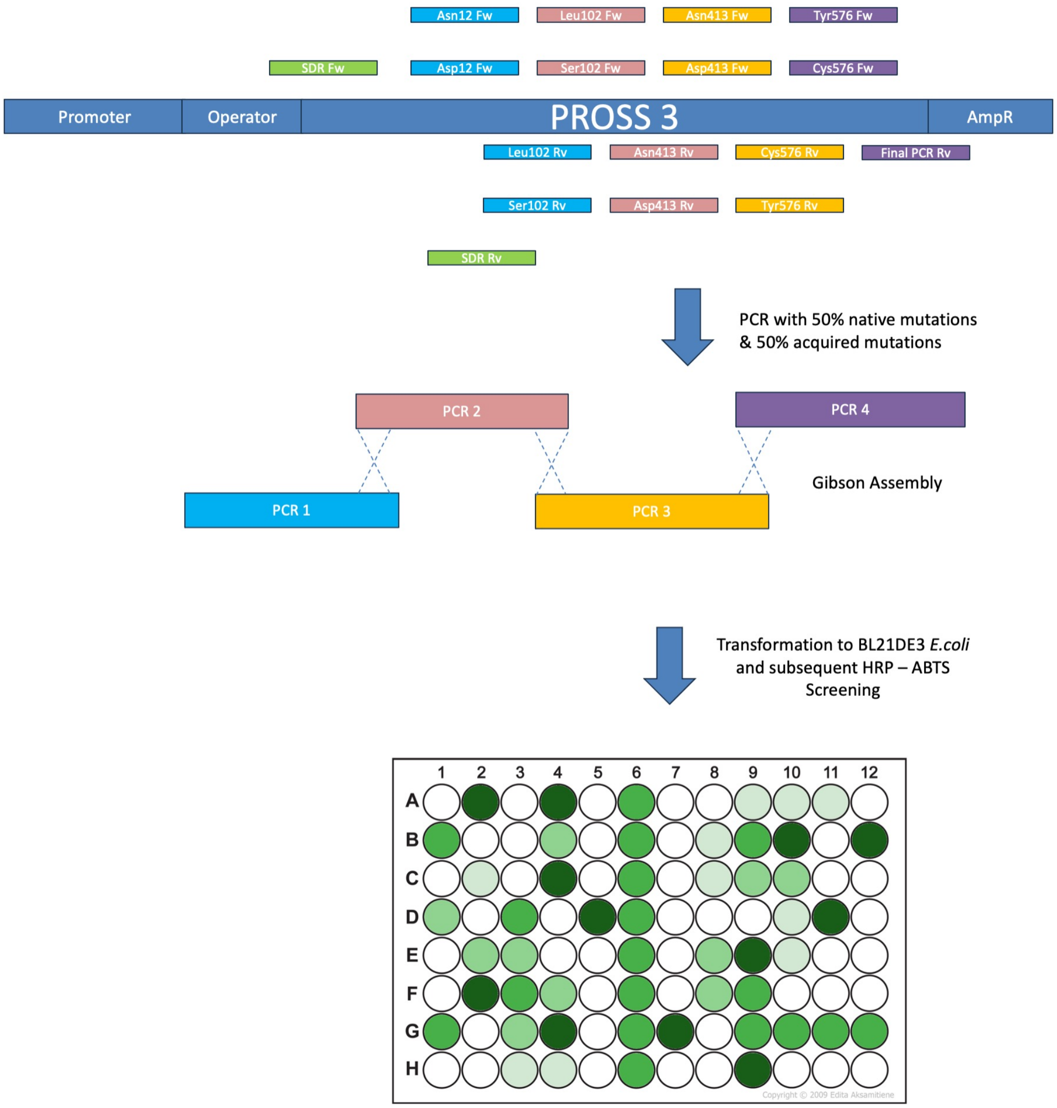

Figure S2. Overview of SDR library construction. Degenerated primers and PCR fragments were assembled by Gibson assembly and transformed into electrocompetent *E. coli* cells for library screening with the ABTS-HRP coupled assay.

Multiple sequence alignment (MSA) of M-RQW parent and selected engineered GOases in this work

```
M-RQW     ASAPIGSA-IPRNNWAVTCDSAQSGNECNKAIDGNKDTFWHTFYGANGDPKPPHTYTIDM  59
EvoPROSS  ASAPIGSA-IPRDNWAVTCDSAQSGNECNKAIDGNKDTFWHTFYGANGDPKPPHTYTIDM  59
Node45    AAPPIGNARIDRAGWKVTCDSEEPGNECSKAIDGDNNTFWHTAYSANGNPPPPHNITVDM  60
SarLacc   AAPPIGNARIPRDGWKVTCDSEEPGNECSKAIDGDNNTFWHTAYSANGNPPPPHNITVDM  60
Cons.     *: ***.* * * .* ***** : ****.*****:::***** *.***:* ***. *:**

M-RQW     KTTQNVNGLSVLPRQDGNQNGWIGRHEVYLSSDGTNWGSPVASGSWFADSTTKYSNFETR  119
EvoPROSS  GTTHNVNGLSVLPRQDGNQNGWIGRHEVYLSSDGTNWGSPVASGMWFADSTTKYSNFEPR  119
Node45    GSTQNVNGLSVLPRQDGNQNGWIARHEVYVSTDGTNWGDPVATGTWYADSTTKYANFEPR  120
SarLacc   GSTHNVNGLSVLPRQDGNQNGWIARHEVYVSTDGTNWGDPVASGMWYADSTTKYANFEPR  120
Cons.      :*:*******************.*****:*:******.***:* *:*******:*** *

M-RQW     PARYVRLVAITEANGQPWTSIAEINVFQASSYTAPQPGLGRWGPTIDLPIVPAAAAIEPT  179
EvoPROSS  PARYVRLVAITEANGQPWTSIAEINVFQASSYTPPQPGLGRWGPTIDLPIVPAAAAIEPT  179
Node45    SARYVRLVALSEANGNPWTSIAELNVYKANTEPAPAAGLGKWGPTIDFPIVPVAGAVDPL  180
SarLacc   SARYVRLVALSEANGNPWTSIAELNVYKANTEPPPAAGLGKWGPTIDFPIVPVAGAVDPL  180
Cons.      ********::****:*******:**::*.:   *  ***:******:****.*.*::*

M-RQW     SGRVLMWSSYRNDAFEGSPGGITLTSSWDPSTGIVSDRTVTVTKHDMFCPGISMDGNGQI  239
EvoPROSS  SGRVLMWSSYRNDQFEGSPGGITLTSIWDPSTGIVSDRTVTNTKHDMFCPGISMDGNGQI  239
Node45    TGKVLVWSSYANDNFEGSPGGRTLTSTWDPATGDVTQRIVTNTDHDMFCPGISMDGNGQI  240
SarLacc   TGKVLVWSSYANDQFEGSPGGRTLTSIWDPATGDVTQRIVTNTDHDMFCPGISMDGNGQI  240
Cons.     :*:**:**** ** ******* **** ***:** *::* ** *.****************

M-RQW     VVTGGNDAKKTSLYDSSSDSWIPGPDMQVARGYQSSATMSDGRVFTIGGSFSGG-VFEKN  298
EvoPROSS  VVTGGNDAKKTSLYDSSSDSWIPGPDMQVPRGYQSSATLSDGRVFTIGGSFSGG-VFEKN  298
Node45    VVTGGNDAQKTSLYDSASDSWIPGPDMKVARGYQSSATCSDGRVFTIGGSWSGGEVFEKN  300
SarLacc   VVTGGNDAQKTSLYDSASDSWIPGPDMKVPRGYQSSATLSDGRVFTIGGSFSGGEVFEKN  300
Cons.     ********:*******:**********:* ******** ***********:*** *****

M-RQW     GEVYSPSSKTWTSLPNAKVNPMLTADKQGLYKSDNHAWLFGWKKGSVFQAGPSTAMNWYY  358
EvoPROSS  GEVYDPSSNTWTLLPNAKVNPMLTNDKQGLYKSDNHAWLFGWKNGSVFQAGPSTAMNWYY  358
Node45    GEIYDPSTNTWTMLPGAKVKPMLTADKQGIYRADNHAWLFGWKNGSVFQAGPSTAMNWYY  360
SarLacc   GEIYDPSTNTWTLLPGAKVKPMLTNDKQGIYKADNHAWLFGWKNGSVFQAGPSTAMNWYY  360
Cons.     **:*.**::*** **.***:**** ****:*::**********:****************

M-RQW     TSGSGDVKSAGKRQSNRGVAPDAMCGNAVMYDAVKGKILTFGGSPDYTDSDATTNAHIIT  418
EvoPROSS  TSGSGDVKSAGKRQSNRGVDPDAMCGNAVMYDAVKGKILTFGGSPDYTDSDATTNAHIIT  418
Node45    TSGNGNVKSAGKRKSNRGDDPDSMCGNAVMYDAVAGKILTFGGSPSYQDSDATTNAHIIT  420
SarLacc   TSGNGNVKSAGKRKSNRGDDPDSMCGNAVMYDAVAGKILTFGGSPSYTDSDATTNAHIIT  420
Cons.     ***.*:*******:****  **:*********** **********.* ************
```

```
M-RQW    LGEPGTSPNTVFASNGLYFARTFHTSVVLPDGSTFITGGQRRGIPFEDSTPVFTPEIYVP  478
EvoPROSS LGEPGTSPNVVFASNGLWFARTFHTSVVLPDGSVFITGGQRRGIPFEDSTPVFTPEIYDP  478
Node45   IGDPGATPSVTFASNGMHYARVFHTSVVLPDGTVFITGGQSYAIPFTDSTPQFTPELYDP  480
SarLacc  IGDPGATPSVTFASNGMWYARVFHTSVVLPDGTVFITGGQSYAIPFTDSTPQFTPELYDP  480
Cons.    :*:**::*...*****: :**.**********:.******  .*** **** ****:* *

M-RQW    EQDTFYKQNPNSIVRAYHSISLLLPDGRVFNGGGGLCGDCTTNHFDAQIFTPNYLYDSNG  538
EvoPROSS EQDTFYKQNPNSIPRAYHSVSLLLPDGRVFNGGGGLCGDCTTNHFDAQIFTPPYLFDSNG  538
Node45   EQDTFVEQAPNSIVRVYHSISLLLPDATVFNGGGGLCGDCNTNHFDAQIFTPSYLFNRDG  540
SarLacc  EQDTFVEQAPNSIPRAYHSVSLLLPDATVFNGGGGLCGDCNTNHFDAQIFTPPYLFDRDG  540
Cons.    ***** :* **** *.***:******. ************.*********** **:: :*

M-RQW    NLATRPKITRTSTQSVKVGGRITISTDSSISKASLIRYGTATHTVNTDQRRIPLTLTNNG  598
EvoPROSS NLATRPKITSTSTDSVKVGGRITIQTDSPISKASLIRCGTATHTVNTDQRRIPLTLTNNG  598
Node45   SPAARPVITSVSTSSVKVGGRLTITTDSAVTSASLIRYGTATHTVNTDQRRIPLTLTSSG  600
SarLacc  SPAARPVITSVSTDSVKVGGRLTIQTDSPVTSASLIRCGTATHTVNTDQRRIPLTLTSSG  600
Cons.    . *:** ** .**.*******:** *** ::.***** *******************..*

M-RQW    GNSYSFQVPSDSGVALPGYWMLFVMNSAGVPSVASTIRVTQ 639
EvoPROSS GNSYSFQVPSDSGVALPGYWMLFVMNSAGVPSVAKTIRVTQ 639
Node45   TNSYTAQLPNDPGILLPGYWMLFVMNSNGVPSVAKTIKVLL 641
SarLacc  TNSYTAQLPNDPGILLPGYWMLFVMNSNGVPSVAKTIKVLL 641
Cons.     ***: *:*.* *: ************ ******.**:*
```

Identity matrix of M-RQW parent and engineered GOases in this work

	M-RQW	EvoPROSS	Node45	SarLacc
M-RQW	100.0	95.2	76.2	76.2
EvoPROSS	95.2	100.0	77.2	81.1
Node45	76.2	77.2	100.0	96.1
SarLacc	76.2	81.1	96.1	100.00

Figure S3. MSA and identity matrix of engineered GOases in this work compared with M-RQW parental type. The different mutations from engineered GOases compared to M-RQW parent are highlighted for each variant using their corresponding color. Mutations marked in bold were introduced into node 45 to design SarLacc. Black arrows represent the mutations introduced from M-RQW into SarLacc. Yellow arrows indicate the mutations introduced into SarLacc from EvoPROSS, which were acquired by SDR, while purple arrows show EvoPROSS mutations from PROSS3 introduced into node 45 for engineering SarLacc. The identity matrix represents sequence identities as percentages. Cons.: consensus sequence.

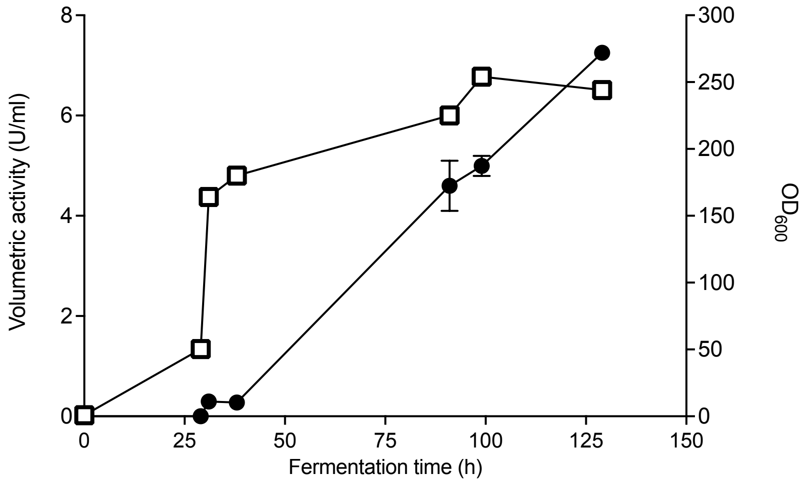

Figure S4. *P. pastoris* M-RQW variant production in 10 L fed-batch bioreactor. Fermentation was monitored over the course of 128 hrs. Samples were taken twice a day and measured in technical duplicates. Black circles: OD600, white squares: volumetric activity (U/mL) measured with ABTS-HRP assay.

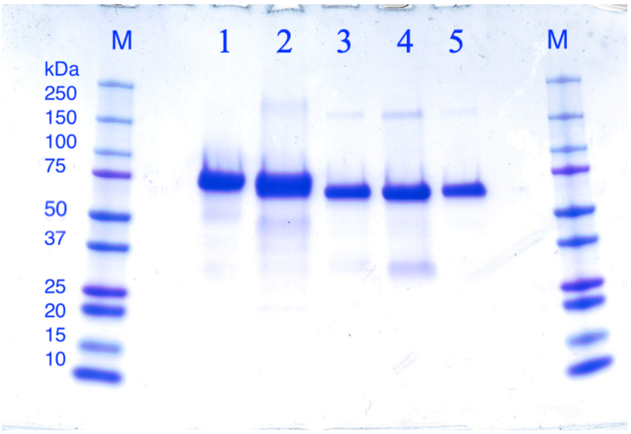

Figure S5. SDS-PAGE of purified *P. pastoris* GOase variants with His-tag using IMAC. Proteins were run in a gradient gel of 4-20% and stained with Coomassie blue R-250. The running buffer was Tris-Glycine pH 8.6 with 0.1% SDS. M: molecular weight marker, lane 1: M-RQW-His, lane 2: PROSS 3-His, lane 3: EvoPROSS-His, lane 4: node 45-His, and lane 5: SarLacc-His.

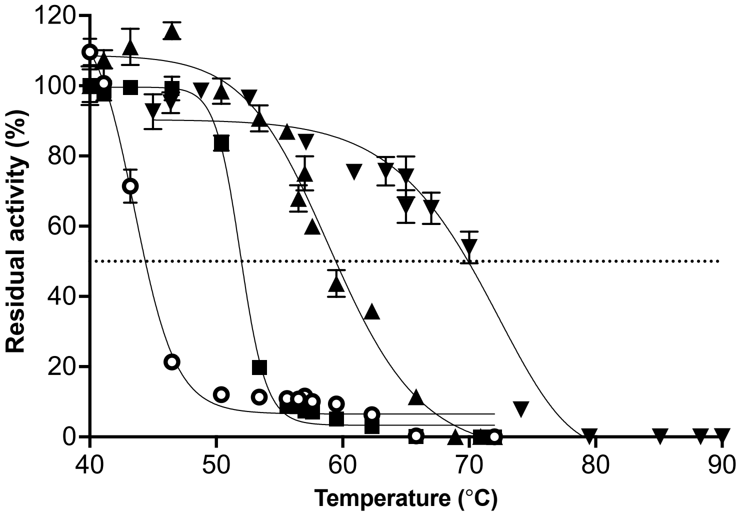

Figure S6. Kinetic thermostability of engineered GOase variants. M-RQW (white circles), EvoPROSS (black squares), SarLacc (black triangles up) and node 45 (black triangles down) variants were expressed in *P. pastoris* and purified by IMAC. Each purified variant was assayed in a gradient of temperature from 40 ºC to 90 ºC. Residual activities were measured by using the HRP-ABTS coupled assay. The dotted line at 50% of residual activity is represented as a reference for the T_{50} value. Each point, including the standard deviation, is from three independent experiments.

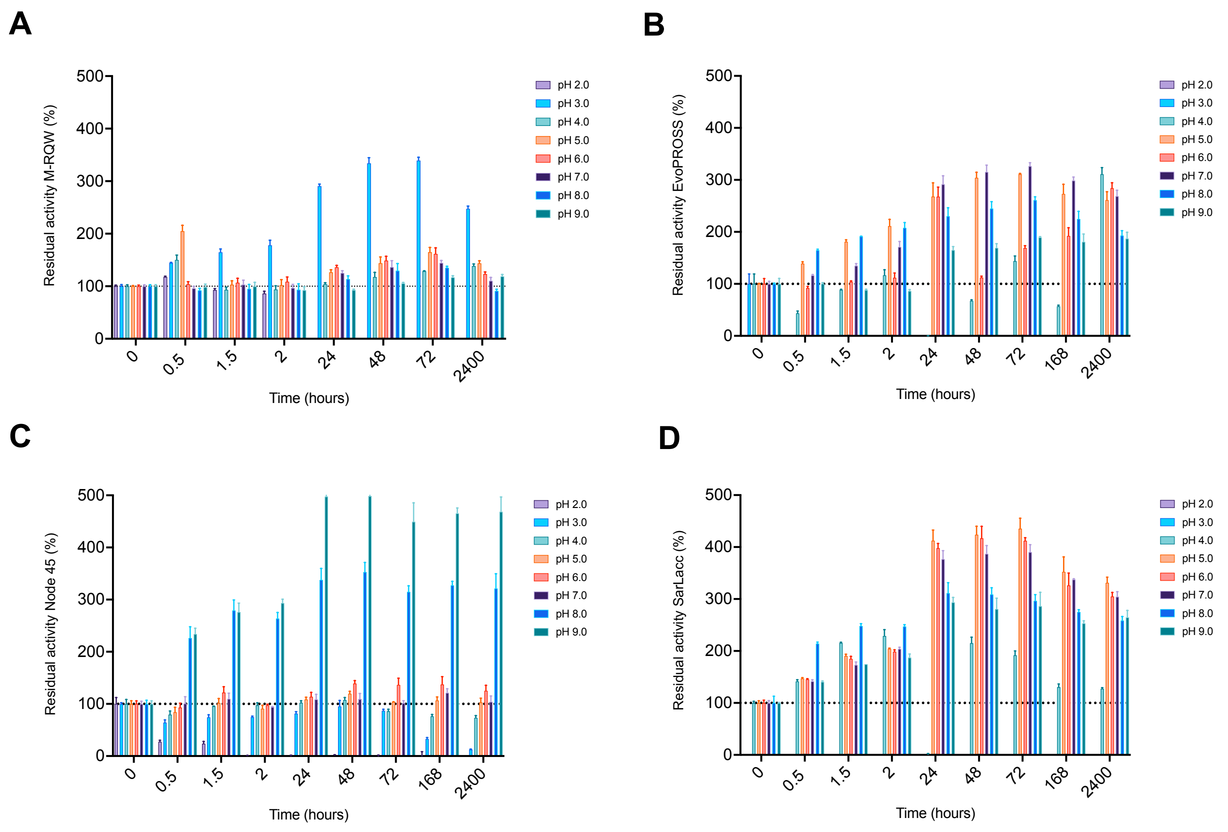

Figure S7. pH-dependent stability over the course of 10 days at 4°C with M-RQW (**A**), EvoPROSS (**B**), node 45 (**C**), and SarLacc (**D**). Activity measurements were run at room temperature in kinetic mode by using 20 µL of enzyme incubations and 180 µL 100 mM NaPi buffer pH 7.0 containing 0.5 mM ABTS, 7 U/mL HRP, and 100 mM galactose as a substrate. The reaction was recorded at 418 nm in a plate reader. Enzyme hyperactivation at different pHs and incubation times was observed. Each point, including the standard deviation, is from three independent experiments.

Benzyl alcohol

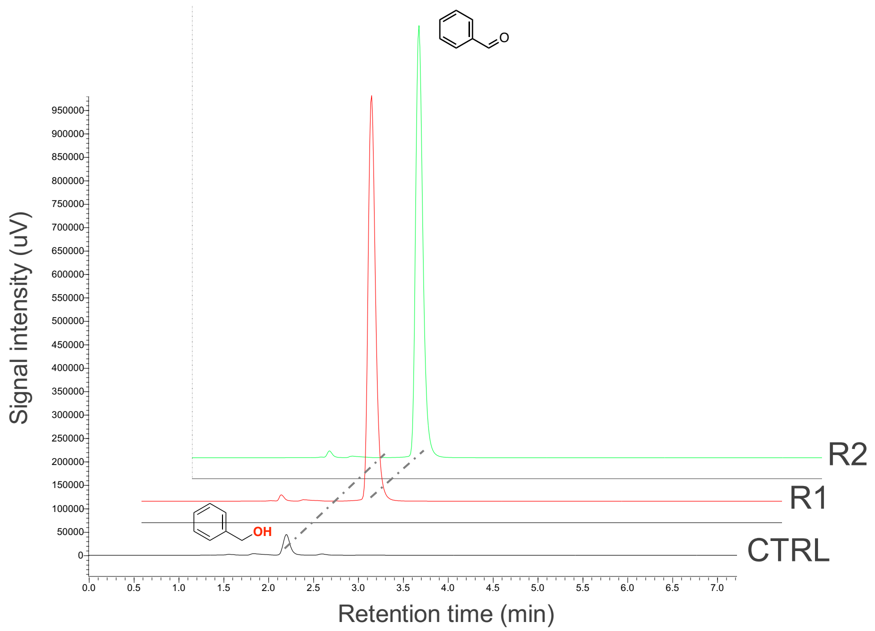

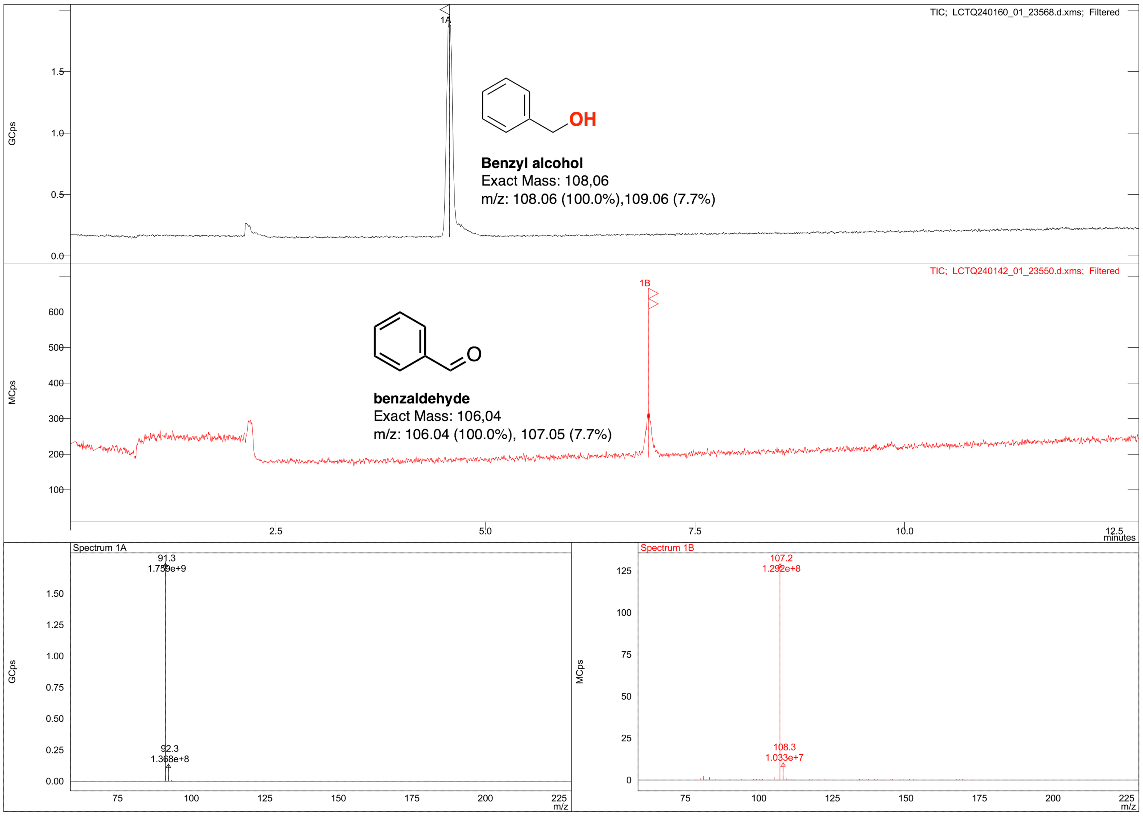

Figure S8. HPLC-MS analysis of benzyl alcohol. CTRL: control reaction in the absence of GOase, R: reaction in the presence of GOase (SarLacc). Reactions were run in duplicates (R1, R2).

1-phenylethanol

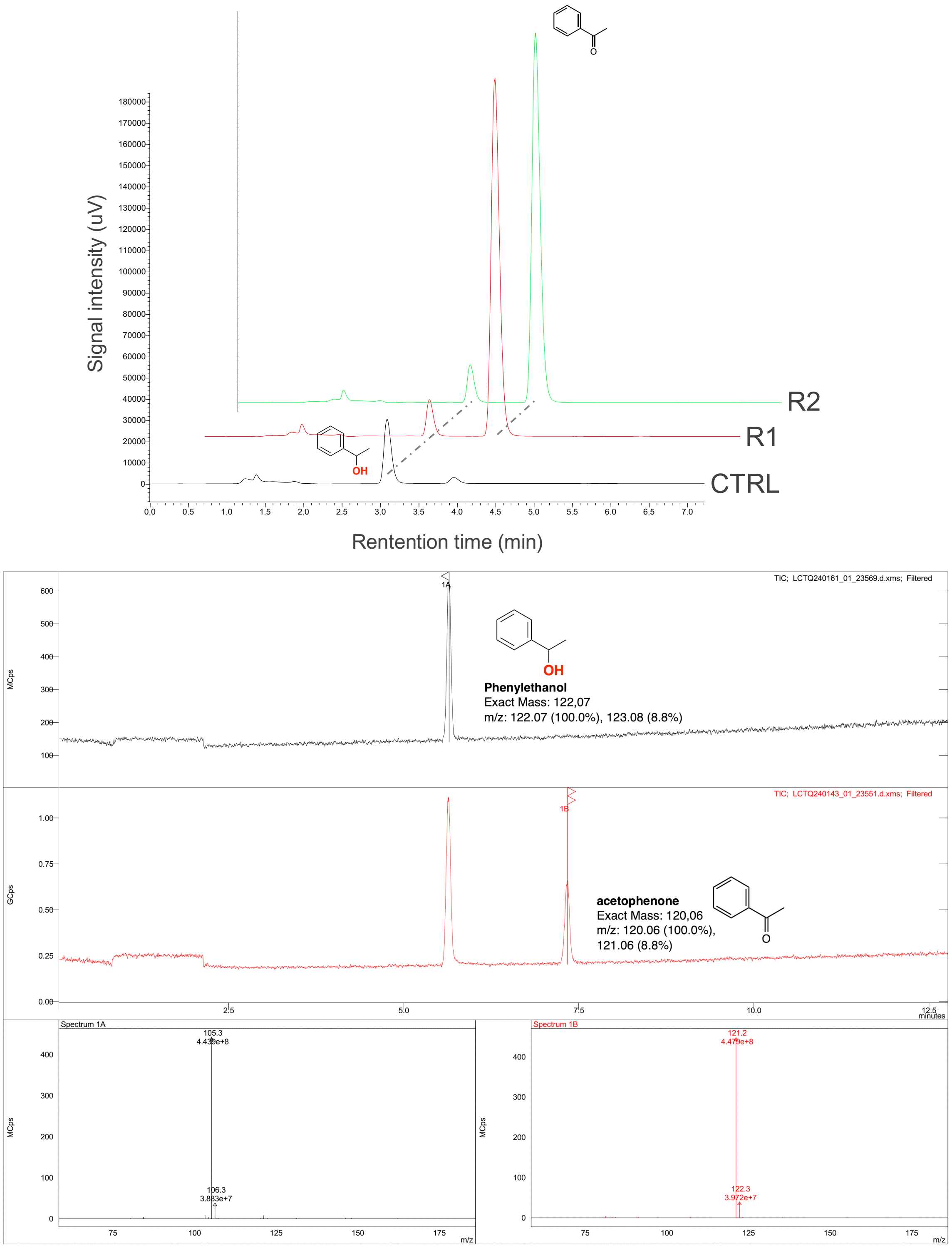

Figure S9. HPLC-MS analysis of 1-phenylethanol. CNTRL: control reaction in the absence of GOase, R: reaction in the presence of GOase (SarLacc). Reactions were run in duplicates (R1, R2).

1-phenylpropanol

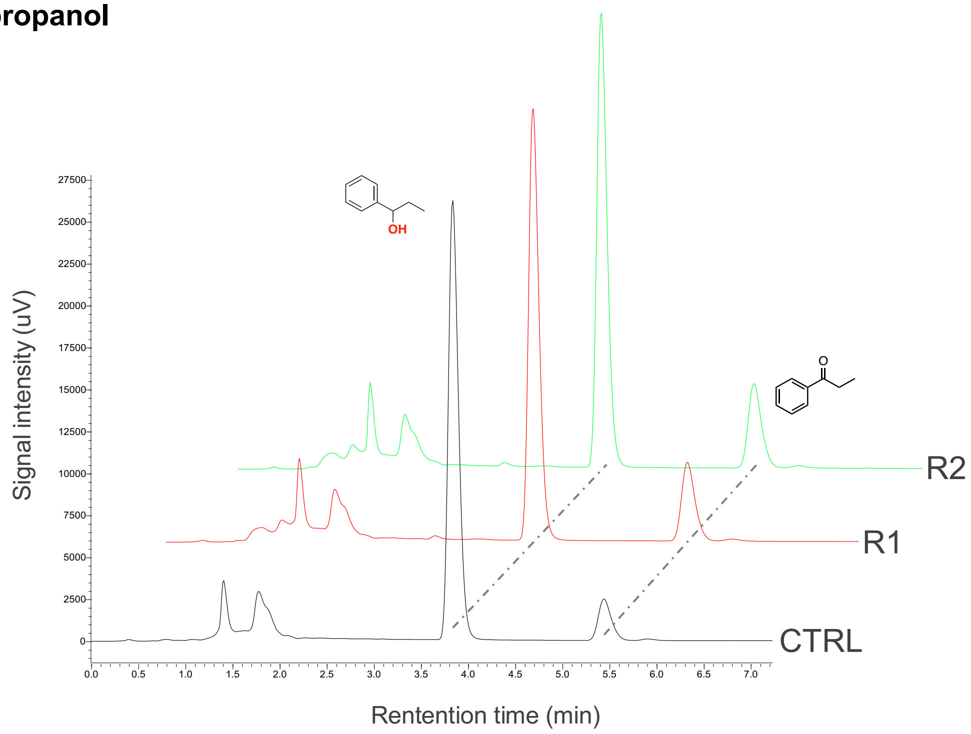

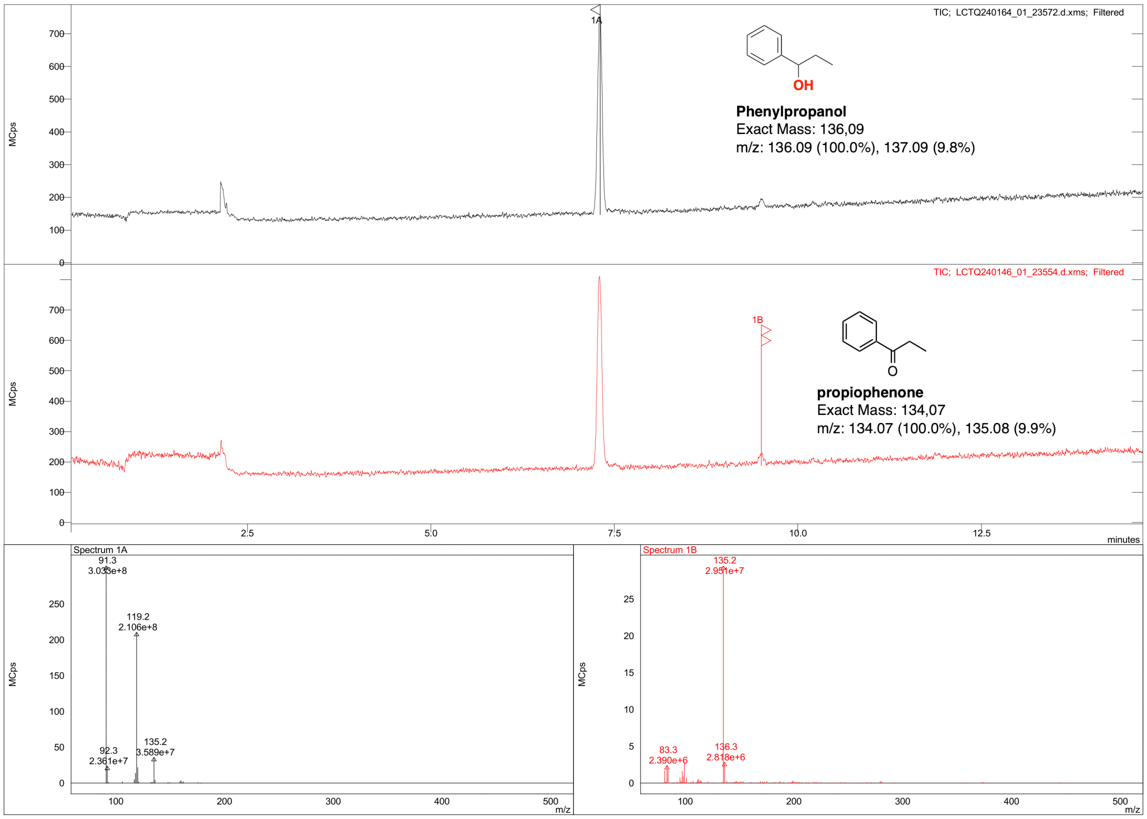

Figure S10. HPLC-MS analysis of 1-phenylpropanol. CTRL: control reaction in the absence of GOase, R: reaction in the presence of GOase (SarLacc). Reactions were run in duplicates (R1, R2).

1-phenylbutanol

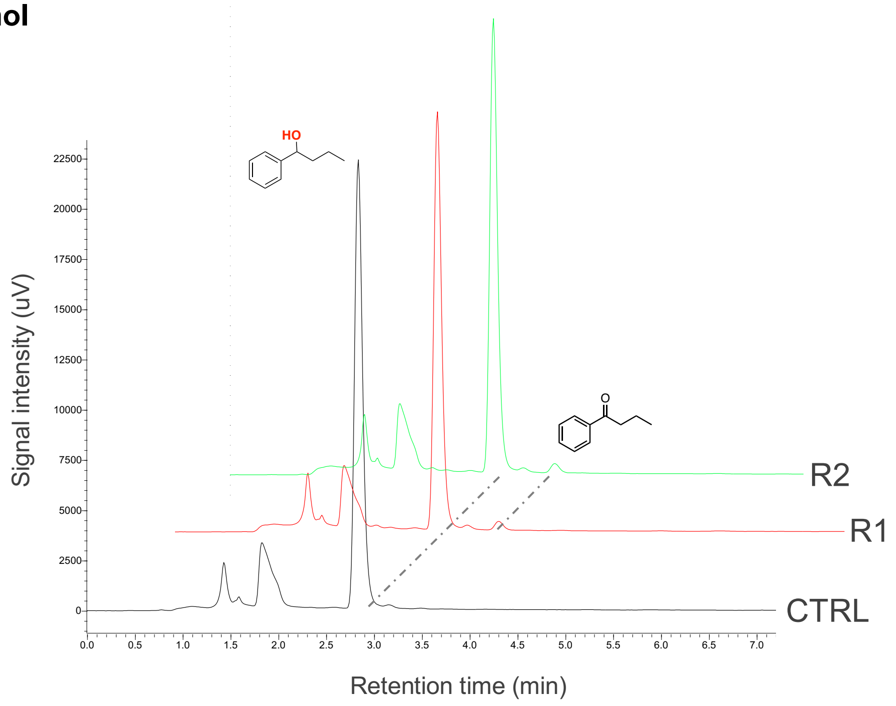

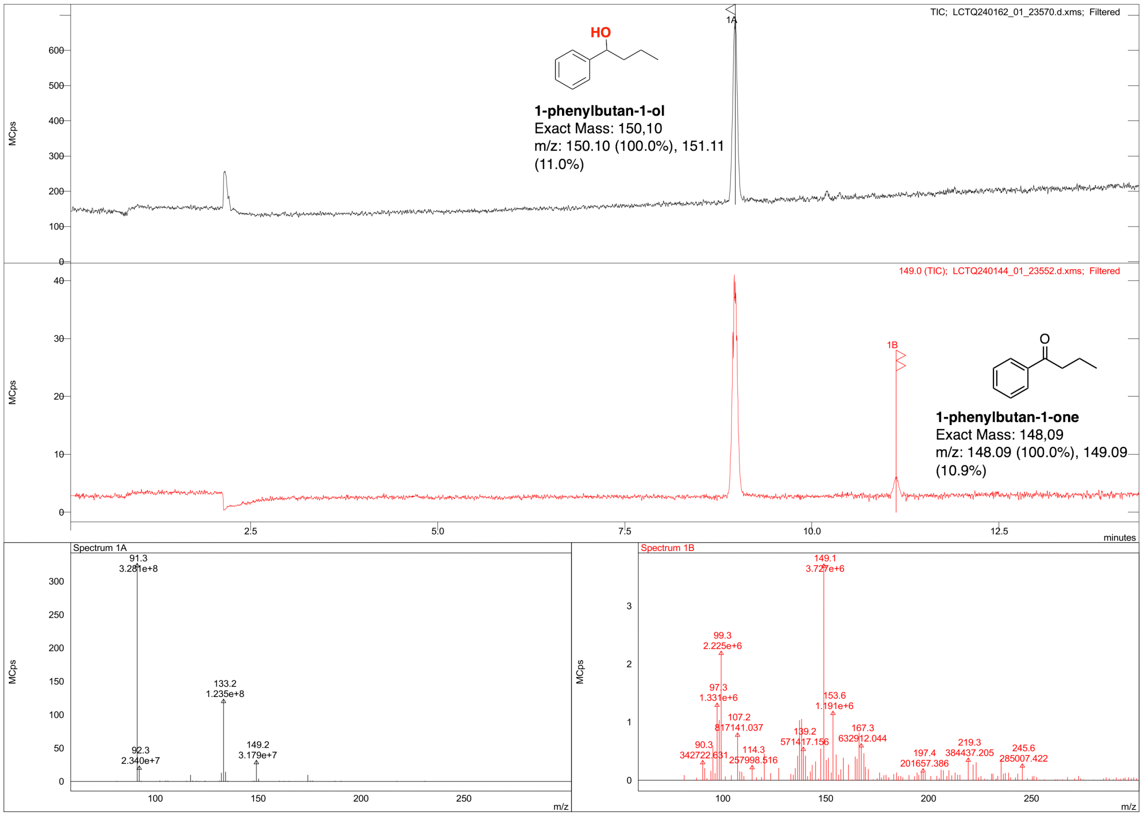

Figure S11. HPLC-MS analysis of 1-phenylbutanol. CTRL: control reaction in the absence of GOase, R: reaction in the presence of GOase (SarLacc). Reactions were run in duplicates (R1, R2).

1-phenylpentanol

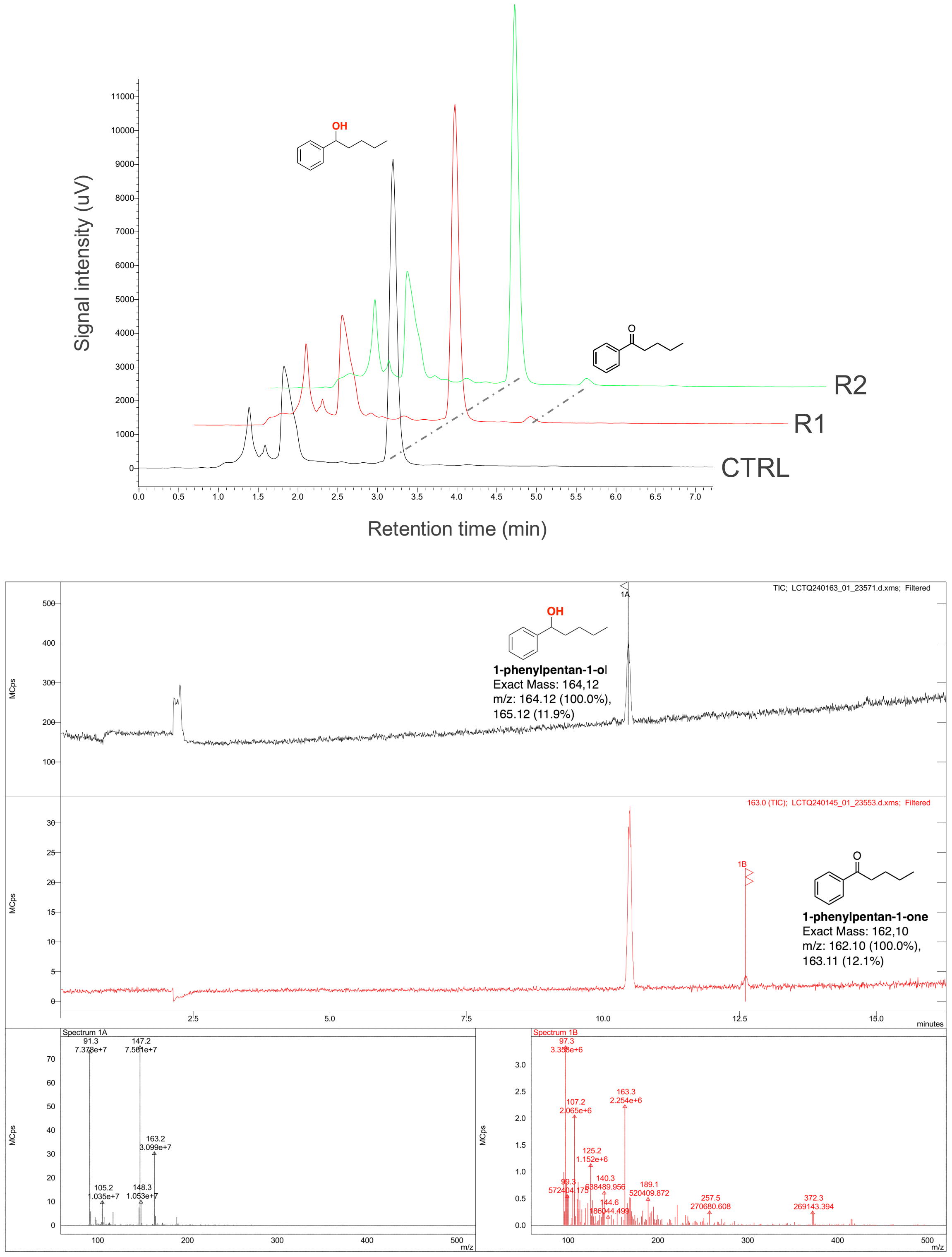

Figure S12. HPLC-MS analysis of 1-phenylpentanol. CTRL: control reaction in the absence of GOase, R: reaction in the presence of GOase (SarLacc). Reactions were run in duplicates (R1,R2).

Alpha-tetralol

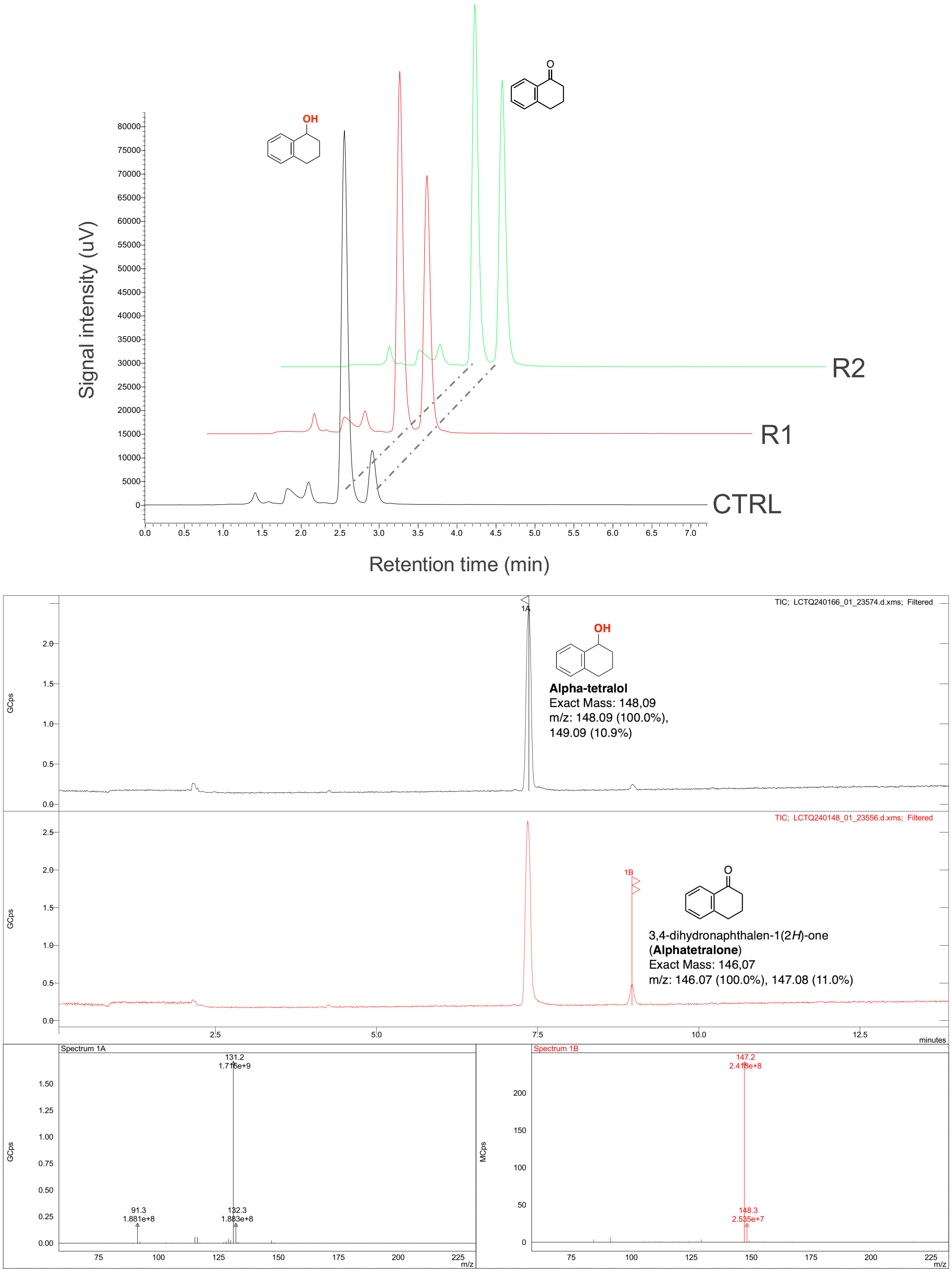

Figure S13. HPLC-MS analysis of alpha-tetralol. CTRL: control reaction in the absence of GOase, R: reaction in the presence of GOase (SarLacc). Reactions were run in duplicates (R1, R2).

Diphenylmethanol

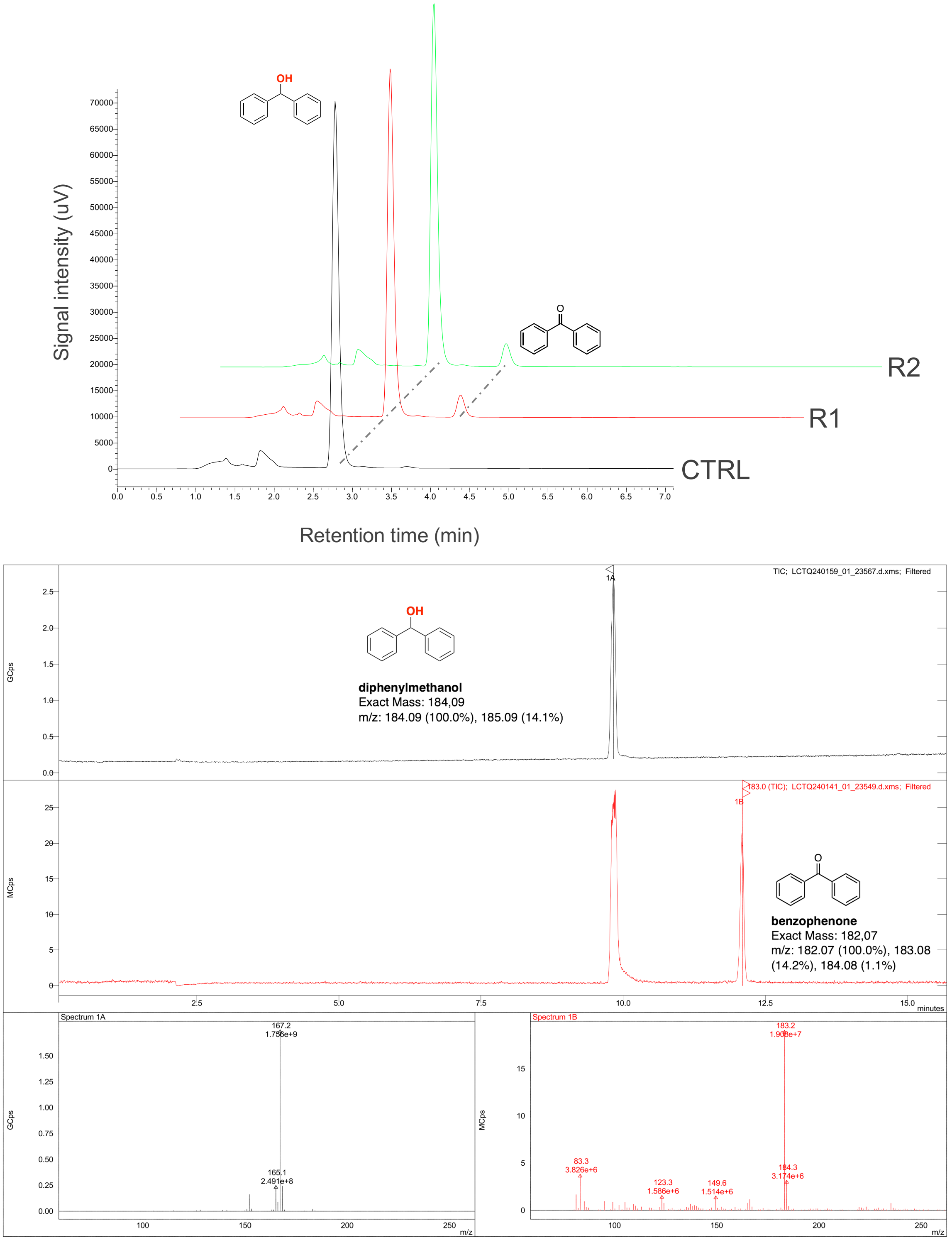

Figure S14. HPLC-MS analysis of diphenylmethanol. CTRL: control reaction in the absence of GOase, R: reaction in the presence of GOase (SarLacc). Reactions were run in duplicates (R1, R2).

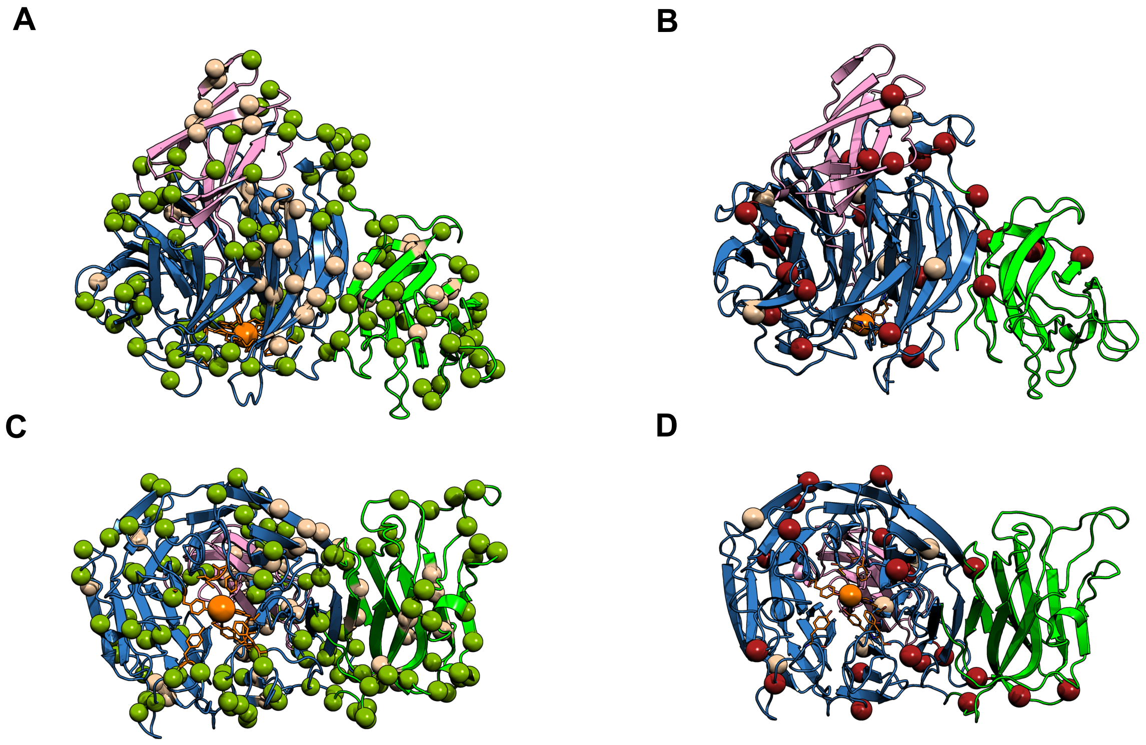

Figure S15. Side and bottom views of mutations found in β-strands of GOase structure for node 45 (**A**, **C**) and PROSS 3 (**B**, **D**). Spheres in wheat color indicate mutations within β-strands. Green spheres and red spheres show node 45 and PROSS mutations outside of β-strands, respectively. Orange spheres and sticks represent the copper ion and amino acids in GOase active site.

Table S1. Mutations of different PROSS designs compared to M-RQW parental type.

Variant	Number of mutations	Mutations	Approach
M-RQW *vs.* GOase_wt	9	S10P, M70V, *P136 (silent),* G195E, W290F, R330K, Q406T, V494A, N535D	Directed evolution
M-RQW *vs.* PROSS 1	13	Q63H, S104M, S206I, V221N, A269P, S303D, K342N, T428V, T452V, V492P, N531P, R548S, S563Q	PROSS
M-RQW *vs.* PROSS 3	28	K60G, Q63H, S104M, T118P, A153P, A193Q, S206I, V221N, A269P, M278L, S303D, K307N, S311L, A323N, K342N, A378D, T428V, Y436W, T452V, V477D, V492P, I498V, N531P, Y534F, R548S, Q552D, S563Q, S633K	PROSS
M-RQW *vs.* PROSS 4	39	A15T, K60G, Q63H, S91T, S104M, F106Y, T118P, A153P, A193Q, S205A, S206I, V221N, K223H, S255P, A269P, M278L, S303D, S306T, K307N, S311L, A323N, K342N, A378D, D404H, T428V, Y436W, T452V, V477D, V492P, I498V, N531P, Y534F, L540P, R548S, T549V, Q552D, S563Q, S569K, S633K	PROSS

Table S2. Mutations of different GOases engineered in this work compared to M-RQW parental type.

Variant	Number of mutations	Mutations*	Approach/Notes
M-RQW *vs.* GOase_wt	9	S10P, M70V, *P136 (silent),* G195E, W290F, R330K, Q406T, V494A, N535D	Directed evolution
M-RQW *vs.* PROSS 3	28	K60G, Q63H, S104M, T118P, A153P, A193Q, S206I, V221N, A269P, M278L, S303D, K307N, S311L, A323N, K342N, A378D, T428V, Y436W, T452V, V477D, V492P, I498V, N531P, Y534F, R548S, Q552D, S563Q, S633K	PROSS
M-RQW *vs.* EvoPROSS	31	N12D, K60G, Q63H, S104M, T118P, A153P, A193Q, S206I, V221N, A269P, M278L, S303D, K307N, S311L, A323N, K342N, A378D, T428V, Y436W, T452V, V477D, V492P, I498V, N531P, Y534F, R548S, Q552D, S563Q, S567P, Y576C, S633K	Combination of PROSS and SDR<br><br>- This mutant incorporated one unexpected mutation during PCR amplification (S567P)
M-RQW *vs.* node 45	151<br>(+ 2 insertions)	S2A, A3P, S7N, P10D, N12A, N13G, A15K, A21E, Q22E, S23P, N28S, N34D, K35N, D36N, F42A, G44S, D48N, K50P, T54N, Y55I, I57V, K60G, T61S, G83A, L89V, S91T, S98D, S102T, S104T, F106Y, S114A, T118P, P120S, I129L, T130S, Q135N, I143L, F146Y, Q147K, S149N, S150T, Y151E, T152P, Q155A, P156A, R160K, L167F, A172V, A174G, I176V, E177D, T179L, S180T, R182K, M185V, R190A, A193N, I201R, S206T, S210A, I213D, S215T, D216Q, T218I, V221N, K223D, K248Q, S256A, Q267K, M278C, F290W, V301I, S303D, S306T, K307N, S311M, N314G, N318K, L328I, K330R, S331A, K342N, S362N, D364N, Q372K, V377D, A378D, A382S, K393A, D404S, T406Q, L419I, E421D, T424A, S425T, N427S, T428V, V429T, L435M, Y436H, F437Y, T440V, S451T, T452V, R459S, R460Y, G461A,	ASR

		E465T, V470Q, I475L, V477D, Y484V, K485E, N487A, A494V, G505A, R506T, T519N, N531S, Y534F, D535N, S536R, N537D, N539S, L540P, T542A, K545V, R548S, T549V, Q552S, I560L, S563T, S567A, I568V, S569T, K570S, N596S, N597S, G599T, S603T, F604A, V606L, S608N, S610P, V612I, A613L, A626N, S633K, R636K, T638L, Q639L	
M-RQW *vs.* SarLacc	151<br>(+2 insertions)	S2A, A3P, S7N, N12D, N13G, A15K, A21E, Q22E, S23P, N28S, N34D, K35N, D36N, F42A, G44S, D48N, K50P, T54N, Y55I, I57V, K60G, T61S, Q63H, G83A, L89V, S91T, S98D, S104M, F106Y, S114A, T118P, P120S, I129L, T130S, Q135N, I143L, F146Y, Q147K, S149N, S150T, Y151E, T152P, A153P, Q155A, P156A, R160K, L167F, A172V, A174G, I176V, E177D, T179L, S180T, R182K, M185V, R190A, A193Q, I201R, S206I, S210A, I213D, S215T, D216Q, T218I, V221N, K223D, K248Q, S256A, Q267K, A269P, M278L, V301I, S303D, S306T, K307N, S311L, N314G, N318K, A323N, L328I, S331A, K342N, S362N, D364N, Q372K, V377D, A378D, A381S, K393A, D404S, L419I, E421D, T424A, S425T, N427S, T428V, V429T, L435M, Y436W, F437Y, T440V, S451T, T452V, R459S, R460Y, G461A, E465T, V470Q, I475L, V477D, Y484V, K485E, N487A, V492P, I498V, G505A, R506T, T519N, N531P, Y534F, S536R, N537D, N539S, L540P, T542A, K545V, R548S, T549V, Q552D, I560L, S563Q, S567P, I568V, S569T, K570S, Y576C, N596S, N597S, G599T, S603T, F604A, V606L, S609N, S610P, V612I, A613L, A626N, S633K, R636K, T638L, Q639L	Combination of SDR+PROSS (EvoPROSS) and ASR (node 45)<br><br>- From the 9 mutations of M-RQW, 3 mutations were already present in node 45 (in bold): S10P, **M70V**, ***P136P (silent)***, **G195E**, W290F, R330K, Q406T, V494A, N535D. Therefore, the remaining 6 mutations were introduced into node 45.<br><br>- From the 28 mutations of PROSS3, 13 mutations were already in node 45 (in bold): **K60G**, Q63H, S104M, **T118P**, A153P, A193Q, S206I, **V221N**, A269P, M278L, **S303D**, **K307N**, S311L, A323N, **K342N**, **A378D**, **T428V**, Y436W, **T452V**, **V477D**, V492P, I498V, N531P, **Y534F**, **R548S**, Q552D, S563Q, **S633K.** Therefore, the

			remaining 15 mutations were introduced into Node45.<br><br>-From the 3 mutations of EvoPROSS: N12D, S567P, Y576C. All 3 mutations were introduced into node 45.<br><br>**In total 24 substitutions were introduced into node 45 to generate SarLacc.**

*The numbering of mutations shown in this table is referred to M-RQW parental type. ASR nodes contain 641 amino acid residues in comparison to 639 found in M-RQW; therefore, in ASR there are two insertions between positions 8/9, and between positions 293/294.

Table S3. Mutations of ancestral nodes compared to GOase wt.

Variant	Number of mutations	Mutations*
GOase_wt *vs.* node 39	186	S2A, I5S, S10D, N13G, A15T, V16A, S23P, N28S, K29N, I31L, N34D, K35T, D36N, F42E, G44D, A45P, N46T, D48N, P49A, K50P, P51L, T54N, Y55I, T61A, Q63Y, L68V, M70Y, Q78S, W81N, R84Q, L89V, S91T, S102T, S104T, F106L, A107N, Y113T, S115A, T118P, P120S, I129L, T130S, Q135N, I140A, V145I, F146Y, Q147T, T152I, Q155P, P156S, L158K, R160K, L167F, A172V, I176V, S180T, R182K, M185V, R190A, N191P, A193Q, I201Q, S205A, S206T, W207Y, S210A, I213T, D216Q, T218I, V221N, K223N, G235F, Q238R, K248Q, L252I, S255P, I261T, P262S, Q267K, V268I, A276T, M278C, V283I, V294E, F295G, E296G, V301I, S303N, K307N, S311L, N314G, N318A, A323N, K325R, L328V, S331A, A335G, K342N, T352K, Y358N, S362N, D364S, K366T, S367G, V377D, A378D, P379A, A381S, K393A, F399V, D404N, S408A, L419I, E421N, T424A, S425T, N427T, T428V, V429T, F430K, S432A, N433S, L435M, Y436S, F437Y, T440I, T443N, S451T, T452V, R459S, R460Y, I462E, E465T, S467T, P469A, V470E, I475L, Y476W, V477N, E479A, Q480T, D481N, Y484T, K485Q, Q486M, N487A, V492P, V494T, S499A, G505A, R506T, F508L, D517N, T519S, N531P, Y534F, S536A, N537D, N539S, L540R, K545V, R548S, T549V, Q552T, R559T, I560L, S563T, D565N, S567A, I568V, S569T, K570S, I574V, N596S, N597S, G599T, S603T, F604V, Q605T, V606I, S610P, A626N, V631I, S633K, R636K, Q639P
GOase_wt *vs.* node 40	185	S2A, I5S, S10D, N13G, A15T, V16A, S23P, N28S, K29N, N34D, K35T, D36N, F42E, G44D, A45P, N46T, D48N, P49A, K50P, P51L, T54N, Y55I, T61A, Q63Y, L68V, M70Y, Q78S, W81N, R84Q, L89V, S91T, S103T, S105T, F106L, A107D, Y113T, S115A, T118P, P120S, I129L, T130S, Q135N, I140A, V145I, F146Y, Q147T, T152I, Q155P, P156S, R160K, L167F, A172V, I176V, S180T, R182K, M185V, R190A, N191P, A193Q, I201Q, S205A, S206T, W207Y, S210A, I213T, D216Q, T218I, V221N, K223N, G235F, Q238R, K248Q, L252I, S255P, I261T, P262S, Q267K, V268I, A276T, M278C, V283I, V294E, F295G, E296G, V301I, S303N, K307N, S311L, N314G, N318A, A323N, K325R, L328V, S331A, A335G, K342N, T352K, Y358N, S362N, D364S, K366T, S367G, V377D, A378D, P379A, A381S, K393A, F399V, D404N, S408A, L419I, E421N, T424A, S425T, N427S, T428V, V429T, F430K, S432A, N433S, L435M, Y436S, F437Y, T440I, T443N, S451T, T452V, R459S, R460Y, I462E, E465T, S467T, P469A, V470E, I475L,

		Y476W, V477N, E479A, Q480T, D481N, Y484T, K485Q, Q486M, N487A, V492P, V494T, S499A, G505A, R506T, F508L, D517N, T519S, N531P, Y534F, S536A, N537D, N539S, L540P, K545V, R548S, T549V, Q552T, R559T, I560L, S563T, D565N, S567A, I568V, S569T, K570S, I574V, N596S, N597S, G599T, S603T, F604V, Q605T, V606I, S608N, S610P, A626N, V631I, S633K, R636K, Q639P
GOase_wt *vs.* node 41	142	S2A, A3P, S10D, N13G, A15T, S23P, N28S, K29N, N34D, K35N, D36N, F42E, G44D, D48N, P49A, K50P, T54N, Y55I, T61S, L68V, M70V, R84Q, L89V, S91T, S102T, S104T, A107D, Y113L, S114A, T118P, P120S, I129L, T130S, Q135N, I143L, F146Y, Q147A, Q155P, R160K, L167F, A172V, I176V, S180T, R182K, M185V, R190A, A193Q, I201K, S206T, S210A, I213T, D216Q, T218I, V221N, K223N, Q238R, K248Q, L252I, P262S, Q267K, A276T, M278C, V294E, V301I, S303N, S306T, K307N, S311M, N314G, N318K, A323N, L328V, S331A, A335G, K342N, T352K, S362N, D364N, K366T, V377D, A378D, A382S, K393A, L419I, E421D, T424A, S425T, N427S, T428V, V429T, F430K, S432G, L435M, Y436H, F437Y, T440V, S451T, T452V, R459S, R460Y, E465T, S467T, V470Q, I475L, V477D, Q480T, Y484T, K485E, N487A, G505A, R506T, F508L, T519S, N531P, Y534F, S536A, N537D, N539S, L540P, K545V, R548S, T549V, Q552T, I560L, S563T, S567A, S569T, K570S, I574V, N596S, N597S, G599T, S603T, F604V, V606I, S608N, S610P, V612I, A626N, S633K, R636K, Q639L
GOase_wt *vs.* node 42	119	S2A, A3P, S10D, N13G, A15T, S23P, N28S, N34D, K35N, D36N, F42A, G44S, D48N, K50P, T54N, Y55I, T61S, M70V, L89V, S91T, S102T, S104T, S114A, T118P, P120S, I129L, T130S, Q135N, I143L, F146Y, Q155P, R160K, L167F, A172V, I176V, S180T, R182K, M185V, R190A, A193Q, I201R, S206T, S210A, I213T, D216Q, T218I, V221N, K223D, K248Q, Q267K, M278C, V301I, S303D, K307N, S311M, N314G, N318K, L328V, S331A, K342N, S362N, D364N, V377D, A378D, A382S, K393A, L419I, E421D, T424A, S425T, N427S, T428V, V429T, L435M, Y436H, F437Y, T440V, S451T, T452V, R459S, R460Y, E465T, V470Q, I475L, V477D, Y484V, K485E, N487A, G505A, R506T, T519S, N531S, Y534F, S536A, N537D, N539S, L540P, K545V, R548S, T549V, Q552S, I560L, S563T, S567A, S569T, K570S, N596S, N597S, G599T, S603T, F604A, V606I, S608N, S610P, V612I, A626N, S633K, R636K, Q639L
GOase_wt *vs.* node 45	148	S2A, A3P, S7N, S10D, N12A, N13G, A15K, A21E, Q22E, S23P, N28S, N34D, K35N, D36N, F42A, G44S, D48N, K50P, T54N, Y55I, I57V, K60G, T61S, M70V, G83A, L89V, S91T, S98D, S102T, S104T, F106Y, S114A, T118P, P120S, I129L, T130S, Q135N, I143L, F146Y, Q147K, S149N, S150T, Y151E, T152P, Q155A, P156A, R160K, L167F, A172V, A174G, I176V, E177D, T179L, S180T, R182K,

		M185V, R190A, A193N, G195E, I201R, S206T, S210A, I213D, S215T, D216Q, T218I, V221N, K223D, K248Q, S256A, Q267K, M278C, V301I, S303D, S306T, K307N, S311M, N314G, N318K, L328I, S331A, K342N, S362N, D364N, Q372K, V377D, A378D, A382S, K393A, D404S, L419I, E421D, T424A, S425T, N427S, T428V, V429T, L435M, Y436H, F437Y, T440V, S451T, T452V, R459S, R460Y, G461A, E465T, V470Q, I475L, V477D, Y484V, K485E, N487A, G505A, R506T, T519N, N531S, Y534F, S536R, N537D, N539S, L540P, T542A, K545V, R548S, T549V, Q552S, I560L, S563T, S567A, I568V, S569T, K570S, N596S, N597S, G599T, S603T, F604A, V606L, S608N, S610P, V612I, A613L, A626N, S633K, R636K, T638L, Q639L
GOase_wt *vs.* node 53	171	S2A, A3P, I5Y, G6N, S10D, N13D, A15T, A21E, S23P, N28S, K29N, N34D, K35N, D36N, T41S, F42E, G44D, A45Q, D48N, P49A, K50P, T54N, Y55I, M59L, T61S, N66S, L68V, S69A, M70V, R84Q, L89V, S91T, T94E, S102Y, S104T, F106W, A107D, Y113L, S114A, N115A, T118P, P120S, I129L, T130S, Q135N, I143L, V145I, F146Y, Q147A, S149N, Q155P, L158K, R160V, L167F, A172V, A173S, I176V, T179S, S180T, R182K, M185V, R190A, A193Q, S197T, I201K, S206T, S210A, I213T, D216Q, T218I, V221N, K223E, Q238R, K248T, L252I, P262S, D265P, Q267K, A276T, M278L, T285V, V294E, E296D, V301I, S303N, S306T, K307N, S311M, N314G, N318K, A323N, L328P, S331A, A335G, K342N, G343R, T352K, T359V, S360E, S362N, D364N, K366T, V377D, A378D, A382S, C383S, Y389F, K393A, Q406E, L419I, E421D, T424A, S425T, N427S, T428V, F430K, S432G, L435M, Y436H, F437Y, T440V, S451T, T452V, R459S, R460Y, E465N, D466E, S467E, V470Q, I475L, V477D, Q480T, Y484V, K485E, N487A, S490N, R506T, F508L, D517N, T519S, T520A, F523Y, N531P, Y534F, S536A, N537D, N539S, L540P, R548S, T549V, T551P, Q552S, I560L, S563T, S567A, S569T, K570S, I574V, N596S, N597S, G599T, S603T, F604V, V606I, S608N, S610P, V612I, A626N, S633K, R636K, Q639L

*The numbering of mutations shown in this is referred to GOase_wt. ASR nodes contain 641 amino acid residues in comparison to 639 found in GOase_wt; therefore, in ASR there are two insertions between positions 8/9, and between positions 293/294.

Table S4. Mutations of SarLacc variant compared to GOase wt.

Variant	Number of mutations	Mutations*	Approach/Notes
GOase wt *vs.* SarLacc	159 (+2 insertions)	S2A, A3P, S7N, **S10P**, N12D, N13G, A15K, A21E, Q22E, S23P, N28S, N34D, K35N, D36N, F42A, G44S, D48N, K50P, T54N, Y55I, I57V, K60G, T61S, Q63H, **M70V**, G83A, L89V, S91T, S98D, S104M, F106Y, S114A, T118P, P120S, I129L, T130S, Q135N, ***P136P (silent)***, I143L, F146Y, Q147K, S149N, S150T, Y151E, T152P, A153P, Q155A, P156A, R160K, L167F, A172V, A174G, I176V, E177D, T179L, S180T, R182K, M185V, R190A, A193Q, **G195E**, I201R, S206I, S210A, I213D, S215T, D216Q, T218I, V221N, K223D, K248Q, S256A, Q267K, A269P, M278L, **W290F**, V301I, S303D, S306T, K307N, S311L, N314G, N318K, A323N, L328I, **R330K**, S331A, K342N, S362N, D364N, Q372K, V377D, A378D, A381S, K393A, D404S, **Q406T**, L419I, E421D, T424A, S425T, N427S, T428V, V429T, L435M, Y436W, F437Y, T440V, S451T, T452V, R459S, R460Y, G461A, E465T, V470Q, I475L, V477D, Y484V, K485E, N487A, V492P, **V494A**, I498V, G505A, R506T, T519N, N531P, Y534F, **N535D**, S536R, N537D, N539S, L540P, T542A, K545V, R548S, T549V, Q552D, I560L, S563Q, S567P, I568V, S569T, K570S, Y576C, N596S, N597S, G599T, S603T, F604A, V606L, S609N, S610P, V612I, A613L, A626N, S633K, R636K, T638L, Q639L	Combinaton of SDR+PROSS (EvoPROSS) and ASR (node 45)

*The numbering of mutations shown in this table is referred to GOase wt. SarLacc 641 amino acid residues in comparison to 639 found in GOase wt; therefore, in SarLacc there are two insertions between positions 8/9, and between positions 293/294. Mutations in bold correspond to M-RQW.

Table S5. HPLC methods for reaction analysis of the substrate panel.*

**Substrate**	**Structure**	**Mobile Phases**	**Isocratic or Linear**	**t_R Substrate (min)**	**t_R Product (min)**
Benzylalcohol	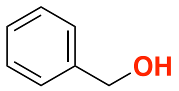	**C**: 0.1% TFA<br>**B**: ACN<br>**A**: H_2O<br>70(**B**):30(**B**):0.1(**C**)	Isocratic	2.20	2.59
Phenylethanol	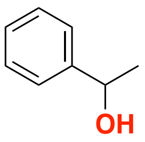	**B**: ACN<br>**A**: H_2O<br>50(**B**):50(**A**)	Isocratic	3.09	3.96
Phenylpropanol	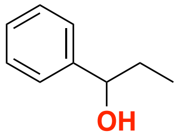	**B**: ACN<br>**A**: H_2O<br>50(**B**):50(**A**)	Isocratic	3.84	5.44
Phenylbutanol	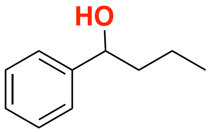	**B**: ACN<br>**A**: H_2O<br>70(**B**):30(**A**)	Isocratic	2.84	3.15
Phenylpentanol	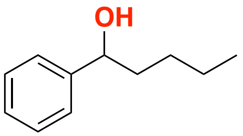	**B**: ACN<br>**A**: H_2O<br>70(**B**):30(**A**)	Isocratic	3.19	4.12

α-Tetralol	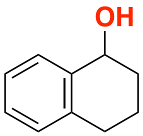	**B**: ACN<br>**A**: H_2O<br>70(**B**):30(**A**)	Isocratic	2.56	2.92
Diphenylmethanol	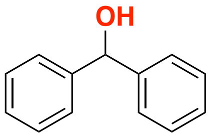	**B**: ACN<br>**A**: H_2O<br>70(**B**):30(**A**)	Isocratic	2.78	3.67

*HPLC Method specifications:

Column: VP-ODS C18 column (150 mm x 4.6 mm, 5 μm)
Injection volume: 10 μL
Flow rate and Elution time: 1 mL/min, 12 minutes.
Column temperature: 40 °C

Table S6. Primer list used in engineering campaigns.

Primer Name	5'→3' Sequence	Application
MKpGAOfw	GTATCGATTAAATAAGGAGGAATAAACAAGCTTatggcctcagcacctatcg	Random Mutagenesis
MKpGAOrv	CGTTTAAACTCAATGATGATGATGATGATGGTCGACTCActgagtaacgcgaatcgtg	
SDR-12Asn_Fw	cctcagcacctatcggaagcgccattcctcgcAACaactgggccgtcacttgcgacag	Site-Directed Recombination[a]
SDR-12Asp_Fw	cctcagcacctatcggaagcgccattcctcgcGACaactgggccgtcacttgcgacag	
SDR_102Leu-Rv	gtagagtcggcgaaccacataccTAAcgcaacagggctgccccagtttg	
SDR_102Ser_Rv	gtagagtcggcgaaccacataccTGAcgcaacagggctgccccagtttg	
SDR-102Ser_Fw	caaactggggcagccctgttgcgTCAggtatgtggttcgccgactctac	
SDR_102Leu-Fw	caaactggggcagccctgttgcgTTAggtatgtggttcgccgactctac	
SDR-413Asn_Rv	gttcaccgagggtgatgatgtgggcgTTGgttgtggcgtcagagtcCGTataatc	
SDR-413Asp_Rv	gttcaccgagggtgatgatgtgggcGTCggttgtggcgtcagagtcCGTataatc	
SDR-413Asn_Fw	gattatacggactctgacgccacaaccAACgcccacatcatcaccctcggtgaac	
SDR_413Asp_Fw	gattatacggactctgacgccacaaccGACgcccacatcatcaccctcggtgaac	
SDR-576Tyr_Rv	gtattaaccgtgtgtgtcgctgtaccATAgcgaatcaacgacgccttgctaatcg	
SDR_576Cys_Rv	gtattaaccgtgtgtgtcgctgtaccACAgcgaatcaacgacgccttgctaatcg	
SDR_576Tyr_Fw	cgattagcaaggcgtcgttgattcgcTATggtacagcgacacacacggttaatac	
SDR_576Cys_Fw	cgattagcaaggcgtcgttgattcgcTGTggtacagcgacacacacggttaatac	
SDR_FinalPCR_Rv	CCAAGCTGGAGACCGTTTAAACTC	

SDR_PCR2_Fw	GTATCGATTAAATAAGGAGGAATAAACAAGCTTatggcctcagcacctatcggaagc	
SDR_PCR2_Rv	gtagagtcggcgaaccacatac	
BB_pPiczA_Rv2	<u>gaatggcgcttccgataggtgctgaggc</u>AGCTTCAGCCTCTCTTTTCTCGAGAG	pPICZ-α cloning for *P. pastoris* expression[b]
bb_pPICZ_HTag_fw2	<u>gtggcttcgacgattcgcgttactcag</u>CATCATCATCATCATCATTGAGTTTGTAGCC	
RQW_pPICZa_fw2	<u>CTCTCGAGAAAAGAGAGGCTGAAGCT</u>gcctcagcacctatcggaagcgccattc	
RQW_PICZ_HTag_rv2	<u>GGCTACAAACTCAATGATGATGATGATGATG</u>ctgagtaacgcgaatcgtcgaagccac	
PROSS_PICZ_HTag_rv2	<u>GGCTACAAACTCAATGATGATGATGATGATG</u>ctgagtaacgcgaatcgtcttagccac	
bb_ASR45_fw	<u>GTCGCCAAGACGATCAAGGTTCTGTTG</u>catcatcatcatcatcattgagtttgtagcc	
bb_ASR45_rv	<u>CGGGCGTTACCAATGGGCGGAGCGGCA</u>gcttcagcctctcttttctcgagag	
ASR45_gene_fw	<u>CTCTCGAGAAAAGAGAGGCTGAAGCT</u>gccgctccgcccattggtaacgcccg	
ASR45_gene_rv	<u>GCTACAAACTCAATGATGATGATGATGATG</u>caacagaaccttgatcgtcttggcgac	
SarLacc_bb_Fw	<u>GAGGATGTCAGAATGCCATTTGCCTGAGAGATGC</u>aggc	
SarLacc_gene_Rv	<u>gcatctctcaggcaaatggcattctgacatcctc</u>TTGATTAGAATCTAGC	

[a]Base pairs highlighted in yellow are the mutations acquired from the directed evolution campaign and those highlighted in blue are the native counterparts present in M-RQW gene.
[b]Underlined portions of the primers represent the complementary overhangs to facilitate Gibson assembly.

Table S7. GOase constructions used in this study.

Name of the construct[a]	Expression host	Engineering approach
pTrcHis2B-(M-RQW)	*Escherichia coli*	Directed evolution
pTrcHis2B-(L3F3)	*Escherichia coli*	Directed evolution
pTrcHis2B-(node 39)	*Escherichia coli*	Ancestral sequence reconstruction
pTrcHis2B-(node 40)	*Escherichia coli*	Ancestral sequence reconstruction
pTrcHis2B-(node 41)	*Escherichia coli*	Ancestral sequence reconstruction
pTrcHis2B-(node 42)	*Escherichia coli*	Ancestral sequence reconstruction
pTrcHis2B-(node 45)	*Escherichia coli*	Ancestral sequence reconstruction
pTrcHis2B-(node 53)	*Escherichia coli*	Ancestral sequence reconstruction
pTrcHis2B-(PROSS1)	*Escherichia coli*	PROSS
pTrcHis2B-(PROSS3)	*Escherichia coli*	PROSS
pTrcHis2B-(PROSS4)	*Escherichia coli*	PROSS
pTrcHis2B-(EvoPROSS)	*Escherichia coli*	Site-Directed Recombination
pTrcHis2B-(SarLacc)	*Escherichia coli*	Hybrid approaches
pPICZα-(M-RQW)	*Pichia pastoris*	Directed evolution
pPICZα-(EvoPROSS)	*Pichia pastoris*	Site-Directed Recombination
pPICZα-(node 45)	*Pichia pastoris*	Ancestral sequence reconstruction
pPICZα-(SarLacc)	*Pichia pastoris*	Hybrid approaches

[a] GOase genes were cloned into pTrcHis2B containing a starting Met residue and a STOP codon at the end lacking His-tags, while for pPICZα-based constructs GOases were cloned after alpha factor pre-proleader and His-tags added at C-termini.

GOase sequences in this study

>GOase_wt

```
1      ATG GCC TCA GCA CCT ATC GGA AGC GCC ATT TCT CGC AAC AAC TGG   45
1       M   A   S   A   P   I   G   S   A   I   S   R   N   N   W   15

46     GCC GTC ACT TGC GAC AGT GCA CAG TCG GGA AAT GAA TGC AAC AAG   90
16      A   V   T   C   D   S   A   Q   S   G   N   E   C   N   K   30

91     GCC ATT GAT GGC AAC AAG GAT ACC TTT TGG CAC ACA TTC TAT GGC   135
31      A   I   D   G   N   K   D   T   F   W   H   T   F   Y   G   45

136    GCC AAC GGG GAT CCA AAG CCC CCT CAC ACA TAC ACG ATT GAC ATG   180
46      A   N   G   D   P   K   P   P   H   T   Y   T   I   D   M   60

181    AAG ACA ACT CAG AAC GTC AAC GGC TTG TCT ATG CTG CCT CGA CAG   225
61      K   T   T   Q   N   V   N   G   L   S   M   L   P   R   Q   75

226    GAT GGT AAC CAA AAC GGC TGG ATC GGT CGC CAT GAG GTT TAT CTA   270
76      D   G   N   Q   N   G   W   I   G   R   H   E   V   Y   L   90

271    AGC TCA GAT GGC ACA AAC TGG GGC AGC CCT GTT GCG TCA GGT AGT   315
91      S   S   D   G   T   N   W   G   S   P   V   A   S   G   S   105

316    TGG TTC GCC GAC TCT ACT ACA AAA TAC TCC AAC TTT GAA ACT CGC   360
106     W   F   A   D   S   T   T   K   Y   S   N   F   E   T   R   120

361    CCT GCT CGC TAT GTT CGT CTT GTC GCT ATC ACT GAA GCG AAT GGC   405
121     P   A   R   Y   V   R   L   V   A   I   T   E   A   N   G   135

406    CAG CCT TGG ACT AGC ATT GCA GAG ATC AAC GTC TTC CAA GCT AGT   450
136     Q   P   W   T   S   I   A   E   I   N   V   F   Q   A   S   150

451    TCT TAC ACA GCC CCC CAG CCT GGT CTT GGA CGC TGG GGT CCG ACT   495
151     S   Y   T   A   P   Q   P   G   L   G   R   W   G   P   T   165

496    ATT GAC TTA CCG ATT GTT CCT GCG GCT GCA GCA ATT GAA CCG ACA   540
166     I   D   L   P   I   V   P   A   A   A   A   I   E   P   T   180

541    TCG GGA CGA GTC CTT ATG TGG TCT TCA TAT CGC AAT GAT GCA TTT   585
181     S   G   R   V   L   M   W   S   S   Y   R   N   D   A   F   195

586    GGA GGA TCC CCT GGT GGT ATC ACT TTG ACG TCT TCC TGG GAT CCA   630
196     G   G   S   P   G   G   I   T   L   T   S   S   W   D   P   210

631    TCC ACT GGT ATT GTT TCC GAC CGC ACT GTG ACA GTC ACC AAG CAT   675
211     S   T   G   I   V   S   D   R   T   V   T   V   T   K   H   225

676    GAT ATG TTC TGC CCT GGT ATC TCC ATG GAT GGT AAC GGT CAG ATC   720
226     D   M   F   C   P   G   I   S   M   D   G   N   G   Q   I   240

721    GTA GTC ACA GGT GGC AAC GAT GCC AAG AAG ACC AGT TTG TAT GAT   765
241     V   V   T   G   G   N   D   A   K   K   T   S   L   Y   D   255

766    TCA TCT AGC GAT AGC TGG ATC CCG GGA CCT GAC ATG CAA GTG GCT   810
256     S   S   S   D   S   W   I   P   G   P   D   M   Q   V   A   270
```

811 CGT GGG TAT CAG TCA TCA GCT ACC ATG TCA GAC GGT CGT GTT TTT 855
271 R G Y Q S S A T M S D G R V F 285

856 ACC ATT GGA GGC TCC TGG AGC GGT GGC GTA TTT GAG AAG AAT GGC 900
286 T I G G S W S G G V F E K N G 300

901 GAA GTC TAT AGC CCA TCT TCA AAG ACA TGG ACG TCC CTA CCC AAT 945
301 E V Y S P S S K T W T S L P N 315

946 GCC AAG GTC AAC CCA ATG TTG ACG GCT GAC AAG CAA GGA TTG TAC 990
316 A K V N P M L T A D K Q G L Y 330

991 AGG TCA GAC AAC CAC GCG TGG CTC TTT GGA TGG AAG AAG GGT TCG 1035
331 R S D N H A W L F G W K K G S 345

1036 GTG TTC CAA GCG GGA CCT AGC ACA GCC ATG AAC TGG TAC TAT ACC 1080
346 V F Q A G P S T A M N W Y Y T 360

1081 AGT GGA AGT GGT GAT GTG AAG TCA GCC GGA AAA CGC CAG TCT AAC 1125
361 S G S G D V K S A G K R Q S N 375

1126 CGT GGT GTA GCC CCT GAT GCC ATG TGC GGA AAC GCT GTC ATG TAC 1170
376 R G V A P D A M C G N A V M Y 390

1171 GAC GCC GTT AAA GGA AAG ATC CTG ACC TTT GGC GGC TCC CCA GAT 1215
391 D A V K G K I L T F G G S P D 405

1216 TAT CAA GAC TCT GAC GCC ACA ACC AAC GCC CAC ATC ATC ACC CTC 1260
406 Y Q D S D A T T N A H I I T L 420

1261 GGT GAA CCC GGA ACA TCT CCC AAC ACT GTC TTT GCT AGC AAT GGG 1305
421 G E P G T S P N T V F A S N G 435

1306 TTG TAC TTT GCC CGA ACG TTT CAC ACC TCT GTT GTT CTT CCA GAC 1350
436 L Y F A R T F H T S V V L P D 450

1351 GGA AGC ACG TTT ATT ACA GGA GGC CAA CGA CGT GGA ATT CCG TTC 1395
451 G S T F I T G G Q R R G I P F 465

1396 GAG GAT TCA ACC CCG GTA TTT ACA CCT GAG ATC TAC GTC CCT GAA 1440
466 E D S T P V F T P E I Y V P E 480

1441 CAA GAC ACT TTC TAC AAG CAG AAC CCC AAC TCC ATT GTT CGC GTC 1485
481 Q D T F Y K Q N P N S I V R V 495

1486 TAC CAT AGC ATT TCC CTT TTG TTA CCT GAT GGC AGG GTA TTT AAC 1530
496 Y H S I S L L L P D G R V F N 510

1531 GGT GGT GGT GGT CTT TGT GGC GAT TGT ACC ACG AAT CAT TTC GAC 1575
511 G G G G L C G D C T T N H F D 525

1576 GCG CAA ATC TTT ACG CCA AAC TAT CTT TAC AAT AGC AAC GGC AAT 1620
526 A Q I F T P N Y L Y N S N G N 540

1621 CTC GCG ACA CGT CCC AAG ATT ACC AGA ACC TCT ACA CAG AGC GTC 1665
541 L A T R P K I T R T S T Q S V 555

1666 AAG GTC GGT GGC AGA ATT ACA ATC TCG ACG GAT TCT TCG ATT AGC 1710
556 K V G G R I T I S T D S S I S 570

1711 AAG GCG TCG TTG ATT CGC TAT GGT ACA GCG ACA CAC ACG GTT AAT 1755
571 K A S L I R Y G T A T H T V N 585

1756 ACT GAC CAG CGC CGC ATT CCC CTG ACT CTG ACA AAC AAT GGA GGA 1800
586 T D Q R R I P L T L T N N G G 600

1801 AAT AGC TAT TCT TTC CAA GTT CCT AGC GAC TCT GGT GTT GCT TTG 1845
601 N S Y S F Q V P S D S G V A L 615

1846 CCT GGC TAC TGG ATG TTG TTC GTG ATG AAC TCG GCC GGT GTT CCT 1890
616 P G Y W M L F V M N S A G V P 630

1891 AGT GTG GCT TCG ACG ATT CGC GTT ACT CAG TGA 1923
631 S V A S T I R V T Q *

>M-RQW

1 ATG GCC TCA GCA CCT ATC GGA AGC GCC ATT CCT CGC AAC AAC TGG 45
1 M A S A P I G S A I P R N N W 15

46 GCC GTC ACT TGC GAC AGT GCA CAG TCG GGA AAT GAA TGC AAC AAG 90
16 A V T C D S A Q S G N E C N K 30

91 GCC ATT GAT GGC AAC AAG GAT ACC TTT TGG CAC ACA TTC TAT GGC 135
31 A I D G N K D T F W H T F Y G 45

136 GCC AAC GGG GAT CCA AAG CCC CCT CAC ACA TAC ACG ATT GAC ATG 180
46 A N G D P K P P H T Y T I D M 60

181 AAG ACA ACT CAG AAC GTC AAC GGC TTG TCT GTG CTG CCT CGA CAG 225
61 K T T Q N V N G L S V L P R Q 75

226 GAT GGT AAC CAA AAC GGC TGG ATC GGT CGC CAT GAG GTT TAT CTA 270
76 D G N Q N G W I G R H E V Y L 90

271 AGC TCA GAT GGC ACA AAC TGG GGC AGC CCT GTT GCG TCA GGT AGT 315
91 S S D G T N W G S P V A S G S 105

316 TGG TTC GCC GAC TCT ACT ACA AAA TAC TCC AAC TTT GAA ACT CGC 360
106 W F A D S T T K Y S N F E T R 120

361 CCT GCT CGC TAT GTT CGT CTT GTC GCT ATC ACT GAA GCG AAT GGC 405
121 P A R Y V R L V A I T E A N G 135

406 CAG CCC TGG ACT AGC ATT GCA GAG ATC AAC GTC TTC CAA GCT AGT 450
136 Q P W T S I A E I N V F Q A S 150

451 TCT TAC ACA GCC CCC CAG CCT GGT CTT GGA CGC TGG GGT CCG ACT 495
151 S Y T A P Q P G L G R W G P T 165

496 ATT GAC TTA CCG ATT GTT CCT GCG GCT GCA GCA ATT GAA CCG ACA 540
166 I D L P I V P A A A A I E P T 180

541 TCG GGA CGA GTC CTT ATG TGG TCT TCA TAT CGC AAT GAT GCA TTT 585
181 S G R V L M W S S Y R N D A F 195

586 GAA GGA TCC CCT GGT GGT ATC ACT TTG ACG TCT TCC TGG GAT CCA 630
196 E G S P G G I T L T S S W D P 210

631 TCC ACT GGT ATT GTT TCC GAC CGC ACT GTG ACA GTC ACC AAG CAT 675
211 S T G I V S D R T V T V T K H 225

676 GAT ATG TTC TGC CCT GGT ATC TCC ATG GAT GGT AAC GGT CAG ATC 720
226 D M F C P G I S M D G N G Q I 240

721 GTA GTC ACA GGT GGC AAC GAT GCC AAG AAG ACC AGT TTG TAT GAT 765
241 V V T G G N D A K K T S L Y D 255

766 TCA TCT AGC GAT AGC TGG ATC CCG GGA CCT GAC ATG CAA GTG GCT 810
256 S S S D S W I P G P D M Q V A 270

811 CGT GGG TAT CAG TCA TCA GCT ACC ATG TCA GAC GGT CGT GTT TTT 855
271 R G Y Q S S A T M S D G R V F 285

856 ACC ATT GGA GGC TCC TTC AGC GGT GGC GTA TTT GAG AAG AAT GGC 900
286 T I G G S F S G G V F E K N G 300

901 GAA GTC TAT AGC CCA TCT TCA AAG ACA TGG ACG TCC CTA CCC AAT 945
301 E V Y S P S S K T W T S L P N 315

946 GCC AAG GTC AAC CCA ATG TTG ACG GCT GAC AAG CAA GGA TTG TAC 990
316 A K V N P M L T A D K Q G L Y 330

991 AAG TCA GAC AAC CAC GCG TGG CTC TTT GGA TGG AAG AAG GGT TCG 1035
331 K S D N H A W L F G W K K G S 345

1036 GTG TTC CAA GCG GGA CCT AGC ACA GCC ATG AAC TGG TAC TAT ACC 1080
346 V F Q A G P S T A M N W Y Y T 360

1081 AGT GGA AGT GGT GAT GTG AAG TCA GCC GGA AAA CGC CAG TCT AAC 1125
361 S G S G D V K S A G K R Q S N 375

1126 CGT GGT GTA GCC CCT GAT GCC ATG TGC GGA AAC GCT GTC ATG TAC 1170
376 R G V A P D A M C G N A V M Y 390

1171 GAC GCC GTT AAA GGA AAG ATC CTG ACC TTT GGC GGC TCC CCA GAT 1215
391 D A V K G K I L T F G G S P D 405

1216 TAT ACG GAC TCT GAC GCC ACA ACC AAC GCC CAC ATC ATC ACC CTC 1260
406 Y T D S D A T T N A H I I T L 420

1261 GGT GAA CCC GGA ACA TCT CCC AAC ACT GTC TTT GCT AGC AAT GGG 1305
421 G E P G T S P N T V F A S N G 435

1306 TTG TAC TTT GCC CGA ACG TTT CAC ACC TCT GTT GTT CTT CCA GAC 1350
436 L Y F A R T F H T S V V L P D 450

1351 GGA AGC ACG TTT ATT ACA GGA GGC CAA CGA CGT GGA ATT CCG TTC 1395
451 G S T F I T G G Q R R G I P F 465

1396 GAG GAT TCA ACC CCG GTA TTT ACA CCT GAG ATC TAC GTC CCT GAA 1440
466 E D S T P V F T P E I Y V P E 480

1441 CAA GAC ACT TTC TAC AAG CAG AAC CCC AAC TCC ATT GTT CGC GCC 1485
481 Q D T F Y K Q N P N S I V R A 495

1486 TAC CAT AGC ATT TCC CTT TTG TTA CCT GAT GGC AGG GTA TTT AAC 1530
496 Y H S I S L L L P D G R V F N 510

1531 GGT GGT GGT GGT CTT TGT GGC GAT TGT ACC ACG AAT CAT TTC GAC 1575
511 G G G G L C G D C T T N H F D 525

1576 GCG CAA ATC TTT ACG CCA AAC TAT CTT TAC GAT AGC AAC GGC AAT 1620
526 A Q I F T P N Y L Y D S N G N 540

1621 CTC GCG ACA CGT CCC AAG ATT ACC AGA ACC TCT ACA CAG AGC GTC 1665
541 L A T R P K I T R T S T Q S V 555

1666 AAG GTC GGT GGC AGA ATT ACA ATC TCG ACG GAT TCT TCG ATT AGC 1710
556 K V G G R I T I S T D S S I S 570

1711 AAG GCG TCG TTG ATT CGC TAT GGT ACA GCG ACA CAC ACG GTT AAT 1755
571 K A S L I R Y G T A T H T V N 585

1756 ACT GAC CAG CGC CGC ATT CCC CTG ACT CTG ACA AAC AAT GGA GGA 1800
586 T D Q R R I P L T L T N N G G 600

1801 AAT AGC TAT TCT TTC CAA GTT CCT AGC GAC TCT GGT GTT GCT TTG 1845
601 N S Y S F Q V P S D S G V A L 615

1846 CCT GGC TAC TGG ATG TTG TTC GTG ATG AAC TCG GCC GGT GTT CCT 1890
616 P G Y W M L F V M N S A G V P 630

1891 AGT GTG GCT TCG ACG ATT CGC GTT ACT CAG TGA 1923
631 S V A S T I R V T Q *

>L3F3

1 ATG GCC TCA GCA CCT ATC GGA AGC GCC ATT CCT CGC GAC AAC TGG 45
1 M A S A P I G S A I P R D N W 15

46 GCC GTC ACT TGC GAC AGT GCA CAG TCG GGA AAT GAA TGC AAC AAG 90
16 A V T C D S A Q S G N E C N K 30

91 GCC ATT GAT GGC AAC AAG GAT ACC TTT TGG CAC ACA TTC TAT GGC 135
31 A I D G N K D T F W H T F Y G 45

136 GCC AAC GGG GAT CCA AAG CCC CCC CAC ACA TAC ACG ATT GAC ATG 180
46 A N G D P K P P H T Y T I D M 60

181 AAG ACA ACT CAG AAC GTC AAC GGC TTG TCT GTG CTG CCT CGA CAG 225
61 K T T Q N V N G L S V L P R Q 75

226 GAT GGT AAC CAA AAC GGC TGG ATC GGT CGC CAT GAG GTT TAT CTA 270
76 D G N Q N G W I G R H E V Y L 90

271 AGC TCA GAT GGC ACA AAC TGG GGC AGC CCT GTT GCG TTA GGT AGT 315
91 S S D G T N W G S P V A L G S 105

316 TGG TTC GCC GAC TCT ACT ACA AAA TAC TCC AAC TTT GAA ACT CGC 360
106 W F A D S T T K Y S N F E T R 120

361 CCT GCT CGC TAT GTT CGT CTT GTC GCT ATC ACT GAA GCG AAT GGC 405
121 P A R Y V R L V A I T E A N G 135

406 CAG CCC TGG ACT AGC ATT GCA GAG ATC AAC GTC TTC CAA GCT AGT 450
136 Q P W T S I A E I N V F Q A S 150

451 TCT TAC ACA GCC CCC CAG CCT GGT CTT GGA CGC TGG GGT CCG ACT 495
151 S Y T A P Q P G L G R W G P T 165

496 ATT GAC TTA CCG ATT GTT CCT GCG GCT GCA GCA ATT GAA CCG ACA 540
166 I D L P I V P A A A A I E P T 180

541 TCG GGA CGA GTC CTT ATG TGG TCT TCA TAT CGC AAT GAT GCA TTT 585
181 S G R V L M W S S Y R N D A F 195

586 GAA GGA TCC CCT GGT GGT ATC ACT TTG ACG TCT TCC TGG GAT CCA 630
196 E G S P G G I T L T S S W D P 210

631 TCC ACT GGT ATT GTT TCC GAC CGC ACT GTG ACA GTC ACC AAG CAT 675
211 S T G I V S D R T V T V T K H 225

676 GAT ATG TTC TGC CCT GGT ATC TCC ATG GAT GGT AAC GGT CAG ATC 720
226 D M F C P G I S M D G N G Q I 240

721 GTA GTC ACA GGT GGC AAC GAT GCC AAG AAG ACC AGT TTG TAT GAT 765
241 V V T G G N D A K K T S L Y D 255

766 TCA TCT AGC GAT AGC TGG ATC CCG GGA CCT GAC ATG CAA GTG GCT 810
256 S S S D S W I P G P D M Q V A 270

811 CGT GGG TAT CAG TCA TCA GCT ACC ATG TCA GAC GGT CGT GTT TTT 855
271 R G Y Q S S A T M S D G R V F 285

856 ACC ATT GGA GGC TCC TTC AGC GGT GGC GTA TTT GAG AAG AAT GGC 900
286 T I G G S F S G G V F E K N G 300

901 GAA GTC TAT AGC CCA TCT TCA AAG ACA TGG ACG TCC CTA CCC AAT 945
301 E V Y S P S S K T W T S L P N 315

946 GCC AAG GTC AAC CCA ATG TTG ACG GCT GAC AAG CAA GGA TTG TAC 990
316 A K V N P M L T A D K Q G L Y 330

991 AAG TCA GAC AAC CAC GCG TGG CTC TTT GGA TGG AAG AAG GGT TCG 1035
331 K S D N H A W L F G W K K G S 345

1036 GTG TTC CAA GCG GGA CCT AGC ACA GCC ATG AAC TGG TAC TAT ACC 1080
346 V F Q A G P S T A M N W Y Y T 360

1081 AGT GGA AGT GGT GAT GTG AAG TCA GCC GGA AAA CGC CAG TCT AAC 1125
361 S G S G D V K S A G K R Q S N 375

1126 CGT GGT GTA GCC CCT GAT GCC ATG TGC GGA AAC GCT GTC ATG TAC 1170
376 R G V A P D A M C G N A V M Y 390

1171 GAC GCC GTT AAA GGA AAG ATC CTG ACC TTT GGC GGC TCC CCA GAT 1215
391 D A V K G K I L T F G G S P D 405

1216 TAT ACG GAC TCT GAC GCC ACA ACC GAC GCC CAC ATC ATC ACC CTC 1260
406 Y T D S D A T T D A H I I T L 420

1261 GGT GAA CCC GGA ACA TCT CCC AAC ACT GTC TTT GCT AGC AAT GGG 1305
421 G E P G T S P N T V F A S N G 435

1306 TTG TAC TTT GCC CGA ACG TTT CAC ACC TCT GTT GTT CTT CCA GAC 1350
436 L Y F A R T F H T S V V L P D 450

1351 GGA AGC ACG TTT ATT ACA GGA GGC CAA CGA CGT GGA ATT CCG TTC 1395
451 G S T F I T G G Q R R G I P F 465

1396 GAG GAT TCA ACC CCG GTA TTT ACA CCT GAG ATC TAC GTC CCT GAA 1440
466 E D S T P V F T P E I Y V P E 480

1441 CAA GAC ACT TTC TAC AAG CAG AAC CCC AAC TCC ATT GTT CGC GCC 1485
481 Q D T F Y K Q N P N S I V R A 495

1486 TAC CAT AGC ATT TCC CTT TTG TTA CCT GAT GGC AGG GTA TTT AAC 1530
496 Y H S I S L L L P D G R V F N 510

1531 GGT GGT GGT GGT CTT TGT GGC GAT TGT ACC ACG AAT CAT TTC GAC 1575
511 G G G G L C G D C T T N H F D 525

1576 GCG CAA ATC TTT ACG CCA AAC TAT CTT TAC GAT AGC AAC GGC AAT 1620
526 A Q I F T P N Y L Y D S N G N 540

1621 CTC GCG ACA CGT CCC AAG ATT ACC AGA ACC TCT ACA CAG AGC GTC 1665
541 L A T R P K I T R T S T Q S V 555

1666 AAG GTC GGT GGC AGA ATT ACA ATC TCG ACG GAT TCT TCG ATT AGC 1710
556 K V G G R I T I S T D S S I S 570

1711 AAG GCG TCG TTG ATT CGC TGT GGT ACA GCG ACA CAC ACG GTT AAT 1755
571 K A S L I R C G T A T H T V N 585

1756 ACT GAC CAG CGC CGC ATT CCC CTG ACT CTG ACA AAC AAT GGA GGA 1800
586 T D Q R R I P L T L T N N G G 600

```
1801  AAT AGT TAT TCT TTC CAA GTT CCT AGC GAC TCT GGT GTT GCT TTG  1845
601    N   S   Y   S   F   Q   V   P   S   D   S   G   V   A   L   615

1846  CCT GGC TAC TGG ATG TTG TTC GTG ATG AAC TCG GCC GGT GTT CCT  1890
616    P   G   Y   W   M   L   F   V   M   N   S   A   G   V   P   630

1891  AGT GTG GCT TCG ACG ATT CGC GTT ACT CAG TGA  1923
631    S   V   A   S   T   I   R   V   T   Q   *
```

>PROSS_1

```
1     ATG GCC TCA GCA CCT ATC GGA AGC GCC ATT CCT CGC AAC AAC TGG  45
1      M   A   S   A   P   I   G   S   A   I   P   R   N   N   W   15

46    GCC GTC ACT TGC GAC AGT GCA CAG TCG GGA AAT GAA TGC AAC AAG  90
16     A   V   T   C   D   S   A   Q   S   G   N   E   C   N   K   30

91    GCC ATT GAT GGC AAC AAG GAT ACC TTT TGG CAC ACA TTC TAT GGC  135
31     A   I   D   G   N   K   D   T   F   W   H   T   F   Y   G   45

136   GCC AAC GGG GAT CCA AAG CCC CCT CAC ACA TAC ACG ATT GAC ATG  180
46     A   N   G   D   P   K   P   P   H   T   Y   T   I   D   M   60


181   AAG ACA ACT CAT AAC GTC AAC GGC TTG TCT GTG CTG CCT CGA CAG  225
61     K   T   T   H   N   V   N   G   L   S   V   L   P   R   Q   75

226   GAT GGT AAC CAA AAC GGC TGG ATC GGT CGC CAT GAG GTT TAT CTA  270
76     D   G   N   Q   N   G   W   I   G   R   H   E   V   Y   L   90

271   AGC TCA GAT GGC ACA AAC TGG GGC AGC CCT GTT GCG TCA GGT ATG  315
91     S   S   D   G   T   N   W   G   S   P   V   A   S   G   M   105

316   TGG TTC GCC GAC TCT ACT ACA AAA TAC TCC AAC TTT GAA ACT CGC  360
106    W   F   A   D   S   T   T   K   Y   S   N   F   E   T   R   120

361   CCT GCT CGC TAT GTT CGT CTT GTC GCT ATC ACT GAA GCG AAT GGC  405
121    P   A   R   Y   V   R   L   V   A   I   T   E   A   N   G   135

406   CAG CCC TGG ACT AGC ATT GCA GAG ATC AAC GTC TTC CAA GCT AGT  450
136    Q   P   W   T   S   I   A   E   I   N   V   F   Q   A   S   150

451   TCT TAC ACA GCC CCC CAG CCT GGT CTT GGA CGC TGG GGT CCG ACT  495
151    S   Y   T   A   P   Q   P   G   L   G   R   W   G   P   T   165

496   ATT GAC TTA CCG ATT GTT CCT GCG GCT GCA GCA ATT GAA CCG ACA  540
166    I   D   L   P   I   V   P   A   A   A   A   I   E   P   T   180

541   TCG GGA CGA GTC CTT ATG TGG TCT TCA TAT CGC AAT GAT GCA TTT  585
181    S   G   R   V   L   M   W   S   S   Y   R   N   D   A   F   195

586   GAA GGA TCC CCT GGT GGT ATC ACT TTG ACG TCT ATC TGG GAT CCA  630
196    E   G   S   P   G   G   I   T   L   T   S   I   W   D   P   210
```

631 TCC ACT GGT ATT GTT TCC GAC CGC ACT GTG ACA AAT ACC AAG CAT 675
211 S T G I V S D R T V T N T K H 225

676 GAT ATG TTC TGC CCT GGT ATC TCC ATG GAT GGT AAC GGT CAG ATC 720
226 D M F C P G I S M D G N G Q I 240

721 GTA GTC ACA GGT GGC AAC GAT GCC AAG AAG ACC AGT TTG TAT GAT 765
241 V V T G G N D A K K T S L Y D 255

766 TCA TCT AGC GAT AGC TGG ATC CCG GGA CCT GAC ATG CAA GTG CCA 810
256 S S S D S W I P G P D M Q V P 270

811 CGT GGG TAT CAG TCA TCA GCT ACC ATG TCA GAC GGT CGT GTT TTT 855
271 R G Y Q S S A T M S D G R V F 285

856 ACC ATT GGA GGC TCC TTC AGC GGT GGC GTA TTT GAG AAG AAT GGC 900
286 T I G G S F S G G V F E K N G 300

901 GAA GTC TAT GAT CCA TCT TCA AAG ACA TGG ACG TCC CTA CCC AAT 945
301 E V Y D P S S K T W T S L P N 315

946 GCC AAG GTC AAC CCA ATG TTG ACG GCT GAC AAG CAA GGA TTG TAC 990
316 A K V N P M L T A D K Q G L Y 330

991 AAG TCA GAC AAC CAC GCG TGG CTC TTT GGA TGG AAG AAC GGT TCG 1035
331 K S D N H A W L F G W K N G S 345

1036 GTG TTC CAA GCG GGA CCT AGC ACA GCC ATG AAC TGG TAC TAT ACC 1080
346 V F Q A G P S T A M N W Y Y T 360

1081 AGT GGA AGT GGT GAT GTG AAG TCA GCC GGA AAA CGC CAG TCT AAC 1125
361 S G S G D V K S A G K R Q S N 375

1126 CGT GGT GTA GCC CCT GAT GCC ATG TGC GGA AAC GCT GTC ATG TAC 1170
376 R G V A P D A M C G N A V M Y 390

1171 GAC GCC GTT AAA GGA AAG ATC CTG ACC TTT GGC GGC TCC CCA GAT 1215
391 D A V K G K I L T F G G S P D 405

1216 TAT ACG GAC TCT GAC GCC ACA ACC AAC GCC CAC ATC ATC ACC CTC 1260
406 Y T D S D A T T N A H I I T L 420

1261 GGT GAA CCC GGA ACA TCT CCC AAC GTA GTC TTT GCT AGC AAT GGG 1305
421 G E P G T S P N V V F A S N G 435

1306 TTG TAC TTT GCC CGA ACG TTT CAC ACC TCT GTT GTT CTT CCA GAC 1350
436 L Y F A R T F H T S V V L P D 450

1351 GGA AGC GTT TTT ATT ACA GGA GGC CAA CGA CGT GGA ATT CCG TTC 1395
451 G S V F I T G G Q R R G I P F 465

1396 GAG GAT TCA ACC CCG GTA TTT ACA CCT GAG ATC TAC GTC CCT GAA 1440
466 E D S T P V F T P E I Y V P E 480

1441 CAA GAC ACT TTC TAC AAG CAG AAC CCC AAC TCC ATT CCT CGC GCC 1485
481 Q D T F Y K Q N P N S I P R A 495

1486 TAC CAT AGC ATT TCC CTT TTG TTA CCT GAT GGC AGG GTA TTT AAC 1530
496 Y H S I S L L L P D G R V F N 510

1531 GGT GGT GGT GGT CTT TGT GGC GAT TGT ACC ACG AAT CAT TTC GAC 1575
511 G G G G L C G D C T T N H F D 525

1576 GCG CAA ATC TTT ACG CCA CCT TAT CTT TAC GAT AGC AAC GGC AAT 1620
526 A Q I F T P P Y L Y D S N G N 540

1621 CTC GCG ACA CGT CCC AAG ATT ACC AGT ACC TCT ACA CAG AGC GTC 1665
541 L A T R P K I T S T S T Q S V 555

1666 AAG GTC GGT GGC AGA ATT ACA ATC CAA ACG GAT TCT TCG ATT AGC 1710
556 K V G G R I T I Q T D S S I S 570

1711 AAG GCG TCG TTG ATT CGC TAT GGT ACA GCG ACA CAC ACG GTT AAT 1755
571 K A S L I R Y G T A T H T V N 585

1756 ACT GAC CAG CGC CGC ATT CCC CTG ACT CTG ACA AAC AAT GGA GGA 1800
586 T D Q R R I P L T L T N N G G 600

1801 AAT AGC TAT TCT TTC CAA GTT CCT AGC GAC TCT GGT GTT GCT TTG 1845
601 N S Y S F Q V P S D S G V A L 615

1846 CCT GGC TAC TGG ATG TTG TTC GTG ATG AAC TCG GCC GGT GTT CCT 1890
616 P G Y W M L F V M N S A G V P 630

1891 AGT GTG GCT TCG ACG ATT CGC GTT ACT CAG TGA 1923
631 S V A S T I R V T Q *

>PROSS_3

1 ATG GCC TCA GCA CCT ATC GGA AGC GCC ATT CCT CGC AAC AAC TGG 45
1 M A S A P I G S A I P R N N W 15

46 GCC GTC ACT TGC GAC AGT GCA CAG TCG GGA AAT GAA TGC AAC AAG 90
16 A V T C D S A Q S G N E C N K 30

91 GCC ATT GAT GGC AAC AAG GAT ACC TTT TGG CAC ACA TTC TAT GGC 135
31 A I D G N K D T F W H T F Y G 45

136 GCC AAC GGG GAT CCA AAG CCC CCT CAC ACA TAC ACG ATT GAC ATG 180
46 A N G D P K P P H T Y T I D M 60

181 GGA ACA ACT CAT AAC GTC AAC GGC TTG TCT GTG CTG CCT CGA CAG 225
61 G T T H N V N G L S V L P R Q 75

226 GAT GGT AAC CAA AAC GGC TGG ATC GGT CGC CAT GAG GTT TAT CTA 270
76 D G N Q N G W I G R H E V Y L 90

```
271   AGC TCA GAT GGC ACA AAC TGG GGC AGC CCT GTT GCG TCA GGT ATG   315
91     S   S   D   G   T   N   W   G   S   P   V   A   S   G   M    105

316   TGG TTC GCC GAC TCT ACT ACA AAA TAC TCC AAC TTT GAA CCA CGC   360
106    W   F   A   D   S   T   T   K   Y   S   N   F   E   P   R    120

361   CCT GCT CGC TAT GTT CGT CTT GTC GCT ATC ACT GAA GCG AAT GGC   405
121    P   A   R   Y   V   R   L   V   A   I   T   E   A   N   G    135

406   CAG CCC TGG ACT AGC ATT GCA GAG ATC AAC GTC TTC CAA GCT AGT   450
136    Q   P   W   T   S   I   A   E   I   N   V   F   Q   A   S    150

451   TCT TAC ACA CCG CCC CAG CCT GGT CTT GGA CGC TGG GGT CCG ACT   495
151    S   Y   T   P   P   Q   P   G   L   G   R   W   G   P   T    165

496   ATT GAC TTA CCG ATT GTT CCT GCG GCT GCA GCA ATT GAA CCG ACA   540
166    I   D   L   P   I   V   P   A   A   A   A   I   E   P   T    180

541   TCG GGA CGA GTC CTT ATG TGG TCT TCA TAT CGC AAT GAT CAG TTT   585
181    S   G   R   V   L   M   W   S   S   Y   R   N   D   Q   F    195

586   GAA GGA TCC CCT GGT GGT ATC ACT TTG ACG TCT ATC TGG GAT CCA   630
196    E   G   S   P   G   G   I   T   L   T   S   I   W   D   P    210

631   TCC ACT GGT ATT GTT TCC GAC CGC ACT GTG ACA AAT ACC AAG CAT   675
211    S   T   G   I   V   S   D   R   T   V   T   N   T   K   H    225

676   GAT ATG TTC TGC CCT GGT ATC TCC ATG GAT GGT AAC GGT CAG ATC   720
226    D   M   F   C   P   G   I   S   M   D   G   N   G   Q   I    240

721   GTA GTC ACA GGT GGC AAC GAT GCC AAG AAG ACC AGT TTG TAT GAT   765
241    V   V   T   G   G   N   D   A   K   K   T   S   L   Y   D    255

766   TCA TCT AGC GAT AGC TGG ATC CCG GGA CCT GAC ATG CAA GTG CCA   810
256    S   S   S   D   S   W   I   P   G   P   D   M   Q   V   P    270

811   CGT GGG TAT CAG TCA TCA GCT ACC CTA TCA GAC GGT CGT GTT TTT   855
271    R   G   Y   Q   S   S   A   T   L   S   D   G   R   V   F    285

856   ACC ATT GGA GGC TCC TTC AGC GGT GGC GTA TTT GAG AAG AAT GGC   900
286    T   I   G   G   S   F   S   G   G   V   F   E   K   N   G    300

901   GAA GTC TAT GAT CCA TCT TCA AAC ACA TGG ACG CTA CTA CCC AAT   945
301    E   V   Y   D   P   S   S   N   T   W   T   L   L   P   N    315

946   GCC AAG GTC AAC CCA ATG TTG ACG AAC GAC AAG CAA GGA TTG TAC   990
316    A   K   V   N   P   M   L   T   N   D   K   Q   G   L   Y    330

991   AAG TCA GAC AAC CAC GCG TGG CTC TTT GGA TGG AAG AAC GGT TCG   1035
331    K   S   D   N   H   A   W   L   F   G   W   K   N   G   S    345

1036  GTG TTC CAA GCG GGA CCT AGC ACA GCC ATG AAC TGG TAC TAT ACC   1080
346    V   F   Q   A   G   P   S   T   A   M   N   W   Y   Y   T    360
```

```
1081  AGT GGA AGT GGT GAT GTG AAG TCA GCC GGA AAA CGC CAG TCT AAC  1125
361    S   G   S   G   D   V   K   S   A   G   K   R   Q   S   N   375

1126  CGT GGT GTA GAC CCT GAT GCC ATG TGC GGA AAC GCT GTC ATG TAC  1170
376    R   G   V   D   P   D   A   M   C   G   N   A   V   M   Y   390

1171  GAC GCC GTT AAA GGA AAG ATC CTG ACC TTT GGC GGC TCC CCA GAT  1215
391    D   A   V   K   G   K   I   L   T   F   G   G   S   P   D   405

1216  TAT ACG GAC TCT GAC GCC ACA ACC AAC GCC CAC ATC ATC ACC CTC  1260
406    Y   T   D   S   D   A   T   T   N   A   H   I   I   T   L   420

1261  GGT GAA CCC GGA ACA TCT CCC AAC GTA GTC TTT GCT AGC AAT GGG  1305
421    G   E   P   G   T   S   P   N   V   V   F   A   S   N   G   435

1306  TTG TGG TTT GCC CGA ACG TTT CAC ACC TCT GTT GTT CTT CCA GAC  1350
436    L   W   F   A   R   T   F   H   T   S   V   V   L   P   D   450

1351  GGA AGC GTT TTT ATT ACA GGA GGC CAA CGA CGT GGA ATT CCG TTC  1395
451    G   S   V   F   I   T   G   G   Q   R   R   G   I   P   F   465

1396  GAG GAT TCA ACC CCG GTA TTT ACA CCT GAG ATC TAC GAC CCT GAA  1440
466    E   D   S   T   P   V   F   T   P   E   I   Y   D   P   E   480

1441  CAA GAC ACT TTC TAC AAG CAG AAC CCC AAC TCC ATT CCT CGC GCC  1485
481    Q   D   T   F   Y   K   Q   N   P   N   S   I   P   R   A   495

1486  TAC CAT AGC GTA TCC CTT TTG TTA CCT GAT GGC AGG GTA TTT AAC  1530
496    Y   H   S   V   S   L   L   L   P   D   G   R   V   F   N   510

1531  GGT GGT GGT GGT CTT TGT GGC GAT TGT ACC ACG AAT CAT TTC GAC  1575
511    G   G   G   G   L   C   G   D   C   T   T   N   H   F   D   525

1576  GCG CAA ATC TTT ACG CCA CCT TAT CTT TTC GAT AGC AAC GGC AAT  1620
526    A   Q   I   F   T   P   P   Y   L   F   D   S   N   G   N   540

1621  CTC GCG ACA CGT CCC AAG ATT ACC AGT ACC TCT ACA GAT AGC GTC  1665
541    L   A   T   R   P   K   I   T   S   T   S   T   D   S   V   555

1666  AAG GTC GGT GGC AGA ATT ACA ATC CAA ACG GAT TCT TCG ATT AGC  1710
556    K   V   G   G   R   I   T   I   Q   T   D   S   S   I   S   570

1711  AAG GCG TCG TTG ATT CGC TAT GGT ACA GCG ACA CAC ACG GTT AAT  1755
571    K   A   S   L   I   R   Y   G   T   A   T   H   T   V   N   585

1756  ACT GAC CAG CGC CGC ATT CCC CTG ACT CTG ACA AAC AAT GGA GGA  1800
586    T   D   Q   R   R   I   P   L   T   L   T   N   N   G   G   600

1801  AAT AGC TAT TCT TTC CAA GTT CCT AGC GAC TCT GGT GTT GCT TTG  1845
601    N   S   Y   S   F   Q   V   P   S   D   S   G   V   A   L   615

1846  CCT GGC TAC TGG ATG TTG TTC GTG ATG AAC TCG GCC GGT GTT CCT  1890
616    P   G   Y   W   M   L   F   V   M   N   S   A   G   V   P   630
```

```
1891  AGT GTG GCT AAG ACG ATT CGC GTT ACT CAG TGA   1923
631    S   V   A   K   T   I   R   V   T   Q   *
```

>PROSS_4

```
1      ATG GCC TCA GCA CCT ATC GGA AGC GCC ATT CCT CGC AAC AAC TGG   45
1       M   A   S   A   P   I   G   S   A   I   P   R   N   N   W   15

46     ACC GTC ACT TGC GAC AGT GCA CAG TCG GGA AAT GAA TGC AAC AAG   90
16      T   V   T   C   D   S   A   Q   S   G   N   E   C   N   K   30

91     GCC ATT GAT GGC AAC AAG GAT ACC TTT TGG CAC ACA TTC TAT GGC   135
31      A   I   D   G   N   K   D   T   F   W   H   T   F   Y   G   45

136    GCC AAC GGG GAT CCA AAG CCC CCT CAC ACA TAC ACG ATT GAC ATG   180
46      A   N   G   D   P   K   P   P   H   T   Y   T   I   D   M   60

181    GGA ACA ACT CAT AAC GTC AAC GGC TTG TCT GTG CTG CCT CGA CAG   225
61      G   T   T   H   N   V   N   G   L   S   V   L   P   R   Q   75

226    GAT GGT AAC CAA AAC GGC TGG ATC GGT CGC CAT GAG GTT TAT CTA   270
76      D   G   N   Q   N   G   W   I   G   R   H   E   V   Y   L   90

271    AGC ACG GAT GGC ACA AAC TGG GGC AGC CCT GTT GCG TCA GGT ATG   315
91      S   T   D   G   T   N   W   G   S   P   V   A   S   G   M   105

316    TGG TAC GCC GAC TCT ACT ACA AAA TAC TCC AAC TTT GAA CCA CGC   360
106     W   Y   A   D   S   T   T   K   Y   S   N   F   E   P   R   120

361    CCT GCT CGC TAT GTT CGT CTT GTC GCT ATC ACT GAA GCG AAT GGC   405
121     P   A   R   Y   V   R   L   V   A   I   T   E   A   N   G   135

406    CAG CCC TGG ACT AGC ATT GCA GAG ATC AAC GTC TTC CAA GCT AGT   450
136     Q   P   W   T   S   I   A   E   I   N   V   F   Q   A   S   150

451    TCT TAC ACA CCG CCC CAG CCT GGT CTT GGA CGC TGG GGT CCG ACT   495
151     S   Y   T   P   P   Q   P   G   L   G   R   W   G   P   T   165

496    ATT GAC TTA CCG ATT GTT CCT GCG GCT GCA GCA ATT GAA CCG ACA   540
166     I   D   L   P   I   V   P   A   A   A   A   I   E   P   T   180

541    TCG GGA CGA GTC CTT ATG TGG TCT TCA TAT CGC AAT GAT CAG TTT   585
181     S   G   R   V   L   M   W   S   S   Y   R   N   D   Q   F   195

586    GAA GGA TCC CCT GGT GGT ATC ACT TTG ACG GCC ATC TGG GAT CCA   630
196     E   G   S   P   G   G   I   T   L   T   A   I   W   D   P   210

631    TCC ACT GGT ATT GTT TCC GAC CGC ACT GTG ACA AAT ACC CAT CAT   675
211     S   T   G   I   V   S   D   R   T   V   T   N   T   H   H   225

676    GAT ATG TTC TGC CCT GGT ATC TCC ATG GAT GGT AAC GGT CAG ATC   720
226     D   M   F   C   P   G   I   S   M   D   G   N   G   Q   I   240
```

721 GTA GTC ACA GGT GGC AAC GAT GCC AAG AAG ACC AGT TTG TAT GAT 765
241 V V T G G N D A K K T S L Y D 255

766 CCT TCT AGC GAT AGC TGG ATC CCG GGA CCT GAC ATG CAA GTG CCA 810
256 P S S D S W I P G P D M Q V P 270

811 CGT GGG TAT CAG TCA TCA GCT ACC CTA TCA GAC GGT CGT GTT TTT 855
271 R G Y Q S S A T L S D G R V F 285

856 ACC ATT GGA GGC TCC TTC AGC GGT GGC GTA TTT GAG AAG AAT GGC 900
286 T I G G S F S G G V F E K N G 300

901 GAA GTC TAT GAT CCA TCT ACA AAC ACA TGG ACG CTA CTA CCC AAT 945
301 E V Y D P S T N T W T L L P N 315

946 GCC AAG GTC AAC CCA ATG TTG ACG AAC GAC AAG CAA GGA TTG TAC 990
316 A K V N P M L T N D K Q G L Y 330

991 AAG TCA GAC AAC CAC GCG TGG CTC TTT GGA TGG AAG AAC GGT TCG 1035
331 K S D N H A W L F G W K N G S 345

1036 GTG TTC CAA GCG GGA CCT AGC ACA GCC ATG AAC TGG TAC TAT ACC 1080
346 V F Q A G P S T A M N W Y Y T 360

1081 AGT GGA AGT GGT GAT GTG AAG TCA GCC GGA AAA CGC CAG TCT AAC 1125
361 S G S G D V K S A G K R Q S N 375

1126 CGT GGT GTA GAC CCT GAT GCC ATG TGC GGA AAC GCT GTC ATG TAC 1170
376 R G V D P D A M C G N A V M Y 390

1171 GAC GCC GTT AAA GGA AAG ATC CTG ACC TTT GGC GGC TCC CCA CAT 1215
391 D A V K G K I L T F G G S P H 405

1216 TAT ACG GAC TCT GAC GCC ACA ACC AAC GCC CAC ATC ATC ACC CTC 1260
406 Y T D S D A T T N A H I I T L 420

1261 GGT GAA CCC GGA ACA TCT CCC AAC GTA GTC TTT GCT AGC AAT GGG 1305
421 G E P G T S P N V V F A S N G 435

1306 TTG TGG TTT GCC CGA ACG TTT CAC ACC TCT GTT GTT CTT CCA GAC 1350
436 L W F A R T F H T S V V L P D 450

1351 GGA AGC GTT TTT ATT ACA GGA GGC CAA CGA CGT GGA ATT CCG TTC 1395
451 G S V F I T G G Q R R G I P F 465

1396 GAG GAT TCA ACC CCG GTA TTT ACA CCT GAG ATC TAC GAC CCT GAA 1440
466 E D S T P V F T P E I Y D P E 480

1441 CAA GAC ACT TTC TAC AAG CAG AAC CCC AAC TCC ATT CCT CGC GCC 1485
481 Q D T F Y K Q N P N S I P R A 495

1486 TAC CAT AGC GTA TCC CTT TTG TTA CCT GAT GGC AGG GTA TTT AAC 1530
496 Y H S V S L L L P D G R V F N 510

1531 GGT GGT GGT GGT CTT TGT GGC GAT TGT ACC ACG AAT CAT TTC GAC 1575
511 G G G G L C G D C T T N H F D 525

1576 GCG CAA ATC TTT ACG CCA CCT TAT CTT TTC GAT AGC AAC GGC AAT 1620
526 A Q I F T P P Y L F D S N G N 540

1621 CCA GCG ACA CGT CCC AAG ATT ACC AGT GTC TCT ACA GAT AGC GTC 1665
541 P A T R P K I T S V S T D S V 555

1666 AAG GTC GGT GGC AGA ATT ACA ATC CAA ACG GAT TCT TCG ATT AAG 1710
556 K V G G R I T I Q T D S S I K 570

1711 AAG GCG TCG TTG ATT CGC TAT GGT ACA GCG ACA CAC ACG GTT AAT 1755
571 K A S L I R Y G T A T H T V N 585

1756 ACT GAC CAG CGC CGC ATT CCC CTG ACT CTG ACA AAC AAT GGA GGA 1800
586 T D Q R R I P L T L T N N G G 600

1801 AAT AGC TAT TCT TTC CAA GTT CCT AGC GAC TCT GGT GTT GCT TTG 1845
601 N S Y S F Q V P S D S G V A L 615

1846 CCT GGC TAC TGG ATG TTG TTC GTG ATG AAC TCG GCC GGT GTT CCT 1890
616 P G Y W M L F V M N S A G V P 630

1891 AGT GTG GCT AAG ACG ATT CGC GTT ACT CAG TGA 1923
631 S V A K T I R V T Q *

>EvoPROSS

1 ATG GCC TCA GCA CCT ATC GGA AGC GCC ATT CCT CGC GAC AAC TGG 45
1 M A S A P I G S A I P R D N W 15

46 GCC GTC ACT TGC GAC AGT GCA CAG TCG GGA AAT GAA TGC AAC AAG 90
16 A V T C D S A Q S G N E C N K 30

91 GCC ATT GAT GGC AAC AAG GAT ACC TTT TGG CAC ACA TTC TAT GGC 135
31 A I D G N K D T F W H T F Y G 45

136 GCC AAC GGG GAT CCA AAG CCC CCC CAC ACA TAC ACG ATT GAC ATG 180
46 A N G D P K P P H T Y T I D M 60

181 GGA ACA ACT CAT AAC GTC AAC GGC TTG TCT GTG CTG CCT CGA CAG 225
61 G T T H N V N G L S V L P R Q 75

226 GAT GGT AAC CAA AAC GGC TGG ATC GGT CGC CAT GAG GTT TAT CTA 270
76 D G N Q N G W I G R H E V Y L 90

271 AGC TCA GAT GGC ACA AAC TGG GGC AGC CCT GTT GCG TCA GGT ATG 315
91 S S D G T N W G S P V A S G M 105

316 TGG TTC GCC GAC TCT ACT ACA AAA TAC TCC AAC TTT GAA CCA CGC 360
106 W F A D S T T K Y S N F E P R 120

```
361   CCT GCT CGC TAT GTT CGT CTT GTC GCT ATC ACT GAA GCG AAT GGC   405
121    P   A   R   Y   V   R   L   V   A   I   T   E   A   N   G    135

406   CAG CCC TGG ACT AGC ATT GCA GAG ATC AAC GTC TTC CAA GCT AGT   450
136    Q   P   W   T   S   I   A   E   I   N   V   F   Q   A   S    150

451   TCT TAC ACA CCG CCC CAG CCT GGT CTT GGA CGC TGG GGT CCG ACT   495
151    S   Y   T   P   P   Q   P   G   L   G   R   W   G   P   T    165

496   ATT GAC TTA CCG ATT GTT CCT GCG GCT GCA GCA ATT GAA CCG ACA   540
166    I   D   L   P   I   V   P   A   A   A   A   I   E   P   T    180

541   TCG GGA CGA GTC CTT ATG TGG TCT TCA TAT CGC AAT GAT CAG TTT   585
181    S   G   R   V   L   M   W   S   S   Y   R   N   D   Q   F    195

586   GAA GGA TCC CCT GGT GGT ATC ACT TTG ACG TCT ATC TGG GAT CCA   630
196    E   G   S   P   G   G   I   T   L   T   S   I   W   D   P    210

631   TCC ACT GGT ATT GTT TCC GAC CGC ACT GTG ACA AAT ACC AAG CAT   675
211    S   T   G   I   V   S   D   R   T   V   T   N   T   K   H    225

676   GAT ATG TTC TGC CCT GGT ATC TCC ATG GAT GGT AAC GGT CAG ATC   720
226    D   M   F   C   P   G   I   S   M   D   G   N   G   Q   I    240

721   GTA GTC ACA GGT GGC AAC GAT GCC AAG AAG ACC AGT TTG TAT GAT   765
241    V   V   T   G   G   N   D   A   K   K   T   S   L   Y   D    255

766   TCA TCT AGC GAT AGC TGG ATC CCG GGA CCT GAC ATG CAA GTG CCA   810
256    S   S   S   D   S   W   I   P   G   P   D   M   Q   V   P    270

811   CGT GGG TAT CAG TCA TCA GCT ACC CTA TCA GAC GGT CGT GTT TTT   855
271    R   G   Y   Q   S   S   A   T   L   S   D   G   R   V   F    285

856   ACC ATT GGA GGC TCC TTC AGC GGT GGC GTA TTT GAG AAG AAT GGC   900
286    T   I   G   G   S   F   S   G   G   V   F   E   K   N   G    300

901   GAA GTC TAT GAT CCA TCT TCA AAC ACA TGG ACG CTA CTA CCC AAT   945
301    E   V   Y   D   P   S   S   N   T   W   T   L   L   P   N    315

946   GCC AAG GTC AAC CCA ATG TTG ACG AAC GAC AAG CAA GGA TTG TAC   990
316    A   K   V   N   P   M   L   T   N   D   K   Q   G   L   Y    330

991   AAG TCA GAC AAC CAC GCG TGG CTC TTT GGA TGG AAG AAC GGT TCG   1035
331    K   S   D   N   H   A   W   L   F   G   W   K   N   G   S    345

1036  GTG TTC CAA GCG GGA CCT AGC ACA GCC ATG AAC TGG TAC TAT ACC   1080
346    V   F   Q   A   G   P   S   T   A   M   N   W   Y   Y   T    360

1081  AGT GGA AGT GGT GAT GTG AAG TCA GCC GGA AAA CGC CAG TCT AAC   1125
361    S   G   S   G   D   V   K   S   A   G   K   R   Q   S   N    375

1126  CGT GGT GTA GAC CCT GAT GCC ATG TGC GGA AAC GCT GTC ATG TAC   1170
376    R   G   V   D   P   D   A   M   C   G   N   A   V   M   Y    390
```

1171 GAC GCC GTT AAA GGA AAG ATC CTG ACC TTT GGC GGC TCC CCA GAT 1215
391 D A V K G K I L T F G G S P D 405

1216 TAT ACG GAC TCT GAC GCC ACA ACC AAC GCC CAC ATC ATC ACC CTC 1260
406 Y T D S D A T T N A H I I T L 420

1261 GGT GAA CCC GGA ACA TCT CCC AAC GTA GTC TTT GCT AGC AAT GGG 1305
421 G E P G T S P N V V F A S N G 435

1306 TTG TGG TTT GCC CGA ACG TTT CAC ACC TCT GTT GTT CTT CCA GAC 1350
436 L W F A R T F H T S V V L P D 450

1351 GGA AGC GTT TTT ATT ACA GGA GGC CAA CGA CGT GGA ATT CCG TTC 1395
451 G S V F I T G G Q R R G I P F 465

1396 GAG GAT TCA ACC CCG GTA TTT ACA CCT GAG ATC TAC GAC CCT GAA 1440
466 E D S T P V F T P E I Y D P E 480

1441 CAA GAC ACT TTC TAC AAG CAG AAC CCC AAC TCC ATT CCT CGC GCC 1485
481 Q D T F Y K Q N P N S I P R A 495

1486 TAC CAT AGC GTA TCC CTT TTG TTA CCT GAT GGC AGG GTA TTT AAC 1530
496 Y H S V S L L L P D G R V F N 510

1531 GGT GGT GGT GGT CTT TGT GGC GAT TGT ACC ACG AAT CAT TTC GAC 1575
511 G G G G L C G D C T T N H F D 525

1576 GCG CAA ATC TTT ACG CCA CCT TAT CTT TTC GAT AGC AAC GGC AAT 1620
526 A Q I F T P P Y L F D S N G N 540

1621 CTC GCG ACA CGT CCC AAG ATT ACC AGT ACC TCT ACA GAT AGC GTC 1665
541 L A T R P K I T S T S T D S V 555

1666 AAG GTC GGT GGC AGA ATT ACA ATC CAA ACG GAT TCT CCG ATT AGC 1710
556 K V G G R I T I Q T D S P I S 570

1711 AAG GCG TCG TTG ATT CGC TGT GGT ACA GCG ACA CAC ACG GTT AAT 1755
571 K A S L I R C G T A T H T V N 585

1756 ACT GAC CAG CGC CGC ATT CCC CTG ACT CTG ACA AAC AAT GGA GGA 1800
586 T D Q R R I P L T L T N N G G 600

1801 AAT AGC TAT TCT TTC CAA GTT CCT AGC GAC TCT GGT GTT GCT TTG 1845
601 N S Y S F Q V P S D S G V A L 615

1846 CCT GGC TAC TGG ATG TTG TTC GTG ATG AAC TCG GCC GGT GTT CCT 1890
616 P G Y W M L F V M N S A G V P 630

1891 AGT GTG GCT AAG ACG ATT CGC GTT ACT CAG TGA 1923
631 S V A K T I R V T Q *

>Node_39

```
1      ATG GCA GCG GCA CCT AGT GGA TCA GTC GCA ATC GAT CGC AAC GGA   45
1       M   A   A   A   P   S   G   S   V   A   I   D   R   N   G    15

46     TGG ACA GCG ACC TGT GAT TCT GCC CAG CCC GGG AAT GAA TGC TCA   90
16      W   T   A   T   C   D   S   A   Q   P   G   N   E   C   S    30

91     AAT GCA TTA GAC GGA GAC ACG AAC ACT TTC TGG CAT ACT GAA TAT   135
31      N   A   L   D   G   D   T   N   T   F   W   H   T   E   Y    45

136    GAT CCT ACC GGC AAC GCG CCA TTG CCT CAT AAC ATT ACC ATC GAT   180
46      D   P   T   G   N   A   P   L   P   H   N   I   T   I   D    60

181    ATG AAA GCG ACG TAC AAC GTA AAT GGA GTC AGC TAC TTG CCG CGT   225
61      M   K   A   T   Y   N   V   N   G   V   S   Y   L   P   R    75

226    CAG GAT GGC AAT AGT AAC GGA AAC ATC GGC CAG CAC GAG GTG TAT   270
76      Q   D   G   N   S   N   G   N   I   G   Q   H   E   V   Y    90

271    GTT TCA ACG GAT GGC ACT AAT TGG GGC TCA CCT GTA GCC ACG GGC   315
91      V   S   T   D   G   T   N   W   G   S   P   V   A   T   G    105

316    ACG TGG TTG AAT GAT AGC ACC ACG AAA ACC GCG AAT TTT GAA CCA   360
106     T   W   L   N   D   S   T   T   K   T   A   N   F   E   P    120

361    CGC TCT GCC CGC TAT GTA CGT TTG GTG GCG TTA TCT GAA GCG AAC   405
121     R   S   A   R   Y   V   R   L   V   A   L   S   E   A   N    135

406    GGT AAC CCC TGG ACA AGC GCC GCC GAG ATT AAC ATC TAT ACC GCT   450
136     G   N   P   W   T   S   A   A   E   I   N   I   Y   T   A    150

451    TCC AGT TAT ATC GCC CCA CCA TCG GGC AAA GGG AAA TGG GGA CCC   495
151     S   S   Y   I   A   P   P   S   G   K   G   K   W   G   P    165

496    ACG ATT GAT TTT CCA ATC GTA CCT GTG GCA GCT GCC GTC GAG CCG   540
166     T   I   D   F   P   I   V   P   V   A   A   A   V   E   P    180

541    ACA ACG GGT AAA GTG TTG GTC TGG TCT TCA TAC GCG CCC GAC CAG   585
181     T   T   G   K   V   L   V   W   S   S   Y   A   P   D   Q    195

586    TTC GGC GGG TCC CCG GGC GGA CAG ACG CTT ACC GCC ACA TAT GAC   630
196     F   G   G   S   P   G   G   Q   T   L   T   A   T   Y   D    210

631    CCT GCT ACG GGC ACG GTT TCC CAA CGC ATT GTC ACT AAT ACA AAT   675
211     P   A   T   G   T   V   S   Q   R   I   V   T   N   T   N    225

676    CAC GAT ATG TTT TGT CCA GGA ATC AGT ATG GAT TTT AAC GGT CGC   720
226     H   D   M   F   C   P   G   I   S   M   D   F   N   G   R    240

721    ATT GTC GTT ACC GGC GGG AAT GAC GCG CAA AAG ACC TCG ATC TAT   765
241     I   V   V   T   G   G   N   D   A   Q   K   T   S   I   Y    255
```

766 GAT CCC TCG TCT GAT AGC TGG ACA AGC GGG CCA GAT ATG AAA ATT 810
256 D P S S D S W T S G P D M K I 270

811 GCG CGT GGA TAT CAA AGC TCG ACT ACT TGT AGC GAT GGT CGC ATT 855
271 A R G Y Q S S T T C S D G R I 285

856 TTC ACG ATC GGC GGA TCA TGG TCA GGT GGG GAG GAG GGT GGT AAG 900
286 F T I G G S W S G G E E G G K 300

901 AAT GGG GAA ATT TAC AAT CCT TCC TCT AAC ACC TGG ACT CTT CTG 945
301 N G E I Y N P S S N T W T L L 315

946 CCA GGC GCC AAG GTG GCG CCT ATG TTG ACT AAC GAT CGC CAG GGC 990
316 P G A K V A P M L T N D R Q G 330

991 GTT TAC CGC GCG GAT AAC CAC GGT TGG TTA TTT GGT TGG AAG AAT 1035
331 V Y R A D N H G W L F G W K N 345

1036 GGC TCA GTT TTT CAG GCA GGT CCA AGC AAA GCG ATG AAT TGG TAC 1080
346 G S V F Q A G P S K A M N W Y 360

1081 AAC ACC TCA GGG AAC GGC TCC GTC ACA GGG GCT GGA AAA CGC CAA 1125
361 N T S G N G S V T G A G K R Q 375

1126 TCT AAC CGT GGC GAT GAC GCT GAC AGC ATG TGT GGT AAC GCT GTA 1170
376 S N R G D D A D S M C G N A V 390

1171 ATG TAC GAC GCC GTG GCG GGC AAA ATC TTA ACC GTA GGA GGT TCA 1215
391 M Y D A V A G K I L T V G G S 405

1216 CCC AAC TAT CAG GAC GCT GAC GCT ACC ACA AAC GCC CAT ATC ATT 1260
406 P N Y Q D A D A T T N A H I I 420

1261 ACT ATC GGA AAT CCG GGG GCT ACC CCG ACA GTC ACA AAG GCA GCG 1305
421 T I G N P G A T P T V T K A A 435

1306 AGT GGT ATG TCT TAT GCT CGC ATC TTT CAC AAC TCG GTC GTT CTT 1350
436 S G M S Y A R I F H N S V V L 450

1351 CCA GAC GGG ACT GTC TTT ATT ACA GGC GGA CAG AGC TAC GGT GAG 1395
451 P D G T V F I T G G Q S Y G E 465

1396 CCC TTC ACC GAC ACG ACC GCA GAG TTT ACG CCT GAG TTA TGG AAC 1440
466 P F T D T T A E F T P E L W N 480

1441 CCA GCC ACT AAC ACC TTC ACC CAG ATG GCT CCT AAC TCA ATT CCT 1485
481 P A T N T F T Q M A P N S I P 495

1486 CGT ACG TAC CAT TCC ATT GCG TTG CTG CTT CCG GAT GCA ACC GTG 1530
496 R T Y H S I A L L L P D A T V 510

1531 TTG AAT GGG GGC GGC GGA TTA TGT GGC AAT TGT TCG ACC AAC CAC 1575
511 L N G G G G L C G N C S T N H 525

```
1576  TTC GAT GCA CAA ATC TTT ACC CCG CCC TAC TTG TTC AAT GCC GAT   1620
526    F   D   A   Q   I   F   T   P   P   Y   L   F   N   A   D    540

1621  GGG AGT CGC GCT ACC CGT CCT GTT ATC ACT TCA GTC TCT ACG ACC   1665
541    G   S   R   A   T   R   P   V   I   T   S   V   S   T   T    555

1666  AGC GTA AAA GTG GGT GGA ACT TTG ACG ATC ACT ACA AAC TCG GCC   1710
556    S   V   K   V   G   G   T   L   T   I   T   T   N   S   A    570

1711  GTG ACA TCA GCT AGC TTA GTA CGC TAT GGT ACC GCG ACT CAT ACG   1755
571    V   T   S   A   S   L   V   R   Y   G   T   A   T   H   T    585

1756  GTC AAC ACT GAC CAA CGT CGC ATT CCG TTA ACG CTT ACA TCC AGT   1800
586    V   N   T   D   Q   R   R   I   P   L   T   L   T   S   S    600

1801  GGA ACG AAT AGT TAT ACG GTG ACA ATT CCC TCG GAC CCT GGA GTG   1845
601    G   T   N   S   Y   T   V   T   I   P   S   D   P   G   V    615

1846  GCA CTT CCG GGC TAT TGG ATG TTA TTC GTC ATG AAT TCA AAC GGG   1890
616    A   L   P   G   Y   W   M   L   F   V   M   N   S   N   G    630

1891  GTT CCG TCA ATT GCT AAG ACC ATT AAG GTA ACT CCA TGA   1929
631    V   P   S   I   A   K   T   I   K   V   T   P   *
```

>Node_40

```
1     ATG GCG GCC GCG CCG TCT GGT TCG GTG GCG ATC GAC CGT AAT GGT   45
1      M   A   A   A   P   S   G   S   V   A   I   D   R   N   G    15

46    TGG ACA GCG ACG TGC GAT AGT GCA CAG CCG GGT AAC GAG TGC TCG   90
16     W   T   A   T   C   D   S   A   Q   P   G   N   E   C   S    30

91    AAT GCG ATT GAC GGT GAT ACG AAT ACG TTC TGG CAC ACT GAA TAT   135
31     N   A   I   D   G   D   T   N   T   F   W   H   T   E   Y    45

136   GAT CCG ACG GGA AAT GCT CCT TTA CCC CAT AAC ATC ACG ATT GAC   180
46     D   P   T   G   N   A   P   L   P   H   N   I   T   I   D    60

181   ATG AAG GCG ACA TAC AAT GTC AAC GGC GTT TCG TAC CTT CCT CGC   225
61     M   K   A   T   Y   N   V   N   G   V   S   Y   L   P   R    75

226   CAA GAC GGA AAT AGC AAC GGG AAT ATT GGT CAG CAC GAA GTT TAT   270
76     Q   D   G   N   S   N   G   N   I   G   Q   H   E   V   Y    90

271   GTC AGC ACA GAC GGT ACG AAT TGG GGA TCA CCT GTG GCC ACG GGT   315
91     V   S   T   D   G   T   N   W   G   S   P   V   A   T   G    105

316   ACA TGG CTT GAC GAC TCG ACA ACG AAG ACG GCA AAT TTC GAG CCT   360
106    T   W   L   D   D   S   T   T   K   T   A   N   F   E   P    120

361   CGT TCA GCA CGT TAC GTG CGC CTT GTG GCT CTG AGC GAA GCA AAT   405
121    R   S   A   R   Y   V   R   L   V   A   L   S   E   A   N    135
```

406 GGC AAC CCT TGG ACG TCA GCT GCA GAG ATC AAT ATC TAC ACG GCA 450
136 G N P W T S A A E I N I Y T A 150

451 TCA TCC TAT ATT GCA CCG CCA TCA GGG TTA GGA AAG TGG GGA CCT 495
151 S S Y I A P P S G L G K W G P 165

496 ACC ATC GAC TTT CCT ATT GTT CCC GTG GCT GCT GCT GTC GAG CCC 540
166 T I D F P I V P V A A A V E P 180

541 ACG ACC GGA AAA GTT CTG GTA TGG TCT AGT TAC GCG CCA GAC CAA 585
181 T T G K V L V W S S Y A P D Q 195

586 TTC GGG GGG AGT CCA GGG GGG CAA ACA TTA ACA GCG ACC TAT GAC 630
196 F G G S P G G Q T L T A T Y D 210

631 CCT GCT ACG GGC ACA GTT AGC CAA CGC ATT GTT ACC AAC ACT AAT 675
211 P A T G T V S Q R I V T N T N 225

676 CAT GAC ATG TTT TGC CCG GGG ATT TCG ATG GAC TTT AAT GGC CGT 720
226 H D M F C P G I S M D F N G R 240

721 ATT GTC GTT ACC GGT GGA AAT GAC GCA CAG AAG ACG TCT ATT TAT 765
241 I V V T G G N D A Q K T S I Y 255

766 GAC CCA AGC TCG GAT AGC TGG ACA TCA GGA CCT GAC ATG AAA ATT 810
256 D P S S D S W T S G P D M K I 270

811 GCT CGC GGT TAC CAA TCT TCT ACA ACG TGC TCA GAT GGA CGT ATT 855
271 A R G Y Q S S T T C S D G R I 285

856 TTC ACC ATC GGG GGT TCC TGG TCA GGG GGA GAA GAG GGC GGT AAG 900
286 F T I G G S W S G G E E G G K 300

901 AAT GGA GAG ATT TAT AAC CCC AGC TCA AAT ACC TGG ACA CTG CTT 945
301 N G E I Y N P S S N T W T L L 315

946 CCA GGT GCT AAA GTA GCC CCG ATG TTG ACG AAT GAC CGT CAG GGA 990
316 P G A K V A P M L T N D R Q G 330

991 GTG TAC CGC GCC GAT AAT CAC GGC TGG CTT TTC GGG TGG AAA AAC 1035
331 V Y R A D N H G W L F G W K N 345

1036 GGC AGT GTT TTC CAA GCG GGA CCG TCA AAG GCT ATG AAT TGG TAC 1080
346 G S V F Q A G P S K A M N W Y 360

1081 AAC ACG AGC GGA AAC GGT TCG GTT ACC GGG GCT GGC AAA CGC CAG 1125
361 N T S G N G S V T G A G K R Q 375

1126 TCG AAT CGC GGT GAT GAT GCA GAC TCG ATG TGT GGA AAC GCA GTG 1170
376 S N R G D D A D S M C G N A V 390

1171 ATG TAC GAT GCT GTC GCC GGG AAG ATC CTT ACA GTG GGT GGG TCC 1215
391 M Y D A V A G K I L T V G G S 405

1216 CCC AAC TAT CAG GAT GCC GAC GCC ACA ACT AAT GCA CAC ATT ATC 1260
406 P N Y Q D A D A T T N A H I I 420

1261 ACA ATT GGA AAT CCG GGG GCG ACG CCT TCT GTT ACA AAA GCA GCC 1305
421 T I G N P G A T P S V T K A A 435

1306 TCC GGT ATG TCA TAC GCC CGT ATC TTT CAC AAT AGT GTG GTT CTT 1350
436 S G M S Y A R I F H N S V V L 450

1351 CCC GAT GGA ACA GTG TTC ATT ACG GGA GGG CAA TCC TAC GGT GAG 1395
451 P D G T V F I T G G Q S Y G E 465

1396 CCT TTC ACA GAC ACT ACG GCA GAA TTT ACT CCG GAG TTA TGG AAC 1440
466 P F T D T T A E F T P E L W N 480

1441 CCT GCT ACA AAT ACA TTT ACG CAG ATG GCA CCA AAT AGC ATT CCG 1485
481 P A T N T F T Q M A P N S I P 495

1486 CGC ACA TAT CAT AGT ATT GCT TTG TTG TTA CCG GAC GCC ACG GTA 1530
496 R T Y H S I A L L L P D A T V 510

1531 CTG AAC GGA GGC GGA GGG CTG TGC GGT AAT TGC TCG ACG AAT CAT 1575
511 L N G G G G L C G N C S T N H 525

1576 TTC GAC GCG CAG ATT TTT ACC CCC CCG TAT TTA TTT AAC GCG GAC 1620
526 F D A Q I F T P P Y L F N A D 540

1621 GGG TCT CCG GCA ACT CGT CCG GTA ATC ACC TCG GTA TCC ACA ACT 1665
541 G S P A T R P V I T S V S T T 555

1666 TCT GTC AAG GTC GGG GGA ACT TTA ACA ATC ACA ACC AAC TCT GCG 1710
556 S V K V G G T L T I T T N S A 570

1711 GTC ACA TCC GCT TCT CTG GTT CGT TAT GGA ACG GCA ACG CAC ACC 1755
571 V T S A S L V R Y G T A T H T 585

1756 GTT AAC ACA GAC CAA CGT CGC ATC CCG CTG ACG TTA ACC TCT AGT 1800
586 V N T D Q R R I P L T L T S S 600

1801 GGT ACT AAC AGT TAT ACT GTG ACC ATT CCT AAC GAC CCT GGC GTC 1845
601 G T N S Y T V T I P N D P G V 615

1846 GCG CTG CCT GGG TAT TGG ATG CTT TTT GTT ATG AAC AGT AAT GGA 1890
616 A L P G Y W M L F V M N S N G 630

1891 GTA CCT AGC ATC GCG AAG ACG ATC AAG GTC ACC CCG TGA 1929
631 V P S I A K T I K V T P *

>Node_41

1 ATG GCA GCT CCG CCT ATC GGT TCG GCG CGC ATC GAT CGT AAT GGT 45
1 M A A P P I G S A R I D R N G 15

46 TGG ACA GTA ACC TGT GAT TCT GCG CAG CCT GGG AAT GAA TGC AGT 90
16 W T V T C D S A Q P G N E C S 30

91 AAT GCC ATT GAT GGG GAC AAT AAC ACG TTC TGG CAT ACG GAA TAT 135
31 N A I D G D N N T F W H T E Y 45

136 GAC GCA AAT GGT AAC GCA CCA CCT CCG CAT AAT ATC ACT ATC GAT 180
46 D A N G N A P P P H N I T I D 60

181 ATG AAG TCG ACT CAG AAT GTA AAT GGG GTC AGT GTC TTA CCT CGT 225
61 M K S T Q N V N G V S V L P R 75

226 CAG GAT GGC AAC CAA AAC GGA TGG ATC GGG CAG CAC GAA GTG TAT 270
76 Q D G N Q N G W I G Q H E V Y 90

271 GTG TCA ACT GAC GGA ACA AAC TGG GGA TCA CCC GTG GCT ACA GGA 315
91 V S T D G T N W G S P V A T G 105

316 ACA TGG TTT GAT GAC TCA ACG ACC AAA CTG GCA AAT TTT GAG CCT 360
106 T W F D D S T T K L A N F E P 120

361 CGT AGT GCA CGT TAC GTG CGC TTA GTC GCG TTG AGT GAA GCT AAT 405
121 R S A R Y V R L V A L S E A N 135

406 GGC AAT CCG TGG ACA TCC ATC GCG GAA CTT AAT GTG TAC GCA GCG 450
136 G N P W T S I A E L N V Y A A 150

451 TCC TCC TAT ACG GCA CCT CCA CCG GGT TTA GGG AAG TGG GGG CCT 495
151 S S Y T A P P P G L G K W G P 165

496 ACA ATT GAT TTC CCG ATC GTA CCG GTA GCA GCT GCC GTT GAG CCC 540
166 T I D F P I V P V A A A V E P 180

541 ACC ACT GGA AAA GTG TTG GTT TGG TCG TCG TAC GCA AAT GAC CAG 585
181 T T G K V L V W S S Y A N D Q 195

586 TTT GGT GGT TCC CCG GGA GGG AAG ACT TTA ACC TCC ACC TGG GAC 630
196 F G G S P G G K T L T S T W D 210

631 CCC GCG ACC GGT ACG GTT TCC CAG CGT ATC GTT ACA AAC ACG AAC 675
211 P A T G T V S Q R I V T N T N 225

676 CAT GAC ATG TTC TGC CCA GGC ATC TCC ATG GAT GGA AAC GGC CGC 720
226 H D M F C P G I S M D G N G R 240

721 ATC GTT GTT ACT GGC GGA AAC GAC GCC CAA AAA ACG TCA ATT TAC 765
241 I V V T G G N D A Q K T S I Y 255

766 GAC TCT TCT TCG GAT TCG TGG ATT TCA GGC CCC GAT ATG AAA GTG 810
256 D S S S D S W I S G P D M K V 270

811 GCA CGT GGG TAC CAA AGC AGT ACT ACC TGT TCC GAT GGG CGC GTT 855
271 A R G Y Q S S T T C S D G R V 285

856 TTT ACT ATT GGC GGT AGT TGG TCT GGG GGA GAA GAG TTT GAA AAA 900
286 F T I G G S W S G G E E F E K 300

901 AAC GGC GAA ATT TAC AAC CCT TCT ACT AAT ACC TGG ACT ATG CTG 945
301 N G E I Y N P S T N T W T M L 315

946 CCG GGA GCC AAA GTG AAA CCG ATG CTT ACT AAT GAT AAA CAG GGT 990
316 P G A K V K P M L T N D K Q G 330

991 GTG TAT CGC GCA GAC AAT CAT GGA TGG CTG TTC GGG TGG AAA AAC 1035
331 V Y R A D N H G W L F G W K N 345

1036 GGT TCG GTT TTC CAA GCA GGT CCC AGC AAA GCT ATG AAC TGG TAT 1080
346 G S V F Q A G P S K A M N W Y 360

1081 TAT ACG TCT GGC AAT GGA AAT GTA ACT TCA GCT GGC AAA CGC CAG 1125
361 Y T S G N G N V T S A G K R Q 375

1126 TCG AAT CGT GGT GAT GAC CCC GAT TCA ATG TGT GGA AAC GCC GTA 1170
376 S N R G D D P D S M C G N A V 390

1171 ATG TAC GAC GCT GTG GCC GGC AAA ATC TTG ACT TTT GGA GGT TCT 1215
391 M Y D A V A G K I L T F G G S 405

1216 CCT GAT TAC CAA GAT TCA GAC GCG ACT ACT AAC GCG CAT ATT ATC 1260
406 P D Y Q D S D A T T N A H I I 420

1261 ACC ATC GGA GAC CCT GGT GCC ACC CCC AGC GTG ACC AAG GCT GGT 1305
421 T I G D P G A T P S V T K A G 435

1306 AAC GGC ATG CAC TAC GCC CGT GTT TTT CAT ACC AGT GTG GTT CTT 1350
436 N G M H Y A R V F H T S V V L 450

1351 CCG GAC GGT ACC GTT TTT ATC ACT GGC GGT CAG AGC TAC GGA ATC 1395
451 P D G T V F I T G G Q S Y G I 465

1396 CCT TTT ACC GAT ACG ACA CCG CAA TTC ACG CCA GAA CTG TAC GAT 1440
466 P F T D T T P Q F T P E L Y D 480

1441 CCC GAA ACA GAT ACA TTT ACA GAG CAA GCC CCT AAT TCC ATT GTT 1485
481 P E T D T F T E Q A P N S I V 495

1486 CGC GTC TAC CAT TCC ATT TCA CTT CTT CTT CCG GAC GCA ACT GTC 1530
496 R V Y H S I S L L L P D A T V 510

1531 CTT AAT GGC GGG GGT GGG TTA TGT GGA GAT TGT TCG ACT AAT CAC 1575
511 L N G G G G L C G D C S T N H 525

1576 TTT GAT GCC CAG ATT TTT ACC CCC CCA TAT TTA TTT AAT GCC GAC 1620
526 F D A Q I F T P P Y L F N A D 540

1621 GGA TCG CCG GCT ACG CGT CCA GTG ATC ACT AGC GTC TCG ACC ACG 1665
541 G S P A T R P V I T S V S T T 555

1666 TCT GTA AAG GTA GGA GGC CGT TTA ACT ATC ACA ACA GAT TCT GCT 1710
556 S V K V G G R L T I T T D S A 570

1711 ATC ACG AGT GCT TCC TTA GTG CGT TAC GGC ACA GCG ACG CAC ACA 1755
571 I T S A S L V R Y G T A T H T 585

1756 GTT AAT ACT GAT CAG CGT CGC ATC CCA TTG ACC CTT ACG AGC AGT 1800
586 V N T D Q R R I P L T L T S S 600

1801 GGC ACC AAC AGC TAT ACT GTG CAG ATT CCC AAT GAC CCT GGG ATC 1845
601 G T N S Y T V Q I P N D P G I 615

1846 GCG CTG CCC GGA TAT TGG ATG CTG TTT GTT ATG AAT TCT AAC GGG 1890
616 A L P G Y W M L F V M N S N G 630

1891 GTC CCA TCT GTA GCT AAG ACA ATT AAA GTT ACC TTG TGA 1929
631 V P S V A K T I K V T L *

>Node_42

1 ATG GCC GCG CCC CCA ATT GGT AGT GCG CGT ATT GAC CGT AAT GGC 45
1 M A A P P I G S A R I D R N G 15

46 TGG ACT GTT ACG TGT GAC TCC GCT CAG CCC GGC AAT GAG TGC TCG 90
16 W T V T C D S A Q P G N E C S 30

91 AAA GCT ATT GAT GGC GAC AAC AAC ACC TTC TGG CAT ACC GCT TAT 135
31 K A I D G D N N T F W H T A Y 45

136 TCT GCT AAC GGA AAC CCA CCA CCC CCC CAC AAT ATT ACT ATC GAC 180
46 S A N G N P P P P H N I T I D 60

181 ATG AAA TCG ACC CAG AAC GTC AAC GGA TTA AGC GTT CTG CCT CGT 225
61 M K S T Q N V N G L S V L P R 75

226 CAA GAC GGC AAC CAG AAC GGA TGG ATC GGA CGT CAC GAA GTC TAT 270
76 Q D G N Q N G W I G R H E V Y 90

271 GTT AGT ACG GAT GGA ACG AAC TGG GGT AGT CCG GTT GCA ACG GGT 315
91 V S T D G T N W G S P V A T G 105

316 ACG TGG TTT GCT GAC TCC ACC ACG AAG TAT GCT AAC TTT GAG CCA 360
106 T W F A D S T T K Y A N F E P 120

361 CGC TCA GCA CGC TAC GTT CGT CTT GTA GCA TTG TCC GAG GCA AAT 405
121 R S A R Y V R L V A L S E A N 135

406 GGA AAC CCC TGG ACC AGC ATC GCG GAG TTA AAC GTG TAC CAG GCA 450
136 G N P W T S I A E L N V Y Q A 150

451 AGC AGT TAT ACT GCG CCA CCC CCT GGT CTT GGA AAG TGG GGT CCG 495
151 S S Y T A P P P G L G K W G P 165

496 ACC ATT GAC TTC CCA ATC GTG CCG GTT GCA GCT GCC GTG GAG CCC 540
166 T I D F P I V P V A A A V E P 180

541 ACG ACC GGA AAA GTT CTG GTT TGG AGT TCG TAC GCT AAT GAC CAA 585
181 T T G K V L V W S S Y A N D Q 195

586 TTT GGC GGG TCA CCT GGA GGT CGT ACG CTG ACA AGT ACC TGG GAT 630
196 F G G S P G G R T L T S T W D 210

631 CCT GCA ACG GGA ACT GTT TCC CAG CGT ATT GTC ACG AAC ACT GAC 675
211 P A T G T V S Q R I V T N T D 225

676 CAC GAC ATG TTC TGC CCC GGT ATC TCA ATG GAC GGT AAC GGC CAG 720
226 H D M F C P G I S M D G N G Q 240

721 ATC GTT GTA ACC GGG GGT AAC GAC GCT CAG AAA ACA TCC TTA TAC 765
241 I V V T G G N D A Q K T S L Y 255

766 GAT TCG TCG TCT GAT TCA TGG ATT CCG GGA CCC GAT ATG AAA GTA 810
256 D S S S D S W I P G P D M K V 270

811 GCG CGT GGC TAT CAG TCG AGT GCG ACT TGC AGT GAC GGA CGT GTC 855
271 A R G Y Q S S A T C S D G R V 285

856 TTT ACG ATT GGG GGG AGT TGG AGT GGC GGC GAG GTG TTC GAG AAG 900
286 F T I G G S W S G G E V F E K 300

901 AAC GGT GAA ATT TAC GAC CCA AGT AGC AAC ACT TGG ACT ATG TTG 945
301 N G E I Y D P S S N T W T M L 315

946 CCC GGT GCC AAA GTT AAA CCG ATG TTG ACC GCC GAT AAA CAG GGG 990
316 P G A K V K P M L T A D K Q G 330

991 GTG TAC CGC GCA GAT AAC CAT GCC TGG CTT TTT GGG TGG AAA AAC 1035
331 V Y R A D N H A W L F G W K N 345

1036 GGT TCG GTG TTC CAG GCG GGG CCT TCG ACG GCG ATG AAT TGG TAT 1080
346 G S V F Q A G P S T A M N W Y 360

1081 TAC ACT TCG GGT AAC GGG AAT GTA AAA TCG GCA GGG AAA CGC CAG 1125
361 Y T S G N G N V K S A G K R Q 375

1126 AGT AAT CGT GGT GAC GAT CCG GAC TCC ATG TGC GGT AAC GCC GTG 1170
376 S N R G D D P D S M C G N A V 390

1171 ATG TAT GAC GCA GTT GCT GGA AAG ATT TTA ACG TTT GGC GGT TCT 1215
391 M Y D A V A G K I L T F G G S 405

1216 CCG GAT TAC CAG GAC AGC GAT GCC ACC ACG AAC GCG CAT ATC ATC 1260
406 P D Y Q D S D A T T N A H I I 420

1261 ACG ATC GGA GAC CCT GGT GCC ACA CCG TCG GTA ACC TTC GCT TCG 1305
421 T I G D P G A T P S V T F A S 435

```
1306  AAT GGC ATG CAT TAT GCT CGT GTA TTT CAT ACT TCT GTT GTG CTT   1350
436    N   G   M   H   Y   A   R   V   F   H   T   S   V   V   L    450

1351  CCC GAT GGC ACG GTG TTC ATC ACC GGC GGT CAA TCT TAC GGG ATT   1395
451    P   D   G   T   V   F   I   T   G   G   Q   S   Y   G   I    465

1396  CCG TTC ACT GAC TCT ACG CCC CAG TTT ACA CCA GAA TTA TAC GAC   1440
466    P   F   T   D   S   T   P   Q   F   T   P   E   L   Y   D    480

1441  CCA GAA CAA GAT ACG TTC GTA GAA CAA GCA CCG AAT TCG ATC GTC   1485
481    P   E   Q   D   T   F   V   E   Q   A   P   N   S   I   V    495

1486  CGC GTA TAC CAT AGC ATC TCG TTG TTA TTG CCC GAT GCT ACG GTT   1530
496    R   V   Y   H   S   I   S   L   L   L   P   D   A   T   V    510

1531  TTC AAC GGT GGT GGG GGA CTG TGT GGT GAC TGC AGT ACA AAC CAC   1575
511    F   N   G   G   G   G   L   C   G   D   C   S   T   N   H    525

1576  TTC GAC GCA CAA ATC TTC ACC CCG TCT TAC TTA TTC AAC GCA GAT   1620
526    F   D   A   Q   I   F   T   P   S   Y   L   F   N   A   D    540

1621  GGT TCG CCC GCC ACC CGT CCT GTA ATC ACA TCG GTG AGT ACA TCC   1665
541    G   S   P   A   T   R   P   V   I   T   S   V   S   T   S    555

1666  AGC GTT AAA GTC GGG GGT CGT CTT ACA ATT ACT ACC GAC TCG GCA   1710
556    S   V   K   V   G   G   R   L   T   I   T   T   D   S   A    570

1711  ATT ACT AGC GCT AGC CTT ATC CGC TAT GGG ACG GCA ACT CAC ACT   1755
571    I   T   S   A   S   L   I   R   Y   G   T   A   T   H   T    585

1756  GTA AAT ACG GAC CAA CGT CGC ATC CCA CTG ACA CTG ACC TCC AGT   1800
586    V   N   T   D   Q   R   R   I   P   L   T   L   T   S   S    600

1801  GGA ACT AAT TCG TAC ACG GCG CAA ATC CCC AAC GAC CCT GGG ATC   1845
601    G   T   N   S   Y   T   A   Q   I   P   N   D   P   G   I    615

1846  GCT CTT CCC GGC TAC TGG ATG CTT TTT GTC ATG AAC TCA AAT GGT   1890
616    A   L   P   G   Y   W   M   L   F   V   M   N   S   N   G    630

1891  GTC CCC AGT GTT GCG AAG ACC ATT AAA GTG ACA CTT TGA   1929
631    V   P   S   V   A   K   T   I   K   V   T   L   *
```

>Node_45

```
1     ATG GCC GCT CCG CCC ATT GGT AAC GCC CGT ATC GAC CGT GCC GGA   45
1      M   A   A   P   P   I   G   N   A   R   I   D   R   A   G    15

46    TGG AAA GTC ACG TGC GAT TCA GAA GAA CCA GGC AAT GAA TGC TCG   90
16     W   K   V   T   C   D   S   E   E   P   G   N   E   C   S    30

91    AAG GCA ATC GAC GGG GAC AAC AAT ACG TTT TGG CAC ACT GCA TAT   135
31     K   A   I   D   G   D   N   N   T   F   W   H   T   A   Y    45
```

136 TCT GCT AAT GGA AAT CCT CCT CCC CCT CAC AAC ATT ACT GTT GAC 180
46 S A N G N P P P P H N I T V D 60

181 ATG GGC TCG ACC CAA AAC GTG AAC GGT CTG TCG GTA CTG CCG CGT 225
61 M G S T Q N V N G L S V L P R 75

226 CAA GAC GGA AAT CAA AAC GGG TGG ATT GCT CGT CAC GAA GTG TAT 270
76 Q D G N Q N G W I A R H E V Y 90

271 GTC TCT ACC GAC GGA ACG AAT TGG GGG GAC CCA GTC GCG ACT GGT 315
91 V S T D G T N W G D P V A T G 105

316 ACG TGG TAC GCC GAT TCT ACT ACT AAA TAT GCA AAC TTC GAA CCG 360
106 T W Y A D S T T K Y A N F E P 120

361 CGT AGC GCG CGT TAT GTC CGT TTA GTC GCC CTG TCG GAG GCG AAC 405
121 R S A R Y V R L V A L S E A N 135

406 GGA AAT CCG TGG ACT TCA ATC GCA GAA CTT AAT GTA TAC AAA GCT 450
136 G N P W T S I A E L N V Y K A 150

451 AAC ACT GAA CCA GCT CCA GCA GCC GGC CTT GGG AAG TGG GGC CCC 495
151 N T E P A P A A G L G K W G P 165

496 ACG ATC GAT TTT CCC ATC GTG CCC GTC GCA GGG GCC GTA GAT CCT 540
166 T I D F P I V P V A G A V D P 180

541 TTG ACA GGC AAG GTT TTA GTA TGG TCG TCT TAC GCC AAC GAT AAT 585
181 L T G K V L V W S S Y A N D N 195

586 TTC GAG GGA AGC CCT GGC GGA CGT ACT CTT ACA TCC ACT TGG GAT 630
196 F E G S P G G R T L T S T W D 210

631 CCG GCA ACC GGA GAT GTA ACT CAG CGT ATC GTC ACA AAC ACG GAC 675
211 P A T G D V T Q R I V T N T D 225

676 CAC GAT ATG TTC TGT CCC GGG ATC TCT ATG GAT GGT AAT GGG CAG 720
226 H D M F C P G I S M D G N G Q 240

721 ATT GTG GTA ACC GGA GGC AAC GAT GCT CAA AAA ACA TCT TTG TAC 765
241 I V V T G G N D A Q K T S L Y 255

766 GAT TCA GCG AGT GAT TCT TGG ATC CCG GGT CCT GAC ATG AAA GTT 810
256 D S A S D S W I P G P D M K V 270

811 GCA CGC GGT TAT CAG TCG TCC GCT ACA TGT TCA GAC GGG CGC GTA 855
271 A R G Y Q S S A T C S D G R V 285

856 TTT ACC ATT GGG GGC TCT TGG TCC GGA GGG GAA GTT TTC GAG AAA 900
286 F T I G G S W S G G E V F E K 300

901 AAC GGA GAA ATC TAT GAC CCT AGT ACG AAT ACG TGG ACG ATG CTT 945
301 N G E I Y D P S T N T W T M L 315

946 CCC GGT GCA AAA GTG AAA CCA ATG TTA ACT GCC GAT AAG CAG GGG 990
316 P G A K V K P M L T A D K Q G 330

991 ATC TAC CGC GCA GAC AAT CAC GCC TGG CTT TTC GGT TGG AAA AAT 1035
331 I Y R A D N H A W L F G W K N 345

1036 GGT AGT GTG TTC CAA GCT GGG CCC TCT ACA GCA ATG AAT TGG TAC 1080
346 G S V F Q A G P S T A M N W Y 360

1081 TAC ACA AGC GGA AAT GGT AAC GTA AAA TCG GCC GGC AAG CGC AAG 1125
361 Y T S G N G N V K S A G K R K 375

1126 TCC AAT CGC GGA GAT GAC CCC GAC AGT ATG TGT GGC AAC GCT GTA 1170
376 S N R G D D P D S M C G N A V 390

1171 ATG TAC GAT GCG GTG GCT GGT AAA ATC CTG ACA TTC GGG GGA AGT 1215
391 M Y D A V A G K I L T F G G S 405

1216 CCA AGC TAC CAG GAC TCC GAT GCG ACT ACA AAT GCT CAT ATC ATC 1260
406 P S Y Q D S D A T T N A H I I 420

1261 ACG ATT GGG GAC CCA GGT GCA ACT CCC TCT GTT ACT TTT GCA TCA 1305
421 T I G D P G A T P S V T F A S 435

1306 AAC GGC ATG CAT TAT GCT CGC GTG TTC CAC ACG TCT GTA GTG CTG 1350
436 N G M H Y A R V F H T S V V L 450

1351 CCC GAT GGA ACA GTC TTC ATC ACG GGG GGC CAA TCA TAC GCC ATT 1395
451 P D G T V F I T G G Q S Y A I 465

1396 CCT TTC ACG GAT TCC ACA CCA CAG TTT ACA CCG GAA CTT TAC GAT 1440
466 P F T D S T P Q F T P E L Y D 480

1441 CCC GAG CAA GAC ACA TTT GTG GAA CAA GCA CCG AAT TCA ATC GTT 1485
481 P E Q D T F V E Q A P N S I V 495

1486 CGC GTC TAC CAC TCA ATC TCG TTG CTG TTA CCT GAC GCC ACC GTC 1530
496 R V Y H S I S L L L P D A T V 510

1531 TTC AAT GGA GGA GGT GGC TTG TGC GGA GAC TGC AAC ACG AAT CAC 1575
511 F N G G G G L C G D C N T N H 525

1576 TTT GAT GCC CAA ATC TTC ACG CCT AGC TAT TTG TTT AAT CGC GAT 1620
526 F D A Q I F T P S Y L F N R D 540

1621 GGG TCT CCT GCC GCT CGC CCC GTA ATC ACG AGT GTC AGC ACT AGC 1665
541 G S P A A R P V I T S V S T S 555

1666 TCT GTA AAA GTG GGC GGT CGC TTG ACG ATT ACG ACT GAC TCG GCT 1710
556 S V K V G G R L T I T T D S A 570

1711 GTA ACC AGT GCG AGC TTG ATC CGC TAC GGG ACC GCG ACA CAT ACA 1755
571 V T S A S L I R Y G T A T H T 585

```
1756  GTG AAC ACA GAC CAA CGT CGC ATT CCT CTG ACT CTG ACA TCA TCA   1800
586    V   N   T   D   Q   R   R   I   P   L   T   L   T   S   S   600

1801  GGT ACG AAT TCC TAC ACG GCT CAA TTA CCT AAC GAC CCG GGT ATC   1845
601    G   T   N   S   Y   T   A   Q   L   P   N   D   P   G   I   615

1846  CTG TTA CCG GGA TAC TGG ATG TTG TTT GTG ATG AAT TCG AAC GGA   1890
616    L   L   P   G   Y   W   M   L   F   V   M   N   S   N   G   630

1891  GTT CCC TCT GTC GCC AAG ACG ATC AAG GTT CTG TTG TAA   1929
631    V   P   S   V   A   K   T   I   K   V   L   L   *
```

>Node_53

```
1     ATG GCT GCA CCT CCA TAC AAC TCT GCA CGT ATT GAT CGC AAT GAT   45
1      M   A   A   P   P   Y   N   S   A   R   I   D   R   N   D   15

46    TGG ACC GTT ACG TGT GAC AGT GAA CAG CCT GGG AAT GAG TGT AGC   90
16     W   T   V   T   C   D   S   E   Q   P   G   N   E   C   S   30

91    AAC GCT ATC GAT GGA GAC AAC AAT ACG TTT TGG CAT TCA GAG TAT   135
31     N   A   I   D   G   D   N   N   T   F   W   H   S   E   Y   45

136   GAC CAA AAC GGG AAT GCC CCT CCT CCT CAC AAC ATC ACT ATT GAC   180
46     D   Q   N   G   N   A   P   P   P   H   N   I   T   I   D   60

181   CTG AAA TCT ACG CAG AAC GTC TCT GGG GTT GCT GTG CTT CCT CGC   225
61     L   K   S   T   Q   N   V   S   G   V   A   V   L   P   R   75

226   CAA GAT GGT AAT CAA AAC GGT TGG ATT GGG CAA CAT GAA GTA TAT   270
76     Q   D   G   N   Q   N   G   W   I   G   Q   H   E   V   Y   90

271   GTA AGT ACA GAC GGG GAG AAC TGG GGC TCG CCG GTA GCC TAC GGA   315
91     V   S   T   D   G   E   N   W   G   S   P   V   A   Y   G   105

316   ACC TGG TGG GAC GAC AGC ACG ACA AAG TTA GCT GCG TTT GAG CCA   360
106    T   W   W   D   D   S   T   T   K   L   A   A   F   E   P   120

361   CGT TCA GCT CGC TAT GTG CGC CTG GTT GCA CTT TCG GAA GCA AAC   405
121    R   S   A   R   Y   V   R   L   V   A   L   S   E   A   N   135

406   GGA AAT CCA TGG ACA TCG ATT GCC GAA TTA AAT ATC TAT GCA GCG   450
136    G   N   P   W   T   S   I   A   E   L   N   I   Y   A   A   150

451   AAT TCT TAT ACT GCC CCA CCC CCC GGC AAG GGA GTT TGG GGT CCA   495
151    N   S   Y   T   A   P   P   P   G   K   G   V   W   G   P   165

496   ACA ATT GAT TTT CCA ATC GTC CCC GTA TCA GCA GCG GTC GAA CCA   540
166    T   I   D   F   P   I   V   P   V   S   A   A   V   E   P   180

541   TCG ACA GGA AAA GTA CTG GTA TGG TCG AGT TAC GCT AAC GAT CAG   585
181    S   T   G   K   V   L   V   W   S   S   Y   A   N   D   Q   195
```

586 TTC GGC GGG ACT CCT GGA GGA AAA ACA CTG ACC AGC ACT TGG GAC 630
196 F G G T P G G K T L T S T W D 210

631 CCA GCG ACA GGT ACG GTC TCC CAA CGT ATC GTG ACG AAC ACG GAG 675
211 P A T G T V S Q R I V T N T E 225

676 CAC GAT ATG TTC TGT CCT GGC ATT TCT ATG GAC GGG AAT GGG CGT 720
226 H D M F C P G I S M D G N G R 240

721 ATC GTG GTC ACT GGC GGA AAC GAT GCA ACG AAG ACG TCG ATC TAT 765
241 I V V T G G N D A T K T S I Y 255

766 GAT TCA TCT AGC GAT TCC TGG ATC TCG GGC CCT CCG ATG AAA GTC 810
256 D S S S D S W I S G P P M K V 270

811 GCT CGC GGG TAC CAA TCC TCT ACT ACG CTG TCT GAT GGG CGC GTA 855
271 A R G Y Q S S T T L S D G R V 285

856 TTT GTA ATT GGT GGC TCG TGG AGT GGA GGG GAG GAA TTT GAT AAG 900
286 F V I G G S W S G G E E F D K 300

901 AAT GGC GAG ATT TAT AAT CCA TCG ACC AAC ACG TGG ACG ATG TTA 945
301 N G E I Y N P S T N T W T M L 315

946 CCC GGT GCA AAA GTG AAG CCA ATG CTG ACG AAT GAC AAA CAG GGC 990
316 P G A K V K P M L T N D K Q G 330

991 CCA TAT CGC GCT GAC AAT CAT GGG TGG TTA TTT GGA TGG AAA AAT 1035
331 P Y R A D N H G W L F G W K N 345

1036 CGC TCT GTA TTT CAA GCC GGA CCC TCG AAG GCT ATG AAT TGG TAC 1080
346 R S V F Q A G P S K A M N W Y 360

1081 TAC GTC GAA GGG AAT GGC AAT GTT ACC TCT GCA GGA AAG CGC CAA 1125
361 Y V E G N G N V T S A G K R Q 375

1126 TCC AAT CGT GGT GAT GAT CCC GAC TCG ATG TCT GGT AAC GCT GTA 1170
376 S N R G D D P D S M S G N A V 390

1171 ATG TTC GAC GCG GTC GCT GGA AAA ATT TTG ACT TTT GGA GGA TCA 1215
391 M F D A V A G K I L T F G G S 405

1216 CCA GAC TAC GAA GAT TCA GAT GCG ACA ACT AAT GCT CAC ATT ATT 1260
406 P D Y E D S D A T T N A H I I 420

1261 ACA ATC GGT GAT CCA GGG GCA ACT CCA TCC GTG GTG AAA GCT GGT 1305
421 T I G D P G A T P S V V K A G 435

1306 AAT GGT ATG CAC TAT GCT CGC GTC TTC CAT ACC TCG GTG GTC TTG 1350
436 N G M H Y A R V F H T S V V L 450

1351 CCG GAC GGC ACC GTA TTC ATT ACG GGG GGT CAG TCA TAT GGA ATC 1395
451 P D G T V F I T G G Q S Y G I 465

```
1396  CCG TTC AAC GAA GAG ACT CCA CAA TTC ACC CCT GAA TTA TAC GAC   1440
466    P   F   N   E   E   T   P   Q   F   T   P   E   L   Y   D    480

1441  CCA GAA ACA GAC ACA TTT GTA GAA CAG GCC CCT AAT AAC ATT GTG   1485
481    P   E   T   D   T   F   V   E   Q   A   P   N   N   I   V    495

1486  CGC GTG TAT CAT TCG ATT AGC TTG TTG CTT CCT GAC GGT ACA GTT   1530
496    R   V   Y   H   S   I   S   L   L   L   P   D   G   T   V    510

1531  CTT AAT GGT GGC GGG GGA TTG TGC GGA AAC TGC TCG GCG AAC CAC   1575
511    L   N   G   G   G   G   L   C   G   N   C   S   A   N   H    525

1576  TAC GAT GCA CAA ATC TTT ACC CCT CCA TAT TTG TTC AAC GCG GAT   1620
526    Y   D   A   Q   I   F   T   P   P   Y   L   F   N   A   D    540

1621  GGA AGT CCA GCA ACG CGT CCA AAA ATT ACT TCC GTC TCG CCA AGC   1665
541    G   S   P   A   T   R   P   K   I   T   S   V   S   P   S    555

1666  TCG GTA AAG GTG GGC GGT CGC TTG ACA ATT ACA ACG GAC TCC GCG   1710
556    S   V   K   V   G   G   R   L   T   I   T   T   D   S   A    570

1711  ATC ACG TCA GCC TCC TTG GTC CGC TAC GGG ACC GCT ACC CAT ACA   1755
571    I   T   S   A   S   L   V   R   Y   G   T   A   T   H   T    585

1756  GTC AAT ACT GAT CAG CGT CGC ATC CCC CTT ACG CTT ACT AGT AGT   1800
586    V   N   T   D   Q   R   R   I   P   L   T   L   T   S   S    600

1801  GGT ACA AAC TCG TAC ACG GTT CAA ATC CCA AAT GAT CCG GGT ATT   1845
601    G   T   N   S   Y   T   V   Q   I   P   N   D   P   G   I    615

1846  GCC CTT CCA GGG TAT TGG ATG TTA TTC GTT ATG AAT TCA AAC GGA   1890
616    A   L   P   G   Y   W   M   L   F   V   M   N   S   N   G    630

1891  GTC CCG TCT GTG GCC AAA ACT ATC AAA GTC ACG CTT TGA   1929
631    V   P   S   V   A   K   T   I   K   V   T   L   *

>SarLacc
1     ATG GCC GCT CCG CCC ATT GGT AAC GCC CGT ATC CCT CGT GAC GGA   45
1      M   A   A   P   P   I   G   N   A   R   I   P   R   D   G    15

46    TGG AAA GTC ACG TGC GAT TCA GAA GAA CCA GGC AAT GAA TGC TCG   90
16     W   K   V   T   C   D   S   E   E   P   G   N   E   C   S    30

91    AAG GCA ATC GAC GGG GAC AAC AAT ACG TTT TGG CAC ACT GCA TAT   135
31     K   A   I   D   G   D   N   N   T   F   W   H   T   A   Y    45

136   TCT GCT AAT GGA AAT CCT CCT CCC CCT CAC AAC ATT ACT GTT GAC   180
46     S   A   N   G   N   P   P   P   P   H   N   I   T   V   D    60

181   ATG GGC TCG ACC CAT AAC GTG AAC GGT CTG TCG GTA CTG CCG CGT   225
61     M   G   S   T   H   N   V   N   G   L   S   V   L   P   R    75
```

226 CAA GAC GGA AAT CAA AAC GGG TGG ATT GCT CGT CAC GAA GTG TAT 270
76 Q D G N Q N G W I A R H E V Y 90

271 GTC TCT ACC GAC GGA ACG AAT TGG GGG GAC CCA GTC GCG TCA GGT 315
91 V S T D G T N W G D P V A S G 105

316 ATG TGG TAC GCC GAT TCT ACT ACT AAA TAT GCA AAC TTC GAA CCA 360
106 M W Y A D S T T K Y A N F E P 120

361 CGT AGC GCG CGT TAT GTC CGT TTA GTC GCC CTG TCG GAG GCG AAC 405
121 R S A R Y V R L V A L S E A N 135

406 GGA AAT CCC TGG ACT TCA ATC GCA GAA CTT AAT GTA TAC AAA GCT 450
136 G N P W T S I A E L N V Y K A 150

451 AAC ACT GAA CCA CCG CCA GCA GCC GGC CTT GGG AAG TGG GGC CCC 495
151 N T E P P P A A G L G K W G P 165

496 ACG ATC GAT TTT CCC ATC GTG CCC GTC GCA GGG GCC GTA GAT CCT 540
166 T I D F P I V P V A G A V D P 180

541 TTG ACA GGC AAG GTT TTA GTA TGG TCG TCT TAC GCC AAC GAT CAG 585
181 L T G K V L V W S S Y A N D Q 195

586 TTC GAG GGA AGC CCT GGC GGA CGT ACT CTT ACA TCC ATC TGG GAT 630
196 F E G S P G G R T L T S I W D 210

631 CCG GCA ACC GGA GAT GTA ACT CAG CGT ATC GTC ACA AAC ACG GAC 675
211 P A T G D V T Q R I V T N T D 225

676 CAC GAT ATG TTC TGT CCC GGG ATC TCT ATG GAT GGT AAT GGG CAG 720
226 H D M F C P G I S M D G N G Q 240

721 ATT GTG GTA ACC GGA GGC AAC GAT GCT CAA AAA ACA TCT TTG TAC 765
241 I V V T G G N D A Q K T S L Y 255

766 GAT TCA GCG AGT GAT TCT TGG ATC CCG GGT CCT GAC ATG AAA GTT 810
256 D S A S D S W I P G P D M K V 270

811 CCA CGC GGT TAT CAG TCG TCC GCT ACA CTA TCA GAC GGG CGC GTA 855
271 P R G Y Q S S A T L S D G R V 285

856 TTT ACC ATT GGG GGC TCT TTC TCC GGA GGG GAA GTT TTC GAG AAA 900
286 F T I G G S F S G G E V F E K 300

901 AAC GGA GAA ATC TAT GAC CCT AGT ACG AAT ACG TGG ACG CTA CTT 945
301 N G E I Y D P S T N T W T L L 315

946 CCC GGT GCA AAA GTG AAA CCA ATG TTA ACT AAC GAT AAG CAG GGG 990
316 P G A K V K P M L T N D K Q G 330

991 ATC TAC AAG GCA GAC AAT CAC GCC TGG CTT TTC GGT TGG AAA AAT 1035
331 I Y K A D N H A W L F G W K N 345

1036 GGT AGT GTG TTC CAA GCT GGG CCC TCT ACA GCA ATG AAT TGG TAC 1080
346 G S V F Q A G P S T A M N W Y 360

1081 TAC ACA AGC GGA AAT GGT AAC GTA AAA TCG GCC GGC AAG CGC AAG 1125
361 Y T S G N G N V K S A G K R K 375

1126 TCC AAT CGC GGA GAT GAC CCC GAC AGT ATG TGT GGC AAC GCT GTA 1170
376 S N R G D D P D S M C G N A V 390

1171 ATG TAC GAT GCG GTG GCT GGT AAA ATC CTG ACA TTC GGG GGA AGT 1215
391 M Y D A V A G K I L T F G G S 405

1216 CCA AGC TAC ACG GAC TCC GAT GCG ACT ACA AAT GCT CAT ATC ATC 1260
406 P S Y T D S D A T T N A H I I 420

1261 ACG ATT GGG GAC CCA GGT GCA ACT CCC TCT GTT ACT TTT GCA TCA 1305
421 T I G D P G A T P S V T F A S 435

1306 AAC GGC ATG TGG TAT GCT CGC GTG TTC CAC ACG TCT GTA GTG CTG 1350
436 N G M W Y A R V F H T S V V L 450

1351 CCC GAT GGA ACA GTC TTC ATC ACG GGG GGC CAA TCA TAC GCC ATT 1395
451 P D G T V F I T G G Q S Y A I 465

1396 CCT TTC ACG GAT TCC ACA CCA CAG TTT ACA CCG GAA CTT TAC GAT 1440
466 P F T D S T P Q F T P E L Y D 480

1441 CCC GAG CAA GAC ACA TTT GTG GAA CAA GCA CCG AAT TCA ATC CCT 1485
481 P E Q D T F V E Q A P N S I P 495

1486 CGC GCC TAC CAC TCA GTA TCG TTG CTG TTA CCT GAC GCC ACC GTC 1530
496 R A Y H S V S L L L P D A T V 510

1531 TTC AAT GGA GGA GGT GGC TTG TGC GGA GAC TGC AAC ACG AAT CAC 1575
511 F N G G G G L C G D C N T N H 525

1576 TTT GAT GCC CAA ATC TTC ACG CCT CCT TAT TTG TTT GAT CGC GAT 1620
526 F D A Q I F T P P Y L F D R D 540

1621 GGG TCT CCT GCC GCT CGC CCC GTA ATC ACG AGT GTC AGC ACT GAT 1665
541 G S P A A R P V I T S V S T D 555

1666 TCT GTA AAA GTG GGC GGT CGC TTG ACG ATT CAA ACT GAC TCG CCG 1710
556 S V K V G G R L T I Q T D S P 570

1711 GTA ACC AGT GCG AGC TTG ATC CGC TGT GGG ACC GCG ACA CAT ACA 1755
571 V T S A S L I R C G T A T H T 585

1756 GTG AAC ACA GAC CAA CGT CGC ATT CCT CTG ACT CTG ACA TCA TCA 1800
586 V N T D Q R R I P L T L T S S 600

1801 GGT ACG AAT TCC TAC ACG GCT CAA TTA CCT AAC GAC CCG GGT ATC 1845
601 G T N S Y T A Q L P N D P G I 615

```
1846  CTG TTA CCG GGA TAC TGG ATG TTG TTT GTG ATG AAT TCG AAC GGA   1890
616    L   L   P   G   Y   W   M   L   F   V   M   N   S   N   G   630

1891  GTT CCC TCT GTC GCC AAG ACG ATC AAG GTT CTG TTG TGA   1929
631    V   P   S   V   A   K   T   I   K   V   L   L   *
```
